# The concepts of irreversibility and reversibility in research on anthropogenic environmental changes

**DOI:** 10.1093/pnasnexus/pgae577

**Published:** 2024-12-31

**Authors:** Lorina Buhr, Dominic S Lenzi, Auke J K Pols, Claudia E Brunner, Andrea Fischer, Arie Staal, Benjamin P Hofbauer, Bernice Bovenkerk

**Affiliations:** Department of Philosophy, Institute for Liberal Arts & Sciences, University of Hamburg, 20146 Hamburg, Germany; Department of Philosophy and Religious Studies, Ethics Institute, Utrecht University, 3512 BL Utrecht, Netherlands; Faculty of Behavioural, Management and Social Sciences, University of Twente, 7522 NB Enschede, Netherlands; Knowledge, Technology and Innovation Group, Section CPTE (Communication, Philosophy, Technology, and Education), Wageningen University & Research, 6706 KN Wageningen, Netherlands; Max Planck Institute for Dynamics and Self-Organization, 37077 Göttingen, Germany; Institute for Interdisciplinary Mountain Research, Austrian Academy of Sciences, 6020 Innsbruck, Austria; Copernicus Institute of Sustainable Development, Utrecht University, 3584 CB Utrecht, Netherlands; Faculty of Technology, Policy and Management, Delft University of Technology, 2628 BX Delft, Netherlands; Research Institute for Sustainability – Helmholtz Centre Potsdam, 14473 Potsdam, Germany; Philosophy Group, Section CPTE (Communication, Philosophy, Technology, and Education), Wageningen University & Research, 6706 KN Wageningen, Netherlands

**Keywords:** reversibility, irreversibility, climate change, tipping point, ecosystem degradation

## Abstract

The concept of “irreversibility” and its counterpart “reversibility” have become prominent in environmental and ecological research on human-induced changes, thresholds, climate tipping points, ecosystem degradation, and losses in the cryosphere and biosphere. Through a systematic literature review, we show that in these research fields, these notions are not only descriptive terms, but can have different semantic functions and normative aspects. The results suggest that, in the context of environmental and ecological research the concepts of irreversibility and reversibility have taken on additional usages in comparison to their contexts in theoretical thermodynamics and mechanics. Irreversible as a classification of anthropogenic environmental change can be used categorically, in the sense of a finite end, or relatively, i.e. on time or spatial scales of interest. Surprisingly, most of the analyzed scientific articles that use the terminology of (ir)reversibility substantively do not provide an explicit conceptualization or definition (74.7%). The research on potential (ir)reversibility of environmental change may affect the social and political willingness to bear the costs of interventions to mitigate or prevent undesirable environmental change. In particular, classifying a change as reversible or irreversible and determining the timescale(s) and spatial scale(s) involved has implications for policy and ecosystem management decisions, as suggested by its use in several high-level scientific and policy reports on ecosystem and climate change. Therefore, it is important to explicitly present a clear definition of irreversibility or reversibility for the readers from other fields, even if it could be the case that within a specific community an implicit definition was considered to be sufficient. We propose further recommendations for inter- and transdisciplinary reflection and conceptual use in the context of environmental, ecological, and sustainability research.

## Introduction

Starting in 2021 and led by the United Nations Environment Programme and the Food and Agriculture Organization of the United Nations, the main target of the current “UN Decade on Ecosystem Restoration” is “to prevent, halt and reverse the degradation of ecosystems worldwide” (1). Here, ecological restoration is based on the assumption that the degradation of ecosystems is, at least to some extent, *reversible*. Meanwhile, the latest Intergovernmental Panel on Climate Change (IPCC) Synthesis Report (AR6) highlights and warns of “*irreversible* changes and losses”, including species extinction, the retreat and disappearance of mountain glaciers and substantial changes in the global water cycle and ocean circulation (2). The UN report on Global Sustainable Development 2023, dedicated to “Times of Crisis, Times of Change: Science for Accelerating Transformations to Sustainable Development” lists “[o]verharvesting of species, agricultural activities, logging and deforestation for agriculture” as processes “causing *irreversible damage* to the world's biodiversity” (3). In environmental and ecological research, reversibility and irreversibility are often used in close connection with the popular terminology of thresholds, such as “critical transitions”, Earth, climate and environmental “tipping points”, “catastrophic shifts and bifurcations”, “regime shifts” in and of ecosystems, “abrupt changes”, “hysteresis” (4–9), and “collapse”. Paradigmatically, the Global Tipping Points Report 2023 defines tipping points “as occurring when change in part of a system becomes self-perpetuating beyond a threshold, leading to substantial, widespread, frequently abrupt and often irreversible impact” (10). However, even before the rise of threshold terminology, the use of the term “threshold” was fraught with “ambiguity” and “a desire for simplicity” in ecological and environmental change and management literature, potentially leading to uncritical uses lacking empirical evidence or determination of spatial and temporal scales especially when applied in contexts of managerial decision-making (11). The risk of vagueness or misunderstandings in the use of threshold terminology, including the concept of tipping points, and the disconnect between theoretical frameworks of nonequilibrium dynamics and their application in ecosystem management have been highlighted repeatedly (11–20). Previous reviews and analysis of terminology used in environmental and socio-ecological systems literature have primarily focused on the concepts of thresholds or tipping points (12, 20, 21), regime shift (22) or resilience (23). Yet, in a prominent review study by Manjana Milkoreit and colleagues (12), irreversibility—and the related “limited reversibility”, i.e. hysteresis—was identified as one of four “necessary (and potentially sufficient) conditions” in approaches to defining and understanding tipping points across disciplines. However, reversibility and irreversibility tend to remain under-conceptualized in the environmental and ecological literature (24). Nor have the usages of the concepts (linguistically, both the noun and the adjectives “irreversible” and “reversible”) been the subject of a comprehensive investigation. In response, we conducted a systematic literature review study on the usages and functions of the concept of irreversibility, and its conceptual companion reversibility, in natural scientific articles on anthropogenic environmental changes. We also tracked the normative dimensions involved, and the roles they might play in policy-oriented argumentation in the face of climate change and the biodiversity crisis. We define anthropogenic environmental changes as those caused by human activity or resulting from environmental changes that are at least partly induced by human activity, including interventions brought about by technology, labor, mining, urbanization, and agricultural practices. In our systematic review, we identified, screened, and analyzed scientific articles from the environmental, geological, Earth system, and biological sciences, including subdisciplines and interdisciplinary research fields such as agricultural or forestry ecology or ecological restoration. We were primarily interested in current research on anthropogenic changes to the environment and ecosystems that did not have a particular anthropocentric or managerial focus, such as human well-being and societal development. For this reason, work based on a social–ecological systems framework was not included. Based on the findings, we propose recommendations for inter- and transdisciplinary reflection on irreversibility and reversibility in the context of anthropogenic environmental change and decision-making for sustainable futures.

## Results

We included 996 articles in the full-text screening, of which 91 show substantive use of either irreversibility, reversibility, or both in the target research fields (see for an overview of selection procedure and a syllabus of the 91 included articles with substantive use of the target concepts Box 1; Supplementary Material Reference List S1; Dataset S1).

Box 1. **Decision scheme that was applied for the selection of articles.**

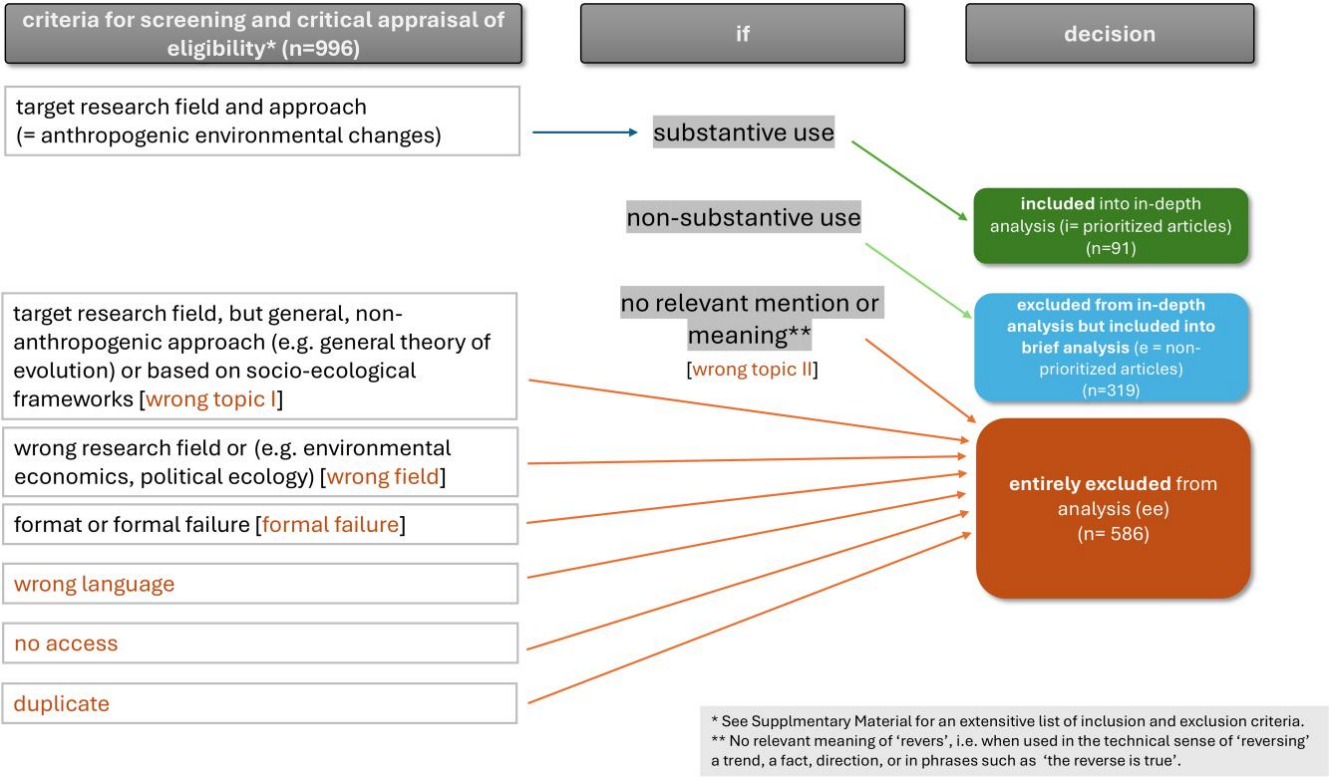

### Substantive conceptual use

**Fig. 1.**
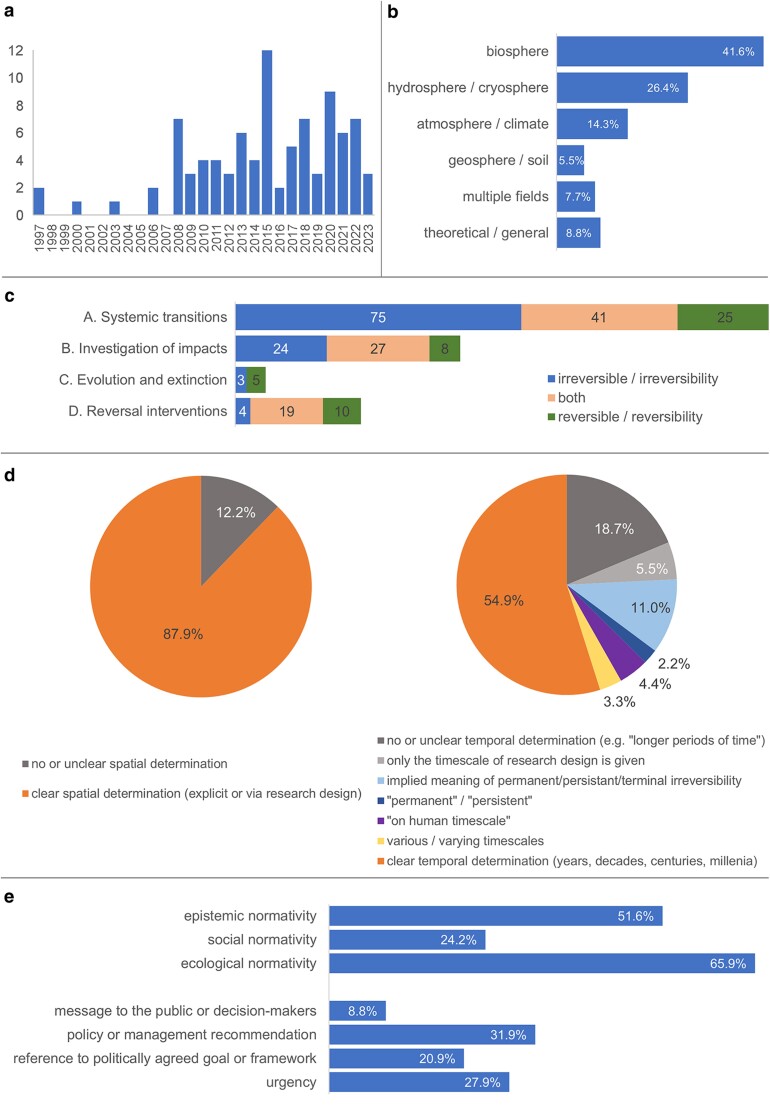
Quantitative analysis of articles with substantive use of the target concepts. a) Distribution of included articles across years of publishing (1997–2023) (*n* = 91). b) Distribution of thematic domains that were primarily addressed by the included articles, six papers showed two primary domains. c) Distribution of conceptual foci per subject area and research topic (cases). We differentiated between three conceptual foci: (i) irreversible/irreversibility used as one or as the key concept or category; (ii) reversible/reversibility used as one or as the key concept or category; (iii) irreversible/irreversibility and reversible/reversibility are used together as one or the key category for research. As a next step, the conceptual foci were investigated with regard to the subject areas. d) Proportion of articles specifying the concepts of reversible/reversibility or/and irreversible/irreversibility in temporal and spatial terms (*n* = 91). e) Proportion of articles entailing normative statements and references to politically agreed goals or frameworks and phrases conveying urgency (*n* = 91).

**Fig. 2.**
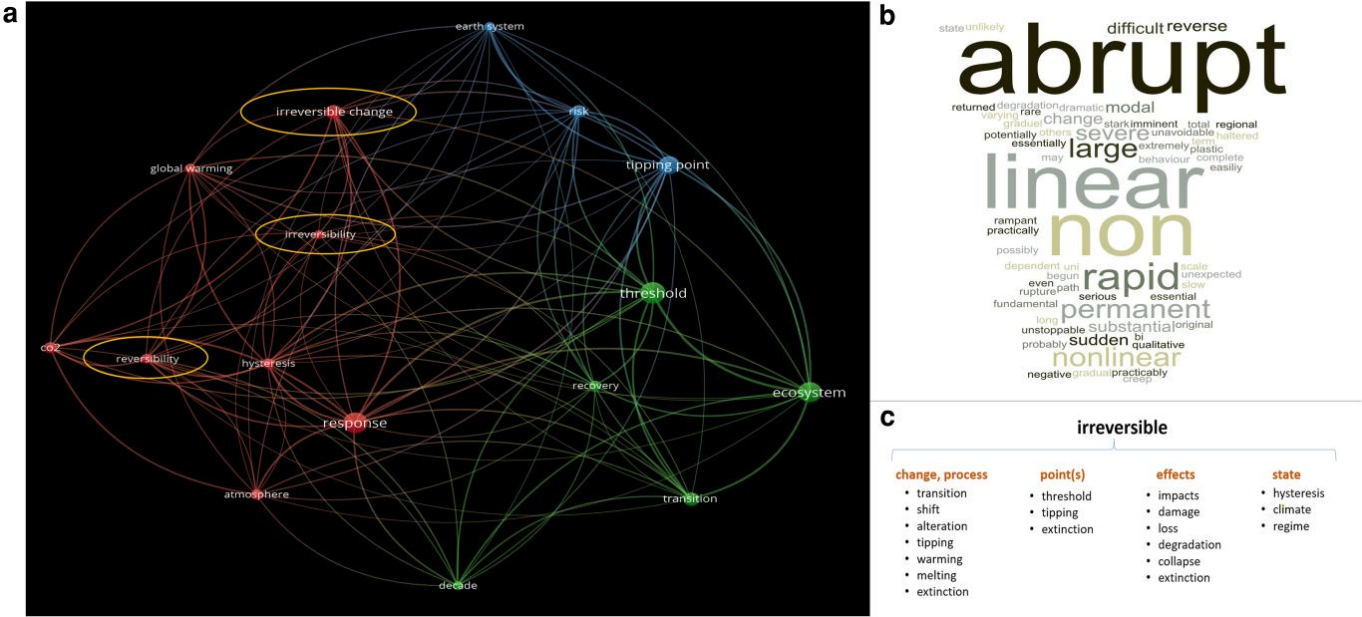
Qualitative and bibliometric analysis of articles showing substantive use of (ir)reversibility (*n* = 91). a) Bibliometric analysis of the most relevant terms used in the titles and abstracts provided by VOSviewer 1.6.20, methods used: binary counting, minimum number of occurrences 9, whereof 60% (*n* = 18) less 2 manual deletions (“example”, “point”) were included in the graph (*n* = 16), total link strength: 30. b) World cloud of attributes used in the close context of irreversibility/irreversible extracted by coding. c) Scheme of attribution: the notion “irreversible” is attributed to a range of subjects as revealed by the coding and analysis.

All articles included on the basis of substantive use of the concepts irreversibility and reversibility fall within the period 1997 to 2023 (see Fig. 1a). Most articles deal with changes in the biosphere (41.6%), followed by research on the cryosphere and hydrosphere (26.4%). The concepts are used much less in research on the atmosphere and climate (14.3%) and on the lithosphere (5.5%) (see Fig. 1b). Interestingly, the notion of irreversibility is used to qualify several things, namely environmental and ecosystem changes (e.g. shifts, transitions) in general, points of changes, effects or consequences of changes, and the states involved in changes (see Fig. 2c). Terms used in association with irreversibility/irreversible indicate the nature/type of system response relative to the forcing applied on it (abrupt, sudden), the quality or type (nonlinear, severe, rapid), and the time scale (long-term; centuries to millennia) of change (see Fig. 2b). Bibliometric analysis of author networks, and related concepts and keywords, showed, as expected, that many of the seminal authors working on climate tipping points, ecological thresholds and regime shifts emerged as prominent reference nodes. Thereby, the author connection landscape and co-authorship network are rather decentralized, showing that research on (ir)reversibility is not limited to a specific set of disciplines or theoretical frameworks (see Fig. S1a, b, f).

### Subject areas and topics

We identified four main subject areas where the concepts of irreversibility and reversibility are used: A) Systemic transitions, B) Investigation of Impacts (impact studies), C) Evolution and Extinction, D) Scenarios of Reversal Intervention. Within the subject areas, we clustered the research themes where possible around topics. For A), these are (i) ecological thresholds/regime shifts/tipping points, (ii) Earth system thresholds/regime shifts/tipping points, (iii) general approaches to environmental changes. For B), these are (i) impacts of greenhouse gas emissions, (ii) impacts as consequences of climate change, and (iii) direct impacts of human activity; for D) (i) biological conservation, (ii) ecosystem restoration and recovery, and (iii) climate engineering (see Table 1a, see also the extended version in Table S1). It is noteworthy that some articles focused primarily on the notion of irreversibility, others on reversibility and many used both notions together in the function of a category, which we propose to call “ir/reversibility” (see Fig. 1c). The conceptual focus on irreversibility is considerably higher than on reversibility in the subject areas of systemic transitions and impact studies (75 versus 25 times and 24 versus 8 times). The opposite is true for the subject area of reversal intervention, such as reversing global warming to reduce sea level rise, removing carbon dioxide from the atmosphere, and restoring ecosystems, where the conceptual focus is much more on reversibility (see Fig. 1c). The wide range of topics was confirmed by a bibliometric analysis of the titles, abstracts, and keywords as assigned by metadata. The bibliometric analysis also showed clearly that the notion of reversibility has emerged as a stand-alone concept which occurs alongside questions of potentiality or feasibility of the reversal of change, damage or undesirable states (see Fig. S1f). As expected, seminal authors in the research fields of ecological and environmental thresholds such as Tim M. Lenton, Oliviér Boucher, Rikarda Winkelmann, and Susan Solomon are key figures in the co-authorship networks (see Fig. S1a to e).

**Table 1.** a) Substantive terminological usages across subject areas and topics (*n* = 91), short version. Some articles covered more than one topic (up to three topics per article found, *n* = 26), we counted each article once per topic. b) Conceptual functions. Some articles covered more than one conceptual function (up to four functions/usages per article found, *n* = 78), and/or more than one conceptual focus (up to three foci per article found, *n* = 4). We counted each article once per function, regardless of the number of topics covered by the function. See for an extended version of this table in Table S1.

a) Subject areas and topics	%	b) Conceptual functions	%
A. Systemic transition	66.3	Conceptual innovation	12.1
** **A.1 Ecological thresholds/regime shifts/tipping points	28.2	Definiendum	25.3
** **A.2 Earth system tipping points/elements/threshold behavior	19.9	Definiens	7.7
** **A.3 General approaches to environmental changes	18.2	Definitional extension (e.g. in definitions of tipping points)	14.3
B. Impacts	29.0	One or the key analytical criterion or research	54.9
** **B.1 Impacts of greenhouse gas emissions	13.6	One or the key notion in the description or classification of research findings:	
** **B.2 Impacts as consequences of climate change	6.3	** **empirical (field study; monitoring; experiment)	27.5
** **B.3 Direct impacts of human activity	9.1	** **modeling/simulation (incl. conceptual models)	34.1
C. Evolution and extinction	4.5	** **review; meta-analysis; assessment	18.7
D. Scenarios of reversal intervention	16.3		
** **D.1 Biological conservation	0.9		
** **D.2 Ecosystem restoration, recovery	5.4		
** **D.3 Climate engineering	10.0		

### Conceptual functions

We found that the concepts of irreversible/irreversibility and reversible/reversibility are used in different ways, serving five main functions (see Table 1b). First, a definition or clarification of irreversibility and reversibility is presented (then they are used as *definiendia*). Second, irreversibility and reversibility serve as a component in a definition (i.e. as a *definiens*), such as in the definition of ecosystem degradation; or, third, they provide a further explication of a definition (i.e. *definitional extension)*, for example an amendment of the definition of tipping points such as that “they are abrupt and/or irreversible,” or in specifying that some tipping points are reversible but others irreversible. Fourth, irreversibility and reversibility are used as one or as the key *analytical criterion* of a study, and fifth, the notions are used in *describing and classifying the research findings* of empirical, modeling, or meta-analysis studies. In these cases, the notions are often part of the research questions (“Is the decline of the Greenland Ice Sheet reversible?” [211]; “Are there critical limits to the duration and/or magnitude of an overshoot beyond which (aspects of) climate change become irreversible?” [996]) or hypotheses that guide the study (see also Supplementary Material Reference List S1). When coupled in the terminological pair irreversible-reversible the notions serve as one or as the key category in research design and/or findings. For example, the notions are used as category in conceptual frameworks or typologies of ecological changes [995], such as a framework of grassland-to-woodland transitions, which is “used to differentiate between state transitions that are truly irreversible versus those that are hysteretic in experimental restoration of grassland from an alternative juniper woodland state” [520, p. 2]. In some cases (*n* = 11), the notion of irreversibility was integrated into new research notions such as “irreversibility potential”, “threshold of irreversibility”, or “hotspots of irreversibility” showing some conceptual innovation (see Table 2; Table S2). In more than half of the articles (54.9%), irreversibility or reversibility is used as one or the key analytical criterion or research category, and for a considerable number of articles the notions are used to classify research findings (18.7 to 34.1%). Against this backdrop it is surprising to see that only one quarter of the articles provide a definition of (ir)reversibility (25.3%) (Table 1b).

**Table 2.** Selection of definitions and conceptual innovations using irreversibility or reversibility as found in the articles with substantive terminological use. The references are included in the Supplementary Material Reference List S1.

No.	Reference	Definition
	IPCC AR5, glossary	“A perturbed state of a dynamical system is defined as **irreversible** on a given timescale, if the recovery timescale from this state due to natural processes is substantially longer than the time it takes for the system to reach this perturbed state. In the context of this report, the time scale of interest is centennial to millennial. See also Tipping point.”
	IPCC AR6, glossary	“A perturbed state of a dynamical system is defined as **irreversible** on a given time scale if the recovery from this state due to natural processes takes substantially longer than the time scale of interest. See also: Tipping point.”
102	Kim et al. (2022)	“The ability of the climate system to be restored to its initial state is referred to as **reversibility**.” (p. 834) “**Reversibility** of a system can be measured as whether the trajectory return to its initial state, indicated as an open loop (irreversible change) and closed loop (reversible change) […].” (p. 835) “an open-loop trajectory does not always indicate that a system is **completely irreversible**. Even if the loop is open, there is a possibility that the system will return to its initial state if sufficient time is provided after the forcing reaches the initial level. Nevertheless, at least, they show that the climate system cannot be immediately restored to its initial state even after successful removal of the atmospheric CO2. The **soft definition of irreversibility** provides a practical classification for climate recoverability within a human-perceptible timescale.” (p. 835)
298	Yaron et al. (2010)	“By **irreversible changes**—on a human time scale—we refer to long-term, stable, and persistent transformations of subsurface structure and properties, which are also resistant to remediation procedures and to natural attenuation.” (p. 2)
119	Lenton (2014)	“Tipping point change also includes transitions that are slower than their cause (in both cases the rate is determined by the system itself). In either case the **change** in state may be reversible or irreversible. **Reversible** means that when the forcing is returned below the tipping point the system recovers its original state (either abruptly or gradually). **Irreversible** means that it does not (it takes a larger change in forcing to recover). Reversibility in principle does not mean that changes will be **reversible in practice**.” (p. 25–6)
527	Boucher et al. (2012)	“**Irreversibility** means that the system cannot be restored to its initial state or only does so on a timescale far longer than those normally considered practical from a human perspective.” (p. 2)
996	Schwinger et al. (2022)	“We define ‘**reversibility’** based on a reference pathway without overshoot (i.e. no CDR applied) and based on cumulative carbon emissions (i.e. the overshoot simulations have the same amount of cumulative carbon emissions after CDR than the reference pathway).” (p. 1641) “We define an aspect of the Earth system to be **reversible** through the application of CDR if the mean state after an overshoot is within the internal variability of the reference case without overshoot. We stress that this definition neither implies reversibility in the absence of CDR nor reversibility of climate change that is committed to in the reference scenario.” (p. 1656)

		Conceptual Innovation
56	Good et al. (2018)	Key finding: “Some **degree of irreversible loss** may have begun, although the eventual magnitude and rate of this irreversible loss is uncertain.” (p. 27)
377	Swingedouw et al. (2020)	“**Irreversibility potential of change**” (used as a categorial qualifier for assessed tipping elements, e.g. the potential is valid, probable, possible, unlikely, varying)
526	Botero et al. (2015)	“We label this strategy **irreversible plasticity** because individuals in these populations exhibit plasticity exclusively during development. The transition from reversible to irreversible plasticity occurs at progressively shorter timescales in less predictable environments because the expected benefits of phenotypic adjustment decrease with higher potential for errors in anticipating environmental change.” (p. 185)
870	Rosier et al. (2021)	“Hereafter we will refer to the former as **irreversible**, in line with previous studies, and the latter as **permanently irreversible**, to differentiate the two. Diagnosing whether a tipping point has been crossed without some prior knowledge of the system is not generally possible without reversing the forcing to see if hysteresis has occurred.” (p. 1502)
967	Mondal et al. (2023)	“The exposed population is prominent in South Africa and Asia. Notably, the population change effect is the principal factor in global exposure change, while it is the climate change effect over the **hotspots of irreversibility**.”

### Temporal and spatial determination

Many articles characterize irreversibility and reversibility on decadal, centennial, or millennial timescales—depending on the change and the entity under consideration (54.9%). These timescales are usually referred to as biological, ecological, or geological timescales. Two of the most influential studies on climate tipping points qualify these timescales relative to what society should or could reasonably decide on, as they distinguish between an “ethical time horizon” (1,000 or 10,000 years) and a “political time horizon” (decades, <100 years) [21; 126]. However, 18.7% of the articles give no or unclear specification of irreversibility or (ir)reversible transitions or remain rather vague, using approximate terms such as “permanent” or “persistent” (2.2%), “on human timescales”, “on timescales relevant to society” (4.4%), or convey an implicit meaning of permanent change (11%) (see Fig. 1d; Table S3). Notably, whereas the Earth system and climate science literature tends to define irreversibility/irreversible change on timescales and spatial scales relevant to human society, the ecology literature tends to be vague in terms of temporality (see Table S3). The spatial scales at stake were in most cases explicitly delineated or implicitly given by the research design or the specific ecosystem under investigation (87.9%; see Fig. 1d). We can thus observe two senses of irreversibility. The first can be described as “relative”, i.e. when irreversibility is defined and specified in relation to human concerns (e.g. irreversible within human timescales, but potentially reversible beyond them) (25). The second is when irreversibility is used in the sense of a “categorical” or “terminal” event or process, where no reversal is technically or ontologically possible, such as extinction, total collapse, or heavy water pollution.

### Illustration and theoretical frameworks of (ir)reversibility

**Fig. 3.**
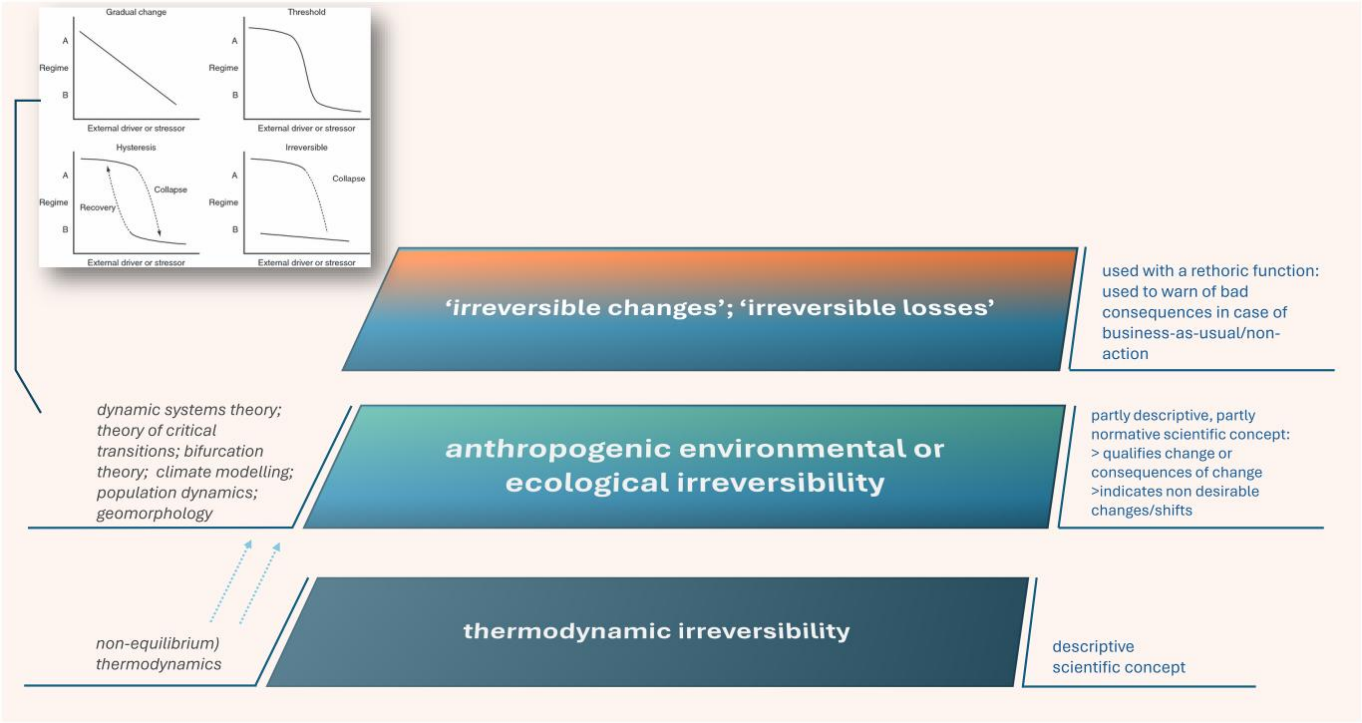
Conceptual layers and aspects of the concept of irreversibility used in different contexts, based on varying theoretical frameworks. Many uses of irreversibility in the context of anthropogenic environmental or ecological change research are based on dynamic systems theory as the underlying theoretical framework, which in turn have generated conceptual models as shown top left. The top left integrated figure is from Davis et al. (26 WILEY, Freshwater Biology), based on Gordon et al. (15).

We explored whether and how irreversibility and reversibility were depicted visually in the articles. More than a third of the articles (39.6%) (see Supplementary Material Reference List S1) used schematic representations of ecosystem changes or possible response behavior towards perturbations. Mostly, variations of equilibrium curve diagrams and stability landscapes (ball-in-basin schemes) were used. These types of figures have become prominent in ecology due to the seminal article on critical transitions by Scheffer et al. (6) (see Fig. S1c,d; S2). The schemes synthesize several technical key concepts of the underlying theoretical framework of dynamical systems theory—an area of mathematics closely related to chaos theory and deploying bifurcation theory—such as equilibrium, stability, (multiple) states, alternative equilibria, stable dynamic equilibrium, hysteresis, basin of attraction, and catastrophic bifurcation. With their help, the concept of resilience is operationalized (e.g. as the “valley” or “basin of attraction” of system states). Notably, conceptual critical transition schemes mainly build on “revers-terminology” to qualify change and perturbation conditions, i.e. they use terms and phrases such as reversal, “difficult-to-reverse”, reversibility, and recovery. The influential paper on ecological regime shifts by Gordon et al. (15) extends the typical three-type typology (linear change, reversible change, critical transition showing hysteresis) by the type “irreversible changes” as a stronger form of hysteresis, where no backward shift after equilibrium collapse, i.e. recovery, is possible [995] (see Fig. 3). It is noteworthy that depending on the discipline and subject area, the use of (ir)reversibility implicitly or explicitly draws from a variety of physical and mathematical theoretical frameworks including nonlinear dynamics and thermodynamics (see Fig. 3; Supplementary Material Reference List S1).

### Types of normativity

We found that the articles show several levels of normativity. Broadly speaking, normativity can be understood as referring to or expressing an “ought” or norm (27). In a basic sense, this means a descriptive reference to a systemic or social norm; in a stronger sense, it contains an attitude or action-guiding claim (28) or advances an ethical or political recommendation. Normative statements can be implicit and explicit. We coded and grouped the normative statements in the articles, and, based on the material, we created a typology of normative statements (see Table S5a). We identified five types of normative statements entailing five types of normativity: (a) “Ecological” or “systemic normativity” concerns an ecosystem when it is subject to changes in its functionality, structure, and/or composition, which is reflected in terms such as “degradation”, “species loss”, “reduction”, or “decline”. Irreversible change or the expected new state of an ecosystem can also be *evaluated,* e.g. in terms of impacts on human society or ecosystems. This is expressed through the use of terms such as “desirable”, “negative effects/impacts on”, “nondesirable”, “harmful” states of ecosystems. Some articles leave the subject of evaluation open. (b) “Social normativity” concerns harms to human well-being, societies, or economies when ecosystems are irreversibly degrading, or climate tipping elements are expected to cross critical thresholds. Compared to ecological normativity, social normative statements express explicitly negative impacts on human societies. (c) “Epistemic normativity” shows up when research findings are explicitly designated as valuable knowledge for ecosystem management or policy, in other words, research findings *should* inform managerial, societal, or political decision-making. The fourth type of normative statements (d) includes explicit ethical or policy recommendations for ecosystem management, climate action and sustainable development facing fundamental ecosystem change (“policy normativity”). Additionally, we identified a fifth type of normative statement (e), which appears as a general message to the public and implies “paradigmatic normativity” (29). It includes statements about rights and wrongs in the way issues of anthropogenic change are discussed or viewed and refers to an “ought” in the sense of how issues and problems should (not) be better discussed and understood; a specific actor is not necessarily addressed. An example is: “These and other management efforts must confront a new reality that promoting the recovery of ecosystems to historical conditions will not be easy, or even possible, as biophysical conditions rapidly change (Harris and others 2006).” ([167], p. 751). More than half of the articles make explicit normative claims about how to use the knowledge presented, and about one-third of the articles made proposals of what action is needed or should be taken; see Fig. 1e for the distribution of normative statements and references.

### Nonsubstantive usage of the notion of (ir)reversibility

A considerable proportion of the screened articles in the relevant disciplines showed nonsubstantive usage of (ir)reversibility, i.e. the notions were used without further elaboration or significance for the research presented in the article (*n* = 319) (Supplementary Material Reference List S2; Dataset S2). The brief analysis revealed that the majority of these articles showed a lack of conceptual specification (65.3%), yet more than a third contained a warning (38.1%) or urgency (10.6%) statement.

## Discussion

The terminology of critical transitions is at the forefront of environmental research. The concepts of irreversibility and reversibility are related to this terminology, but as demonstrated in this review analysis, have their own range of semantic meaning and functionality. In the following, we discuss the status of these concepts in research on anthropogenic environmental change.

### Irreversibility and reversibility as descriptive notions in the history of science

Irreversibility as a scientific concept originated in debates on thermodynamics and dissipative physical systems in the second half of the 19th century. In this context, irreversibility is closely linked to entropy (30), and used descriptively referring to a ubiquitous property of matter, processes, and systems (31). On the macroscopic level, irreversibility “is the natural state of affairs”, whereas complete reversibility, though an axiom of Newtonian mechanics, “is not actually attainable in the real world” (32). In the contexts of thermodynamic theory, mechanics, and engineering, reversibility and irreversibility are neutral scientific concepts that arise from their specific scientific paradigms.

### Irreversibility and reversibility as indicative and context-related notions in environmental research

In research on human-induced environmental change and ecosystem management, the concept of irreversibility is used in a different way. Here, in new applied research contexts, it has taken on several senses, functions and aspects, namely descriptive, evaluative-normative, and communicative-rhetorical. In the descriptive sense environmental or ecological irreversibility qualifies a transition, a shift or tipping, or more generally, a change after which it is impossible to return to a certain baseline. In this sense it is often used in conceptual frameworks as a qualifier for change or response behavior. The (ir)reversibility of a change can be relative to temporal and spatial scales, or it indicates terminal events or an absolute change where no return to a predisturbance state is possible, such as ecosystem collapse or extinction. In this way, the concept of irreversibility helps to refine threshold and transition concepts. In a normative sense, the classification of changes as irreversible indicates a (potential) transition to a qualitatively new state of an Earth system or an ecosystem under consideration. This state is generally considered to be undesirable or disvaluable, with negative impacts or harms to ecosystems, species, or humans (31, 33, 34). In this normatively informed sense and context of application, “irreversibility” indicates, as Folke et al. (7) aptly put it, “a reflection of changes in variables with long turnover times (e.g. biochemical, hydrological, or climatic) and loss of biological sources and interactions for renewal and reorganization [of ecosystems] into desired states”.

### Irreversibility as a term to emphasize urgency and risks to life and well-being

Sometimes the use of the normatively loaded irreversibility is underlined by a sense of urgency and a call for precaution to take appropriate action, for instance, by mitigating climate change rather than relying on the idea that humans could “reverse the global climate” by applying technologies for carbon dioxide removal, despite the lack of robust empirical evidence of such reversibility [996]. Especially when used in a nonsubstantive way, the collocations “irreversible changes” or “irreversible losses” can be understood as a warning term to communicate the high level of risk and detrimental consequences (34, 35); in other words, as a notion with a rhetorical function. Recognizing this rhetorical use is essential for critically applying it in the light of recent concerns about the effectiveness of fear-based narratives in creating behavior or policy change (36). In sum, the notion of irreversibility, as used in research on anthropogenic environmental change, appears to have become what philosophers call a “thick” concept, i.e. one that has descriptive and normative/evaluative dimensions (37). This implies that irreversibility in research on anthropogenic environmental changes (“anthropogenic environmental irreversibility”) is not evaluatively neutral (31), in contrast to thermodynamic irreversibility, even though these normative and communicative dimensions need not be addressed in every research article.

### Environmental, ecological, and thermodynamic irreversibility

It is noteworthy that irreversibility, as used in the context of research on anthropogenic environmental change, is not necessarily detached from the classical theory of irreversible thermodynamics. For instance, theoretical work on environmental and climate tipping points and studies on ecosystem stability in theoretical ecology refer to key concepts of thermodynamics such as entropy production, energy and steady states (9, 38). However, in the research contexts covered by our review, the notion of irreversibility is sometimes given aspects that are not always in line with thermodynamic irreversibility (see Fig. 3). As such, the conceptual understanding of (ir)reversibility and related notions such as “equilibrium” may differ depending on the theoretical or mathematical background of the scientists and the research field (39, 40). In the fields of geomorphology and geophysics, irreversibility, for instance, is used with respect to geomorphological evolution [201], material elasticity [612], and morphodynamics [844], and may relate differently to different types of equilibria (e.g. stable, unstable, metastable, dynamic, or graded) (39, 41).

### The ethics of temporal and spatial scales

As the temporality of irreversibility utilizes standards related to organic and human existence or geological timescales (years, decades, centuries, millennia), some of the risks or impacts identified raise concerns of global or intergenerational justice, as well as multispecies justice (42). For instance, research may emphasize that certain environmental changes are reversible on geological timescales, such as deglaciation. However, if these changes include irreversible changes and extinction on ecological timescales, such a claim would implicitly entail “declaring biodiversity dispensable” (19) and sacrificing existence and bequest values (43). Additionally, climate tipping points, ecosystem degradation and reversal interventions such as climate engineering have heterogeneous spatial impacts, involving reversibility and irreversibility at different spatial and temporal scales, for instance, regional climate reversibility (44). This, in turn, raises concerns about the appropriate definitions of “political” and “ethical” time horizons, as well as spatial horizons that are aligned with ecosystem change. Defining the horizon of relevance is not only a topic of debate in intergenerational ethics and political philosophy but should also be subject to democratic deliberation. The two papers offering definitions [21; 126] (see Table S3) can be seen as a contribution to this discussion on justice and democracy in light of fundamental environmental change and should be taken up by further interdisciplinary research. For any restoration or engineering intervention, the differentiation of multiple temporal, spatial, and systemic, i.e. equilibrium, scales of ecosystem resilience and recovery at stake implies a multiplicity of impacts on human and biotic groups and generations that need to be addressed by fine-grained facet- and scale-sensitivities included in the management frameworks, as has been recently shown in the fields of river ecosystem restoration (see, for instance, Polvi et al. (45), Fuller et al. (46); [402] and climate overshoot scenarios [337; 996] (44)).

### Reversibility research and decision-making

Environmental ethics pioneer Holmes Rolston III prominently formulated a “reversibility maxim”, which requires the avoidance of human activity that leads to irreversible change in the web of life: “[W]e should not disturb an ecosystem so that we cannot, if we later wish, put it back as it was” (47). Today, reversibility is claimed as “a criterion for project selection” in sustainable development (48); it is relevant for sustainable management of ecosystems and natural resources, ensuring their resilience and recoverability (49). Not all scientists tie the possibility of restoration and ecosystem recovery to reversibility. When recovery is tied to some kind or degree of reversibility, this, however, raises the crucial question of the possibility of reversibility of fundamental ecosystem changes and the need for differentiated assessment (50, 51): Only if the system has the ability to “return” or to “reverse” (technically: backshift), hence “revers-ability”, is recovery a live possibility. Against this backdrop it is not surprising that we see a shift towards “reversibility research” with an agenda to investigate empirical evidence of reversibility and its underlying mechanisms at multiple temporal and spatial scales (52) (see the conceptual focus on reversibility in the subject area of reversal intervention Fig. 1c). This applies to ecological as well as to large-scale changes such as those in the global climate. From this perspective, the use of (ir)reversibility reflects a shift in the way environmental and ecological research sees itself as contributing to the dissemination of knowledge for policy change, climate action and ecological restoration (53, 54): A significant number of articles conveying socially and politically normative statements and messages to the public, as revealed in our review, shows that many authors feel comfortable addressing their research findings and recommendations to policy-makers and the public (Fig. 1e). However, it is important to keep in mind that researchers and managers “may need to use the term ‘reversible’ differently”, the former considering longer timescales of centuries, the latter years and decades (50). Thus, there are different perspectives on reversibility and irreversibility and “long recovery” leading to different scientific and managerial or “practical” understandings.

### (Ir)reversibility as a decision of costs

In the translation of reversibility research into practice—environmental management, restoration, remediation, life cycle assessment, environmental impact assessment, and compensatory measures—the scientific classification of an ecosystem state or change as hysteretic or irreversible can strongly influence managerial and policy decision-making about (non)intervention, with either positive or negative implications for (policy) prioritization, the distribution of risks (35), and the option values remaining for future generations (34). As James Miller and Brandon Bestelmeyer (55) have pointed out in their reflection on the novel ecosystem concept, “[o]ftentimes, reversibility is a function of multiple factors, such as costs and public support”. In turn, “irreversibility” may imply the deliberate determination that effects are “too costly to reverse” (7) by involved actors (49). When revers-ability (i.e. the assisted or self-recovery of an ecosystem) is overemphasized, or when the overall repairability of deteriorated ecosystems is claimed to strategically justify action or inaction (11, 51), this may override scientific evidence and confidence of (ir)reversibility in decision-making (42). However, the potential or probable irreversibility of climate and global environmental change should not be misunderstood as a generally disempowering message for local and regional ecological restoration efforts, where at least partial reversibility of or recovery from damage and partial reversal of harm is a viable option (56). The terminology of (ir)reversibility is thus closely linked to societal ideas and values of recovery and repair, which feed political hopes and agendas and guide budget plans for intervention.

### Towards accurate and responsible usage of irreversibility/reversibility

Given the significant potentials for underdetermined use of irreversibility and reversibility that this review has identified, we recommend accuracy and consistency in future uses of these concepts in the context of anthropogenic environmental change and ecosystem management (see for an extended synthesis of recommendations, Box 2). First, scientific concepts can take unpredictable paths when translated into practice and decision-making, thereby confusing scientific frameworks and intuitive understanding, overriding empirical evidence, and affecting public views and values. For example, an analysis of climate engineering narratives found that framings emphasizing catastrophe were used to argue that implementing these technologies might be preferable when faced with irreversible climate regime changes (57). Given the very high stakes involved in environmental decision-making and policy, it is important for scientists to reflectively examine their use of the notions of irreversibility and reversibility. This appears especially important in light of our finding that 74.7% of the literature surveyed did not define or explain their use of (ir)reversibility, yet 27.5% of this literature contained normative statements warning against an outcome or signaling urgency. Second, similar to what has been observed with the notions of “tipping points”, the “Anthropocene” and the “Sixth Mass Extinction”, the concepts of irreversibility and reversibility may be used in completely different ways. Authors should therefore clarify their usage of the notions of (ir)reversibility by indicating whether they use them “precisely or strategically, scientifically or culturally, as a formal or informal category” (58), i.e. as a scientific concept or as a communicative tool. Thus, reflecting on the different uses and aspects of irreversibility and reversibility can help to ensure that these concepts remain epistemically valuable in various scientific contexts. Third, for irreversibility or reversibility to be used as scientific concepts in inter- and transdisciplinary collaboration, it is important to specify temporal and spatial scales as precisely as possible when applicable, which means considering a multiplicity of ecosystem scales (20, 25, 59). While some environmental changes are theoretically reversible on geological timescales, irreversible changes on ecological timescales—such as biodiversity loss and extinction—terminate life in the strict ontological sense of an end of existence, although so-called de-extinction technologies claim to be capable of overcoming even this (illustrating that irreversibility can be, at least theoretically, dependent on human motivation and socio-technical developments). Defining the “timescale of interest” and whose interest is taken into account signals the importance of issues of ecological, intergenerational and multispecies justice. Accurately and legitimately incorporating such issues may require stronger collaboration of scholars from the natural, environmental, social sciences and the humanities as well as of community stakeholders.

Box 2. **Recommendations for application of the concept of irreversibility and reversibility in the context of environmental and ecological research and when applied in the contexts of ecosystem management, ecological, economic and social sustainability, societal transformation, the possibility of recovery, restoration, and remediation, climate change mitigation and adaptation (that is the life in and with crossing/crossed climate tipping points), life cycle assessment, environmental impact assessment, assessment, and policies of ecological compensatory measures.**

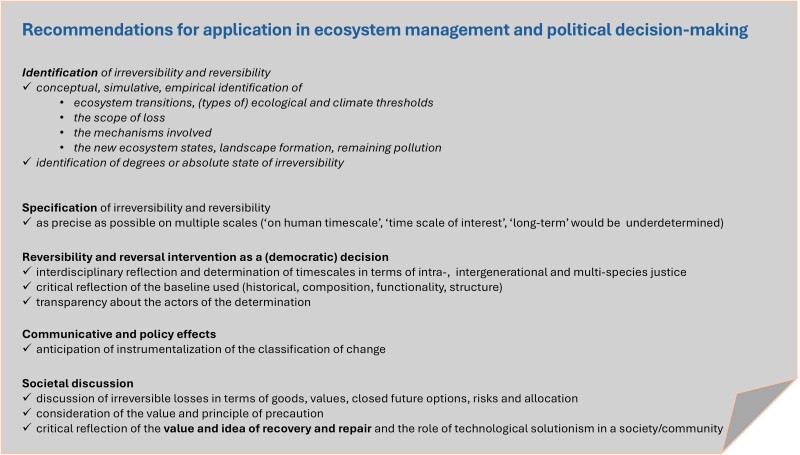

## Outlook

For future research, we recommend conducting narrative and systematic literature reviews of specific research areas and debates, such as landscape studies, sustainability studies, restoration sciences, and river sciences to elaborate their specific understandings and usage of the concepts of irreversibility and reversibility. Further reviews of the notions of irreversibility and reversibility in the study of anthropogenic change may include contributions from environmental ethics and environmental social science, as the notions are under-conceptualized in these fields as well (25, 34, 60–63). Future physics oriented environmental research on the concepts should focus on the clash between irreversibility and reversibility at different scales, also known as the “irreversibility paradox”, and include probability accounts of irreversibility. In general, we recommend that future empirical environmental and ecological research explores the reversibility and irreversibility of change using specified or more precise conceptual frameworks.

## Materials and methods

A literature review was conducted using the guidelines for systematic literature reviews, as originally introduced in medicine and the health sciences (64) and now extended for application across disciplines, as a methodological orientation and framework (65, 66). The aim was to trace the uses and functions of the concepts of irreversibility and reversibility in ecological and environmental research on anthropogenic environmental change. We addressed the following research questions: (i) How is the concept of irreversibility and related terminology used and determined? (ii) What are subject areas, thematic foci and conceptual linkages in scientific articles that use the concepts of “irreversibility” or “reversibility”? (iii) What are implicit and explicit normative aspects of the usages of the concept of irreversibility? The study had been preregistered with a protocol with the Open Science Framework (https://doi.org/10.17605/OSF.IO/QE9XC) before the search started. Comprehensive database research was carried out twice, on 2023 January 20 and on 2023 September 23, using the databases Web of Science (Science Citation Index Expanded + Emerging Source Citation Index), Scopus, and GreenFILE, and deploying a mixed search principle (sensible and specific). Except for the language filter (English), no other filters were set; see Fig. S3 for the research strings). Additionally, a screening of the reference lists and the literature cited in the included articles identified a few more relevant articles (*n* = 11). The reporting followed the reporting guidelines of the PRISMA-2020 extension statement and its checklist (67) and the PRISMA 2020-EcoEvo checklist (Fig. S3, Table S6). In addition, the “refhunter” protocol (68) (German template, partly translated into English) was used to protocol the construction of the research string (available on request) in order to document decisions on the construction of the research string and to ensure replicability.

### Screening

We carried out three steps of screening. First, titles, abstracts and keywords of the deduplicated database records (*n* = 14,705) were screened using the web-based tool *colandr*, which uses machine learning and natural language processing. *Colandr* helped to sort target articles during the screening process. In total, we screened a third of the articles manually until a saturation point was reached in *colandr* (*n* = 4,905). The second step was a full-text screening of included articles (*n* = 996). Third, three rounds of critical appraisal of included articles were carried out, one after each screening step and a third during the in-depth analysis. A pilot study was conducted to test the eligibility criteria and the reliability of the reviewers; additionally, the eligibility criteria were refined during the first stage of screening. All records from the database search were screened by two reviewers independently and discussed in case of disagreement; for borderline cases (*n* = 11) a decision committee was set up to decide on inclusion by consent.

### Eligibility

We were interested in scientific articles, including editorials, opinions, and perspectives, on anthropogenic environmental changes from the viewpoint of natural sciences, including environmental, geo, Earth system sciences, and ecology published in scientific journals or edited volumes. Literature and methodological approaches that focus primarily on human well-being, such as ecosystem management and agriculture in terms of ecosystem services or natural resources used for economic productivity and development, were excluded (see Table S4). Similarly, articles from the target disciplines that do not deal with anthropogenic change but with general theoretical and modeling accounts (such as irreversibility in theoretical ecology and general evolutionary theory) were also excluded. We distinguished three levels of eligibility for the second screening stage: (i) inclusion for in-depth analysis due to substantive use of the notions of irreversibility or reversibility, i.e. the group of prioritized articles, (ii) exclusion from in-depth analysis, but inclusion to brief analysis due to nonsubstantive use of the inquired notions, i.e. the group of nonprioritized articles, and (iii) entire exclusion from analysis due to no use of the terms of (ir)reversibility at all, wrong research field, or wrong topic, duplication, or formal failure (see Box 1). Substantive use, as we understand it, is evident when the terms play a key role in the overall argument of the paper, in the rationale for the research, or in describing the results. Nonsubstantive use of terms means they are used a few times and are not key to the article. A significant proportion of the papers that underwent the first stage did not use of the terms of (ir)reversibility at all at the second stage of screening (full-text screening) (26.8%, 267/996). They were initially included because the title, abstract and keywords appeared to have some relevance to the research questions and at least used the term “tipping”.

### Analysis

We conducted a qualitative content analysis, supplemented by simple quantitative and bibliometric analysis of the articles in which we identified a substantive use of the notions of irreversibility or reversibility. For the qualitative analysis, we used mixed coding with mainly deductive categories and codes, supplemented by some inductive codes (69) (Table S5). The codebook was progressively refined. The coding and categorization of normative statements was agreed by a subgroup of three researchers and widely discussed by the whole research group. After coding the relevant data were charted according to the coding categories. Half of the articles that were coded and charted were analyzed by two reviewers and the remaining were analyzed by the principal investigator. For bibliometric analysis, we used the web-based research platform ResearchRabbit (as provided in April 2024), VOSViewer 1.6.20, and the web application Local Citation Network (as provided in April 2024). The articles with nonsubstantive uses of irreversibility and reversibility appeared also worth consideration. We therefore carried out a brief content analysis, using a reduced codebook of deductive categories (see Table S5b). Brief content analysis, including coding and charting, was carried out by the principal investigator. While for the in-depth analysis of the articles with substantive use of the target notions we did a close reading of the entire articles, for the short analysis only the close textual contexts in which the target notions were used (i.e. the sentences, the section, or the paragraph) were considered for analysis. Here we were mainly interested in whether the notions were used to indicate serious consequences of the environmental or ecological changes under consideration, i.e. to warn of bad effects or to emphasize the urgency of taking countermeasures, and if any specification or determination of the notions were given.

### Limitations

The findings in this review report are subject to multiple limitations. First, we decided not to use synonyms for irreversibility, such as irretrievable or irreplaceable, or synonyms for reversibility, such as recoverability. The narrow search strategy to focus on irreversib* and reversib* may have led to the omission of some articles that are closely related to the target research areas on anthropogenic environmental and ecological change. However, since we were only interested in the notion of irreversibility (and reversibility) itself, we believe that this narrow scope was justified. In the screening and analysis, we slightly expanded the linguistic scope to also include terms around revers*, that is, reversal or reversing, when used in the sense of ecosystem reversibility. Second, to keep the review feasible within the given time and cost constraints, we decided not to consider monographs and edited volumes for inclusion in addition to database research. The focus on searching scientific database only, supplemented by selective inclusion through reference list screening as a standard in the review methodology, can be considered a weakness, since relevant works—and entire journals or older issues of journals—that are not included in the databases consulted do not appear at all, e.g. not all articles published in edited volumes or articles that were produced in print versions are included in databases. In this sense, the methodological approach is implicitly biased towards recent and contemporary literature. Third, some articles, although relevant to the discussion on the use of (ir)reversibility, may have been excluded in the first screening step because they showed no evidence of substantive use of the target concepts in the title, abstract, and keywords. We were aware that there were such cases in the literature on climate and environmental tipping points, so we generously included papers with “tipping” terminology in the full-text screening. However, we did not have a general backstop for similar cases across all subject areas. Future systematic reviews may benefit from AI tools that screen the full-text before making exclusion decisions. Fourth, it should be recalled that the purpose of this review was to cover a broad range of research, but we excluded work based on social–ecological frameworks. Hence, not all scientific debates and contexts that deal with the issue of (ir)reversibility, are included in detail—either for methodical or technical reasons related to the database research or for reasons of consistency in eligibility criteria. For instance, multi- and transdisciplinary research fields, programs and sciences, such as river science (70) and restoration science based on a social–ecological framework or adaptive cycles approach, landscape studies, and sustainability studies, as well as the discussion about the concept of novel ecosystems (53, 71, 72) are touched upon but do not form the main body and target of this review. Thus, we understand this review as a rather broad, by no means exhaustive contribution to the reflection on (ir)reversibility terminology across disciplines and perspectives.

## Supplementary Material

pgae577_Supplementary_Data
