# Correction to: The concepts of irreversibility and reversibility in research on anthropogenic environmental changes

**DOI:** 10.1093/pnasnexus/pgaf036

**Published:** 2025-02-22

This is a correction to: Lorina Buhr, Dominic S Lenzi, Auke J K Pols, Claudia E Brunner, Andrea Fischer, Arie Staal, Benjamin P Hofbauer, Bernice Bovenkerk, The concepts of irreversibility and reversibility in research on anthropogenic environmental changes, *PNAS Nexus*, Volume 4, Issue 1, January 2025, pgae577, https://doi.org/10.1093/pnasnexus/pgae577

In the originally published version of this paper, a production error caused the captions for boxes 1 and 2 to be reversed.

The correct caption for Box 1 is “Decision scheme that was applied for the selection of articles.”

The correct caption for Box 2 is “Recommendations for application of the concept of irreversibility and reversibility in the context of environmental and ecological research and when applied in the contexts of ecosystem management, ecological, economic and social sustainability, societal transformation, the possibility of recovery, restoration, and remediation, climate change mitigation and adaptation (that is the life in and with crossing/crossed climate tipping points), life cycle assessment, environmental impact assessment, assessment, and policies of ecological compensatory measures.”

This error, for which the publisher apologizes, has been corrected.

