# Supplementary material for: The concepts of irreversibility and reversibility in research on anthropogenic environmental changes
Source: PNAS Nexus. 2024 Dec 31;4(1):pgae577. doi: 10.1093/pnasnexus/pgae577 (PMC11740731; doi:10.1093/pnasnexus/pgae577)
Supplement: pgae577_Supplementary_Data [file pgae577_supplementary_data.zip › PNASNEXUS-PNASNEXUS-2024-00908RR-s01.pdf]

Buhr et al. (2024): The concepts of irreversibility and reversibility in research on anthropogenic environmental changes

Supplementary Material 1

Contents

Results

Table S1 Substantive terminological usages across subject areas and topics

Table S2 Definitions and conceptual innovations (extended list)

Table S3 Selection of temporal, spatial and further specification of irreversibility

Figure S1 Bibliometric analysis: author and citation analysis (in-depth analysis/substantive use)

Figure S2 Illustrations of (ir)reversibility (extended analysis/substantiveuse)

References Supplementary Material Results

Methods

Figure S3 PRISMA flow diagram

Table S4 Eligibility Criteria

Table S5 Qualitative content analysis categories of coding and data extraction

Table S6 PRIMA 2020 checklist *[preliminary]*

Reference Lists (RL)

Reference List S1 Articles with substantive uses of reversibility/reversible, irreversibility/irreversible (in-depth analysis)

Reference List S2 Articles with non-substantive use of reversibility/reversible, irreversibility/irreversible (brief analysis)

Other supplementary information:

Supplementary Material 2: Protocol Search String

Supplementary Data

Dataset S1: Articles with substantive use (n=91)

Dataset S2: Articles with non-substantive use (n=319)

Supplementary Material: Results

Contents

Table S1 Substantive terminological usages across subject areas and topics 2

Table S2 Definitions and conceptual innovations (extended list) 5

Table S3 Selection of temporal, spatial and further specification of irreversibility 8

Figure S1 Bibliometric analysis: author and citation analysis (in-depth analysis/substantive use) 9

Figure S2 Illustrations of (ir)reversibility (extended analysis/substantiveuse) 12

References Supplementary Material Results 13

Table S1 Substantive terminological usages across subject areas and topics

Table S1 Substantive terminological usages across subject areas and topics (extended version) (n= 91). We differentiated between three conceptual foci: *irreversible/irreversibility used as one or as *the* key concept or category; **reversible/reversibility used as one or as *the* key concept or category; ***irreversible/irreversibility *and* reversible/reversibility are used together as *one or the* key category for research. Some articles covered more than one topic (up to 3 topics per article found, n=26), we counted each article once per topic. b) Conceptual functions. Some articles covered more than one conceptual function (up to 4 functions/usages per article found, n=78), and/or more than one conceptual focus (up to 3 foci per article found, n=4). We counted each article once per function, regardless of the number of topics covered by the function.

	**Conceptual functions of irreversible/irreversibility*, or reversible/reversibility**, or ir/reversible/ ir/reversibility***** The concepts are used for/as…								
**Subject areas and topics**	conceptual innovation	definiendum	definiens	definitional extension	one or the key analytical criterion or research category	one or the key notion in the description or classification of research findings			
				potentiality, quantification, explication	object of investigation, rationale	empirical (field study; monitoring; experiment)	modelling/ simulation (incl. conceptual models)	review, meta-analysis, assessment	%
**(A) Systemic transition**									**66.3**
1 Ecological thresholds/regimes shifts/tipping points									28.2
investigation of thresholds (including resilience)									11.7
marine, aquatic ecosystems				[237***]	[237***]		[237***]	[237***]	0.9
eutrophication, acidification, salination, pollution					[367*] [564*]	[238*]	[564*]	[43*]	3.6
aquatic animals							[398**]		0.9
bacterial response					[569*]	[569*] [764*]			1.8
forest response					[501**]	[320*] [973***]	[35***]		3.6
investigation of transitions									6.3
forest to-grassy ecosystem					[565***]	[565***]	[35***]		1.8
savanna to woody vegetation						[819*]		[59***]	1.8
grassland to shrub- or woodland		[520*]			[520*] [992**] [924**]	[520*]			2.7
investigation of ecological or ecosystem degradation									10.1
meadows, rangeland		[620*]	[770***]	[620***],	[451**] [620***] [770***] [834*]	[451**] [620***] [770***] [896***] [994**]			5.5
soil functioning/erosion/pollution	[387*]	[107**] [298*] [387*]	[54***]		[54***] [298*]	[387*]	[107***]	[298*] [995***]	4.6
2 Earth system tipping points/elements/threshold behaviour									19.9
cryosphere									7.2
Arctic sea ice				[122***]			[262***]	[122***]	1.8
Greenland Ice Sheet	[211*] [754*]	[211*]					[211***] [754*]		1.8
West Antarctic Ice Sheet	[870*]	[938**]			[938**]		[870*] [938**]		1.8
across the cryosphere					[506**]		[506*]	[176***]	1.8
hydrosphere									4.5
Atlantic Thermohaline/Occean Circulation		[234**]					[234*] [355***]		1.8
ocean acidification, deoxygenation								[73***]	0.9
sea level rise					[733**] [993**]		[733**] [993**]		1.8
across Earth systems									8.2
	[56*] [377*]	IPCC 5* IPCC 6* [119*,**] [377*] [983*,**]		IPCC 5* IPCC 6* [21***] [119***] [126***]	[250*] [119***] [983***] [989**]		[989**]	IPCC 5* IPCC 6* [56*] [377***] [983***] [149***]	8.2

3 General approaches to environmental changes: theory and concept building									18.2
general tipping theory, conceptual models			[184*]<br>[335*]	[124*]<br>[180***]<br>[335***]	[807**]<br>[834*]<br>[875*]			[124*]	6.4
typologies of ecological change		[509**]<br>[995*]			[226***]<br>[509**]<br>[520*]<br>[564*]<br>[995*]		[509**]	[43*]	6.4
modelling of ecosystem response or degradation	[367*]	[367*]<br>[332*]			[367*]<br>[620***]<br>[332*]				2.7
climate modelling					[389*]				0.9
physics of climate change; entropy				[55*]					0.9
Anthropocene concept								[59***]	0.9
**(B) Investigation of impacts**									**29.0**
1 Impacts of greenhouse gas emissions									13.6
on the cryosphere		[212**]			[714*]		[212***]<br>[714*]		1.8
on the biosphere	[511*]				[511*]	[511*]			0.9
on climate change/surface temperature/ precipitation	[967*]	[102*,**]<br>[286*]<br>[527*]<br>[967***]			[967***]<br>[988**]<br>[990**]		[102***]<br>[243*]<br>[402***]<br>[527***]<br>[596***]<br>[967***]<br>[988**]<br>[990**]		7.3
on the hydrosphere							[337***]		0.9
ocean warming, thermal state					[337***]		[337***]<br>[733***]		1.8
ocean acidification							[402***]		0.9
2 Impacts as consequences of climate change									6.3
glacial retreat on rock slopes, sea level rise				[612***]		[612***]	[776*]		1.8
droughts/heat waves/ global warming on vegetation/vegetation response		[167**]<br>[491*]		[491*]	[167***]<br>[891***]<br>[903*]	[903*]<br>[987***]		[891***]	3.6
global warming on bedrock permafrost	[286*]				[286***]	[286*]			0.9
3 Direct impacts of human activity									9.1
on rivers, estuaries, sea			[799*]<br>[844*]		[201*]<br>[300***]<br>[799***]	[238*]<br>[799***]<br>[844***]	[201*]		4.6
livestock grazing on rangeland					[620***]<br>[875*]	[620***]<br>[819*]<br>[994**]			3.6
land use on biodiversity					[634***]				0.9
**(C) Evolution and extinction**									**4.5**
population dynamics						[738*]			0.9
levels of species loss, extinction			[634***]						0.9
evolutionary response	[526*]			[526***]		[764*]	[526***]		1.8
invasive/alien species					[840***]			[840***]	0.9
**(D) Scenarios of reversal intervention**									**16.3**
1 Biological conservation									0.9
animals and amphibians					[472***]			[472***]	0.9
2 Ecosystem restoration, recovery									5.4
grazing reversal						[896***]<br>[992**]<br>[994**]			2.7
aquatic ecosystem recovery					[145***]			[145***]	0.9
soil restoration	[387*]	[387*]				[387***]			0.9
across ecosystems					[226***]				0.9
3 Climate engineering									10.0
CO_2 removal, sequestration, cut of emissions	[967***]	[102*, **]<br>[967***]<br>[996**]		[147*]	[337***]<br>[967***]<br>[988**]<br>[989**]<br>[990**]<br>[996***]		[102***]<br>[527***]<br>[733***]<br>[967***]<br>[988**]<br>[989**]		9.1

							[990**] [996***]		
Solar-radiation management					[124***]			[124***]	0.9
	12.1	25.3	7.7	14.3	[illegible]4.9	27.5	34.1	18.7	

Table S2 Definitions and conceptual innovations (extended list)

Table S2 Definitions and conceptual innovations (extended list). a) Definitions of irreversibility and reversibility, b) Conceptual innovations using irreversibility/irreversible or reversibility/reversible

a) Definitions of irreversibility or reversibility

	pdf no.	Reference	Topic	Definition
	IPCC AR5		glossary	"A perturbed state of a dynamical system is defined as **irreversible** on a given timescale, if the recovery timescale from this state due to natural processes is substantially longer than the time it takes for the system to reach this perturbed state. In the context of this report, the time scale of interest is centennial to millennial. See also Tipping point."
	IPCC AR6		glossary	"A perturbed state of a dynamical system is defined as **irreversible** on a given time scale if the recovery from this state due to natural processes takes substantially longer than the time scale of interest. See also: Tipping point."
1	102	Kim et al. (2022)	*CO_2 removal*	"The ability of the climate system to be restored to its initial state is referred to as **reversibility**." (p. 834) "**Reversibility** of a system can be measured as whether the trajectory return to its initial state, indicated as an open loop (irreversible change) and closed-loop (reversible change) […]." (p. 835) "an open-loop trajectory does not always indicate that a system is completely irreversible. Even if the loop is open, there is a possibility that the system will returns to its initial state if sufficient time is provided after the forcing reaches the initial level. Nevertheless, at least, they show that the climate system cannot be immediately restored to its initial state even after successful removal of the atmospheric CO2. The **soft definition of irreversibility** provides a practical classification for climate recoverability within a human-perceptible timescale." (p. 835)
2	107	Kramer & Mau (2020)	*Degradation of soil functioning*	"Existing models for the effect of salinity and sodicity on K_s *[hydraulic conductivity]*, including the Ezlit et al. (2013) model, consider soil degradation and rehabilitation to be **reversible processes**. That is, while changes to soil salinity and sodicity can cause *Ks* to decline, if the changes to salinity and sodicity are reversed, then *Ks* will immediately return to its original value. The scant experimental evidence that exists, however, suggests that these processes feature hysteresis, that is, the system follows different paths for degradation and rehabilitation (Dane & Klute, 1977)." (p. 10)
3	119	Lenton (2014)	*Climate tipping points and elements*	"Tipping point change also includes transitions that are slower than their cause (in both cases the rate is determined by the system itself). In either case the **change** in state may be reversible or irreversible. **Reversible** means that when the forcing is returned below the tipping point the system recovers its original state (either abruptly or gradually). **Irreversible** means that it does not (it takes a larger change in forcing to recover). Reversibility in principle does not mean that changes will be reversible in practice." (p. 25-6)
4	167	Munson (2021)	*Impacts of climate change on plants*	**"Reversible**: Previous level of plant or ecosystem performance is restored following the return of favorable hydrologic condition." (p. 741)
5	211	Ridley et al. (2009)	*Melt of the Greenland Ice Sheet*	"The original ice-sheet volume can be regained only if the volume has not fallen below a threshold of **irreversibility**", which lies between 80 and 90% of the original value." (p. 1049)
6	212	Ridley & Hewitt (2014)	*Impact of GHG on the cryosphere*	"We thus define **reversibility** as the existence of a unique state (allowing for short time scale internal variability defined by 2 standard deviations from the trend, using the prediction method) of the system for a specific global temperature." (p. 8406)
7	234	Schleussner et al. (2015)	*Atlantic Occean Circulation*	"[W]e adopt a statistical notion of time series **reversibility**: A stationary stochastic process or time series {xi} is called reversible if for arbitrary *m*, the tuples (x_n, x_n+1, . . . , x_{n+m}) and (x_{n+m}, x_n+m-1, . . . , x_n) possess the same joint probability distribution (Lawrence 1991) […]. It is important to note that this definition of time series reversibility is distinct from more commonly known thermodynamic notions of the time reversibility of physical processes that derive from the second law of thermodynamics." (p. 3625)
8	243	Solomon et al. (2009)	*Climate Change*	"Future carbon dioxide emissions in the 21st century will hence lead to adverse climate changes on both short and long time scales that would be essentially irreversible (where **irreversible** is defined here as a time scale exceeding the end of the millennium in year 3000 […])." (p. 1704)
9	298	Yaron et al. (2010)	*Soil pollution*	"By **irreversible changes**—on a human time scale—we refer to long-term, stable, and persistent transformations of subsurface structure and properties, which are also resistant to remediation procedures and to natural attenuation." (p. 2)
10	332	Alkhayuon et al. (2021)	*Ecosystem responses*	"By '**irreversible**' we mean that the system approaches a different state in the long term." (p. 2)
11	335	Lenton (2013)	*Tipping theory*	"Catastrophic bifurcations and some phase transitions involve **irreversible changes** [...], which means that when the control parameter is returned to the threshold value (.crit) it does not revert to its original state. However, other noncatastrophic bifurcations and phase transitions are reversible at the same value of the control". (p. 6) "The resulting change may ultimately be reversible (i.e., the system eventually returns to the original attractor)." (p. 7)

12	367	Wu, Liang & Liu (2019)	*Eutrophication*	"[…] we proposed a probabilistic indicator, RID, to quantify the risk of **irreversible degradation**. It is defined as the probability of the threshold for recovery (T2) being unattainable under real world scenarios." (p.2)
13	377	Swingedouw D et al. (2020)	*Tipping elements*	"[T]his type of bifurcation can lead to irreversible change (where irreversible means that the recovery time scale from this state is substantially longer than the time it takes for the system to reach this perturbed state, cf. Masson-Delmotte et al. 2018)." (p. 1238)
14	387	Gao et al. (2011)	*Soil de-gradation and restoration*	"A major problem was identified, which we refer to as the '**irreversible** loss of soil services'; when vegetation cover decreases below a degradation threshold, this leads to sustained degeneration of the vegetation community, erosion of the surface soil and declining soil fertility. These changes represent a severe and long-lasting disturbance that will prevent ecosystem recovery in the absence of comprehensive artificial restoration measures." (p. 1145)
15	491	Vedhuyshkin (1995)	*Impacts of GHG emission on climate change*	"When a critical level of environmental change occurs, the boundary begins to move and the vegetation type changes irreversibly at the place under consideration. **Irreversibility** means that if the environmental change is reversed, the boundary will not move until the reversed environmental change reaches a value lower than at the beginning of the process." (p. 2)
16	509	Bagchi (2017)	*Typology of ecological change*	**"Reversible** change: major community changes that later return to a former configuration, reflected as initial increases in compositional dissimilarity that are followed by increasing similarity with initial composition." (p. 1515)
17	520	Bielski et al. (2021)	*Grassland to woodland transition*	"The implication that a regime shift from grassland to juniper woodland is **irreversible** means that costly post-fire interventions, such as mechanical removal, are requisite for a return to the grassland regime (Twidwell et al., 2013a)."
18	527	Boucher et al. (2012)	*Climate modelling*	"**Irreversibility** means that the system cannot be restored to its initial state or only does so on a timescale far longer than those normally considered practical from a human perspective." (p. 2)
19	620	Jamsranjav et al. (2018)	*Rangeland degradation*	"Here, we define degradation as a sequential process of progressive departure from a reference ecological state for a given ecological site (climate, landform, and soil complex), accounting for natural fluctuations in reference conditions due to precipitation and native herbivores. In this conceptualization of degradation, different levels of departure from reference conditions exist, some of which are reversible, and others of which denote permanent and irreversible changes in ecological structure and/or function. Once a rangeland is **degraded irreversibly**, its pre-degradation state may not be attained even with significant management efforts, due to changes in soil physical properties and the resulting feedbacks to plant communities (Bestelmeyer et al. 2013)." (p. 623)
20	938	Caillet et al. (2022)	*West Antarctic Ice Sheet*	"The transitions are **reversible**, that is, cancelling the atmospheric perturbation brings the ocean system back to its unperturbed state within a few decades." (p. 14)
21	967	Mondal et al. (2023)	*CO_2 removal*	"[**R**]**eversibility** is the ability of the Earth's climate system to be restored to its original state; when it returns to its initial condition known as reversible change, and the opposite case reflect irreversible change (An et al., 2021a,b; Yeh et al., 2021; Kug et al., 2022). **Irreversibility** of a climate structure can transpire when a climatic variable undergoes a threshold specifically as a 'tipping point' or when the response spans of that variable is comparatively more extensive than that of the forcing timescale (An et al., 2021a,b; Armstrong McKay et al., 2022; Kim et al., 2022). Notably, such irreversibility of a climate system typically consorts the hysteresis effect." (p. 2)
22	983	Wang et al. (2023)	*Tipping elements of the Earth system*	"[W]e use the term **'irreversible'** to refer only to changes that cannot be halted or returned to the original state on timescales of less than hundreds of years. In contrast, a '**reversible'** process can undergo more rapid reversion to the original system state in under a century under the right conditions. Note that the terms "tipping elements" and "non-tipping elements" in and of themselves do not imply whether changes are reversible or irreversible." (p. 3)
23	995	Gordon et al. (2008)	*Regime shifts in soil ecosystems*	"**Irreversible change** (Figure Id) is a stronger form of hysteresis, where vegetation is unable to recover to its pre-collapse levels, even if rainfall increases substantially. This type of dynamic can occur when the ability of vegetation to recover is lost from the system during a collapse." (p. 213)
24	996	Schwinger et al. 2022	*Climate system, CO_2 removal*	"We define '**reversibility'** based on a reference pathway without overshoot (i.e. no CDR applied) and based on cumulative carbon emissions (i.e. the overshoot simulations have the same amount of cumulative carbon emissions after CDR than the reference pathway)." (p. 1641) "We define an aspect of the Earth system to be **reversible** through the application of CDR if the mean state after an overshoot is within the internal variability of the reference case without overshoot. We stress that this definition neither implies reversibility in the absence of CDR nor reversibility of climate change that is committed to in the reference scenario." (p. 1656)

b) Conceptual innovations using irreversibility/irreversible or reversibility/reversible

	pdf no.		Topic	Conceptual Innovations
1	56	Good et al. (2018)	*Climate thresholds*	Key finding: "Some **degree of irreversible loss** may have begun, although the eventual magnitude and rate of this irreversible loss is uncertain." (p. 27)
2	211	Ridley J et al. (2009)	*Melt of the Greenland Ice Sheet*	"Our aim is to look for a **threshold of irreversibility** in the size of the ice-sheet, above which it could regrow in pre-industrial CO2, and below which it would be inevitably lost. [...] This threshold is of practical significance, because it is a 'point of no return', which would set a constraint on acceptable future timelines of CO2 concentration (Lowe et al. 2008) in order to avoid the eventual loss of the ice sheet, which is an outcome that might well be regarded as a dangerous consequence of climate change." (p. 1050-1)
3	286	Weber et al. (2017)	*Mountain cryosphere*	"We build a metric (termed **irreversibility index**) that aims at detecting periods during which overall kinematics is not dominated by thermoelastic strains. This index uses the absolute difference (1y) between the observed fracture data (yobs) and the modeled reversible fracture kinematics component (yrev) given by the LRM as input [...]". (p. 572)
4	367	Wu S, Liang Z & Liu Y (2019)	*Eutrophication*	"To verify our hypothesis and quantitatively measure this risk, we proposed a probabilistic indicator, **RID**, to quantify the **risk of irreversible degradation**. It is defined as the probability of the threshold for recovery (T2) being unattainable under real world scenarios." (p.2)
5	377	Swingedouw D et al. (2020)	*Tipping points across Earth systems*	**"Irreversibility potential** of change**"** (used as a categorial qualifier for assessed tipping elements, e.g., the potential is valid, probable, possible, unlikely, varying)
6	387	Gao Y et al. (2011)	*Soil de-gradation and restoration*	"A major problem was identified, which we refer to as the '**irreversible loss of soil services'**; when vegetation cover decreases below a degradation threshold, this leads to sustained degeneration of the vegetation community, erosion of the surface soil and declining soil fertility. These changes represent a severe and long-lasting disturbance that will prevent ecosystem recovery in the absence of comprehensive artificial restoration measures." (p. 1145)
7	511	Bartsev SI, Degermendzhi AG & Erokhin DV (2008)	*Impacts of GHG emission on the biosphere*	"Different curves correspond to different dates when fuel combustion is stopped, thus, making a digression from Scenario B2. This unrealistic element is introduced in order to demonstrate that even if fuel combustion is stopped completely, there still will be an '**irreversibility date'**, after which the catastrophic process in the system becomes irreversible. The test on the sensitivity of the model to T_{del} variations in the range 1.5–6°C shows the stability of the irreversibility phenomenon: as the greenhouse effect is reduced, the "irreversibility date" becomes more remote. It is also important that prior to the "irreversibility date" and for some time after it, biosphere and climate parameters change in a gradual, unalarming manner. By varying combinations of different values we obtained very pessimistic scenarios, when the date of irreversible changes in the biosphere is around 2035." (p. 164)
8	526	Botero et al. (2015)	*Evolutionary response to climate change*	"We label this strategy **irreversible plasticity** because individuals in these populations exhibit plasticity exclusively during development. The transition from reversible to irreversible plasticity occurs at progressively shorter timescales in less predictable environments because the expected benefits of phenotypic adjustment decrease with higher potential for errors in anticipating environmental change." (p. 185)
9	754	Gregory JM, George SE & Smith RS (2020)	*Modelling of Greenland Ice Mass Loss*	"This result suggests that, for slow or quasi-static decline of the ice sheet, the no-north mass itself is the **threshold of irreversibility**." (p. 4312)
10	870	Rosier et al. (2021)	*Modelling West Antarctic Ice Sheet*	"Hereafter we will refer to the former as **irreversible**, in line with previous studies, and the latter as **permanently irreversible**, to differentiate the two. Diagnosing whether a tipping point has been crossed without some prior knowledge of the system is not generally possible with outreversing the forcing to see if hysteresis has occurred." (p. 1502)
11	967	Mondal et al. (2023)	*CO_2 removal*	"The exposed population is prominent in South Africa and Asia. Notably, the population change effect is the principal factor in global exposure change, while it is the climate change effect over the **hotspots of irreversibility**."

Table S3 Selection of temporal, spatial and further specification of irreversibility

Table S3 Selection of temporal, spatial and further specification of irreversibility as found in the articles with substantive terminological use.

Specification	Definition	Exemplary articles
Temporal		
‘human timescale’		
ethical time horizon (T_E)	(T_E)= ~1,000 years<br>(T_E)= ~10,000 years	[119], [126]<br>[21]
political time horizon (T_P)	T_P = <100 years, critical time within this millennium = by 2100	[21], [126]
decades to millennia		[337], [377], [402]
millennia		[501], [754]
multiple/varying (without further definition)		[3], [149]
permanent/persistent; “short-term”, “long-term”		[332], [992]
Spatial		
global		[983], [996]
‘at least subcontinental in scale (of the order of 1000 km, i.e., ~1 M km2)’		[21]
regional		[211]
local		[201], [237], [994]
Further specification		
impacts on human population	> 100m	[21]
by stochastic processes in relation to time		[367], [226]

Figure S1 Bibliometric analysis: author and citation analysis (in-depth analysis/substantive use)

Figure S1 Bibliometric analysis: author and citation analysis (in-depth analysis/substantive use). We performed bibliometric analysis using VOSviewer version 1.6.20 and the browser-based tools Researchrabbit, and LocalCitationNetwork. As reference metadata may be incomplete, 100% completeness is not granted for online tools. **a)** author connections as provided by Researchrabbit (95% completeness), **b)** co-authorship network as provided by LocalCitationNetwork (91% estimated completeness, OpenAlex API, minimum input articles per author: 2), **c)** citation network of the articles as provided by LocalCitationNetwork (91% estimated completeness), **d)** top 10 references cited by the articles as provided by LocalCitationNetwork (91% completeness), **e)** top 10 of the most cited articles within the group the articles included for in-depth analysis as provided by LocalCitationNetwork (91% completeness), **f)** bibliometric analysis of the most relevant keywords provided by VOSviewer 1.6.20, methods used: full counting, association strength, minimum items: 2; 32 items and 6 cluster were included in the graph, 83 total link strength, scale 1.04., overlay visualization**.**

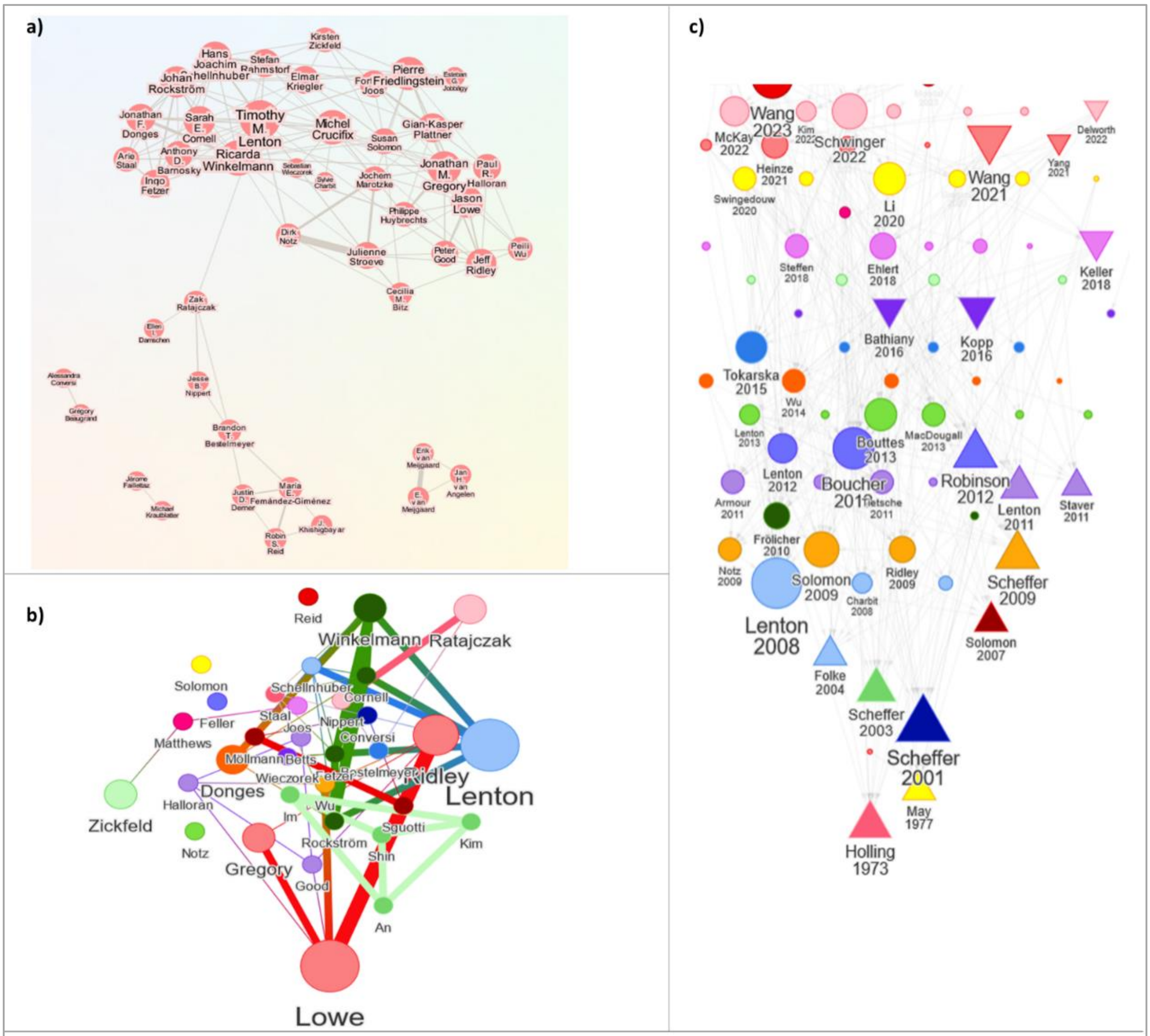

d)

Title	Author	Year	Cit	InDeg
Catastrophic shifts in ecosystems	Scheffer	2001	5540	14
Early-warning signals for critical transitions	Scheffer	2009	3410	11
Resilience and Stability of Ecological Systems	Holling	1973	13225	11
Catastrophic regime shifts in ecosystems: linking theory to observation	Scheffer	2003	2374	10
Multistability and critical thresholds of the Greenland ice sheet	Robinson	2012	223	9
Early warning of climate tipping points	Lenton	2011	652	9
Climate change 2007 : the physical science basis : contribution of Working Group I to the Fourth Assessment Report of the Intergovernmental Panel on Climate Change	Solomon	2007	11782	8
Regime Shifts, Resilience, and Biodiversity in Ecosystem Management	Folke	2004	3111	8
Thresholds and breakpoints in ecosystems with a multiplicity of stable states	May	1977	1475	8
The Global Extent and Determinants of Savanna and Forest as Alternative Biome States	Staver	2011	1091	7

e)

Title	Author	Year	Cit	InDeg
Tipping elements in the Earth's climate system	Lenton	2008	2848	15
Reversibility in an Earth System model in response to CO ₂ concentration changes	Boucher	2012	105	9
Irreversible climate change due to carbon dioxide emissions	Solomon	2009	2503	9
Reversible and irreversible impacts of greenhouse gas emissions in multi-century projections with the NCAR global coupled carbon cycle-climate model	Frölicher	2010	102	6
Reversing climate warming by artificial atmospheric carbon-dioxide removal: Can a Holocene-like climate be restored?	MacDougall	2013	27	6
The Reversibility of Sea Level Rise	Bouttes	2013	52	5
Thresholds for irreversible decline of the Greenland ice sheet	Ridley	2009	107	5
Trajectories of the Earth System in the Anthropocene	Steffen	2018	1982	5
Recovery mechanisms of Arctic summer sea ice	Tietsche	2011	189	5
The future of ice sheets and sea ice: Between reversible retreat and unstoppable loss	Notz	2009	143	5

f)

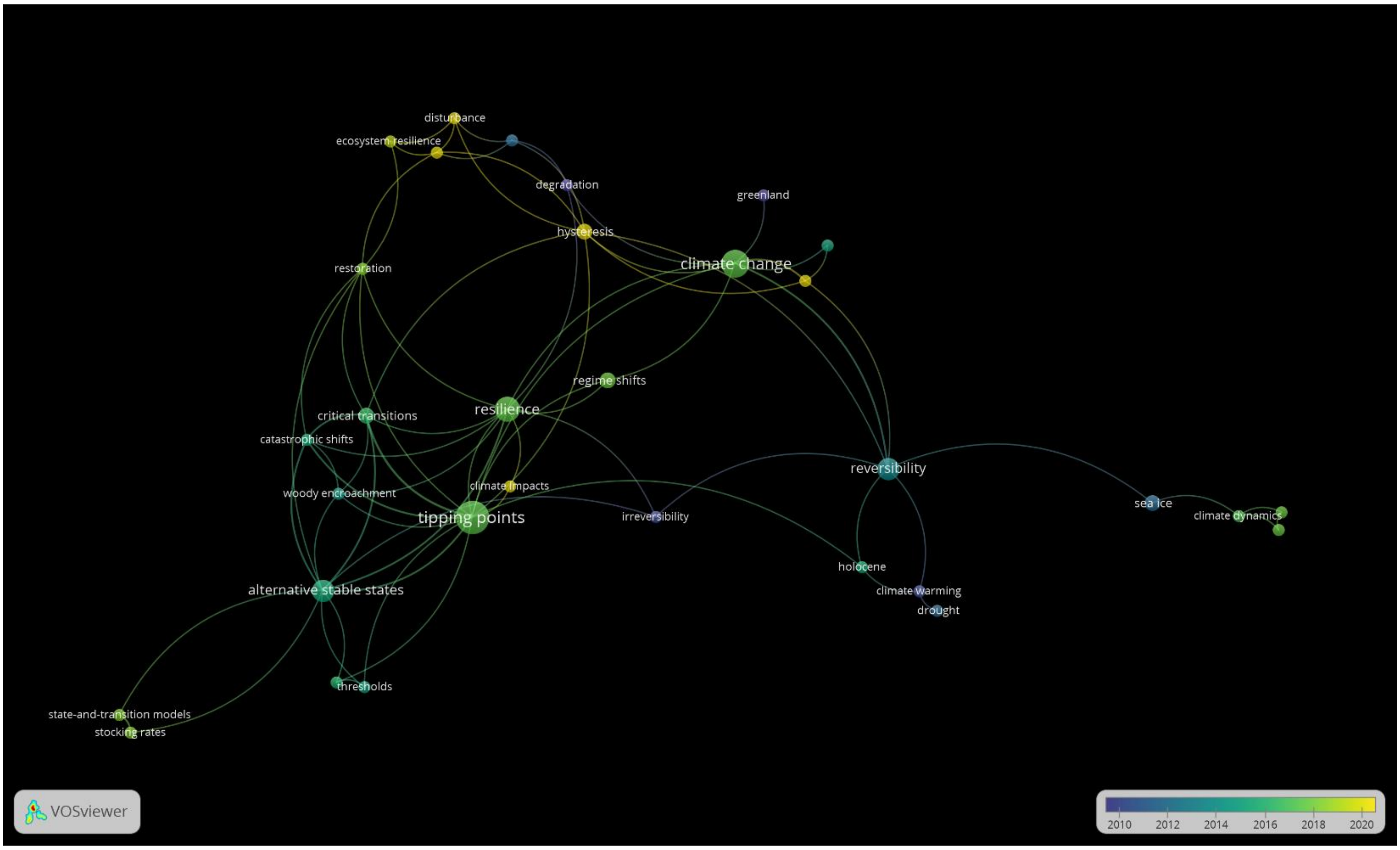
disturbance
ecosystem resilience
degradation
greenland
hysteresis
climate change
restoration
regime shifts
resilience
critical transitions
catastrophic shifts
reversibility
climate impacts
woody encroachment
tipping points
irreversibility
sea ice
climate dynamics
holocene
climate warming
drought
alternative stable states
thresholds
state-and-transition models
stocking rates
VOSviewer
2010
2012
2014
2016
2018
2020

Figure S2 Illustrations of (ir)reversibility (extended analysis/substantiveuse)

Figure R2A The figures (R2Aa-c) are from Scheffer et al (2001)[1] (Copyright © 2001, Macmillan Magazines Ltd.), which is based on Scheffer et al. (2000)[2]. The caption provided by Scheffer at al. (2001) is as follows: "**a** and **b**, only one equilibrium exists for each condition. However, if the equilibrium curve is folded backwards (**c**), three equilibria can exist for a given condition. It can be seen from the arrows indicating the direction of change that in this case equilibria on the dashed middle section are unstable and represent the border between the basins of attraction of the two alternative stable states on the upper and lower branches." **R2B** The figures (R2Ba-b) are from Scheffer et al (2001)[1] (Copyright © 2001, Macmillan Magazines Ltd.). The caption provided by Scheffer at al. (2001) is as follows: "If the system is on the upper branch, but close to the bifurcation point F_2, a slight incremental change in conditions may bring it beyond the bifurcation and induce a catastrophic shift to the lower alternative stable state ('forward shift'). If one tries to restore the state on the upper branch by means of reversing the conditions, the system shows hysteresis. A backward shift occurs only if conditions are reversed far enough to reach the other bifurcation point, F_1. **b**, A perturbation (arrow) may also induce a shift to the alternative stable state, if it is sufficiently large to bring the system over the border of the attraction basin (see also Fig. 3) [here: Figure R2C]." **R2C** The figure is from Scheffer et al (2001)[1] (Copyright © 2001, Macmillan Magazines Ltd.). The caption provided by Scheffer at al. (2001) is as follows: "The bottom plane shows the equilibrium curve as in Fig. 2. The stability landscapes depict the equilibria and their basins of attraction at five different conditions. Stable equilibria correspond to valleys; the unstable middle section of the folded equilibrium curve corresponds to a hill. If the size of the attraction basin is small, resilience is small and even a moderate perturbation may bring the system into the alternative basin of attraction."

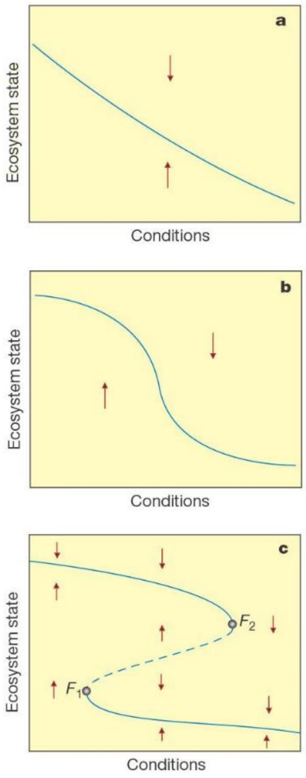

Figure S2A

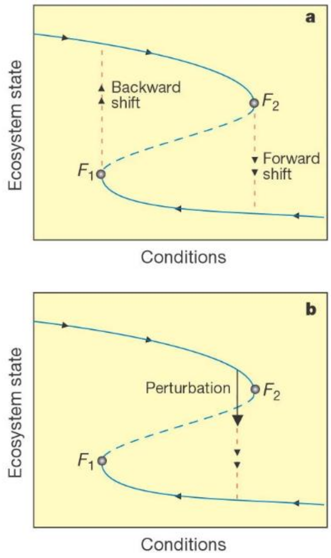

Figure S2B

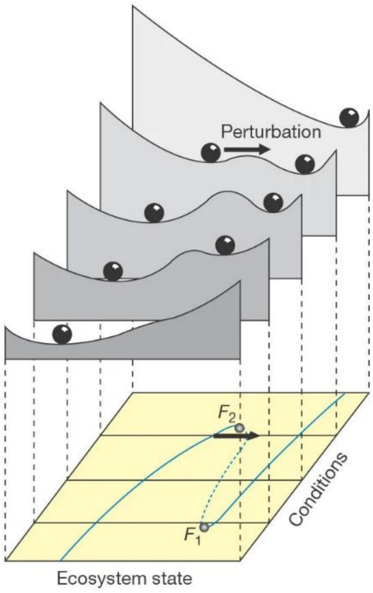

Figure S2C

References Supplementary Material Results

1. Scheffer, M., Carpenter, S., Foley, J. A., Folke, C. & Walker, B. Catastrophic shifts in ecosystems. *Nature* 413, 591–596 (2001). https://doi.org/10.1038/35098000
2. Scheffer, M., Brock, W. & Westley, F. Socioeconomic Mechanisms Preventing Optimum Use of Ecosystem Services: An Interdisciplinary Theoretical Analysis. *Ecosystems* 3, 451–471 (2000).

Supplementary Material: Methods

Contents

Figure S3 PRISMA flow diagram........ 2
Table S4 Eligibility Criteria 3
Table S5 Qualitative content analysis categories of coding and data extraction........ 6
Table S6 PRIMA 2020 checklist *[preliminary]* 9

Figure S3 PRISMA flow diagram

Figure S3 PRISMA Flow diagram. The PRISMA 2020 flow diagram* scheme is used, but modified and adapted to the specificity of selection and analysis. See Figure M2 for inclusion and exclusion decisions, and Table M1 for detailed eligibility criteria.

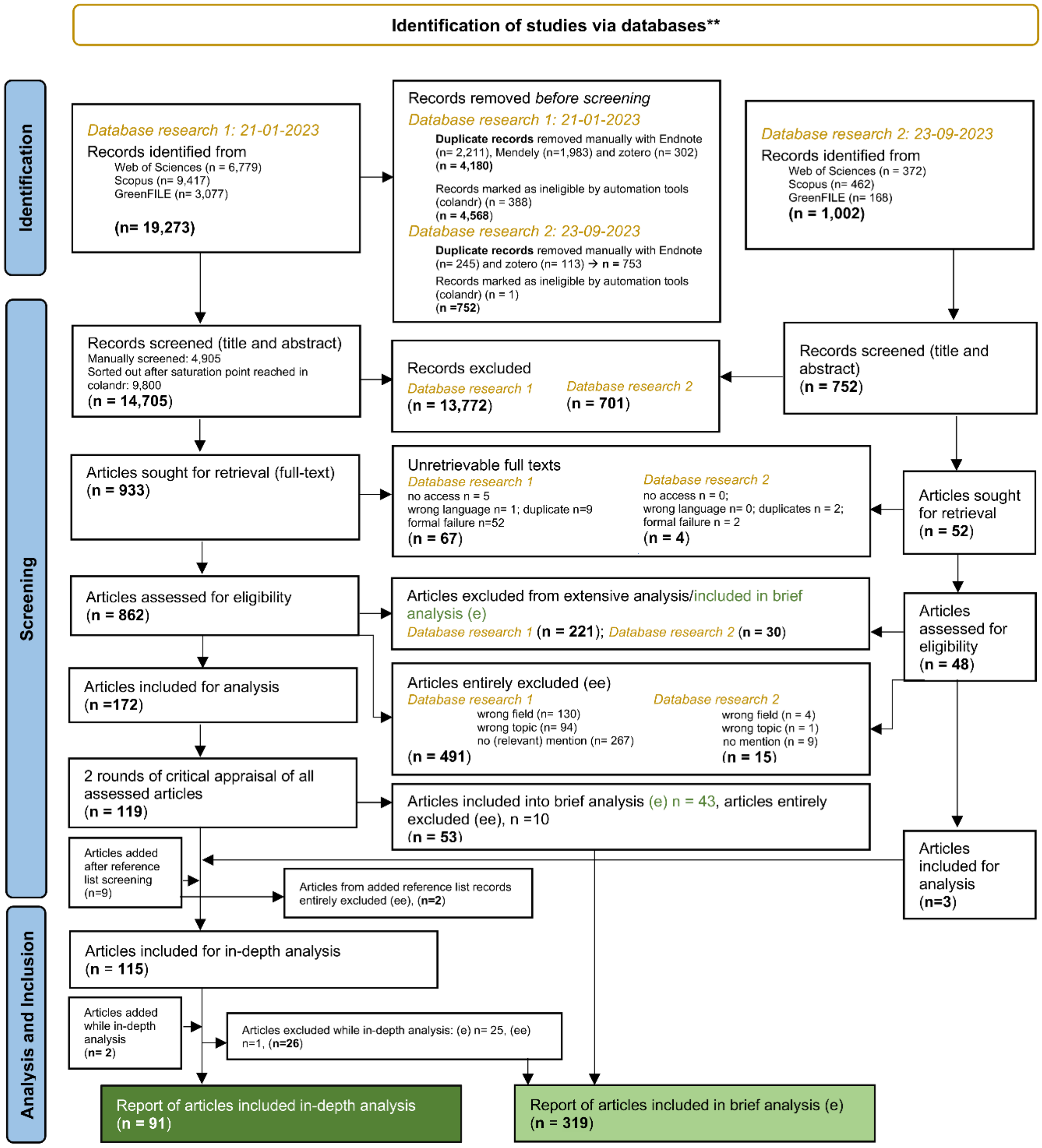

**** Search strings**:

WoS: ((TI=(irreversib* OR (no reversib*) OR "tipping")) OR (KP=(irreversib* OR (no reversib*) OR "tipping")) OR AK=(irreversib* OR (no reversib*) OR "tipping")) AND TS=((climat* OR earth* OR planet* OR natur* OR environment* OR ecosystem* OR ecolog* OR life* OR liv* OR species OR ocean* OR atmospher*))

Scopus: (TITLE-ABS-KEY (climat* OR earth* OR planet* OR natur* OR environment* OR ecosystem* OR ecolog* OR life* OR liv* OR species OR ocean* OR atmospher*)) AND (((TITLE (irreversib*) OR TITLE ((no OR non OR not) PRE/2 reversib*) OR TITLE ("tipping"))) OR ((KEY (irreversib*) OR KEY ((no OR non OR not) PRE/2 reversib*) OR KEY ("tipping")))) AND (LIMIT-TO (LANGUAGE , "English"))

GreenFile: ((TI irreversib* OR "tipping" OR (no* reversib*)) OR (KW irreversib* OR "tipping" OR (no* reversib*)) OR (AB irreversib* OR "tipping" OR (no* reversib*)) AND ((TI climat* OR earth* OR planet* OR natur* OR environment* OR ecosystem* OR ecolog* OR life* OR liv* OR species OR ocean* OR atmospher*) OR (AB climat* OR earth* OR planet* OR natur* OR environment* OR ecosystem* OR ecolog* OR life* OR liv* OR species OR ocean* OR atmospher*) OR (KW climat* OR earth* OR planet* OR natur* OR environment* OR ecosystem* OR ecolog* OR life* OR liv* OR species OR ocean* OR atmospher*) OR (SU CLIMATOLOGY OR CLIMATE CHANGE OR ATMOSPHERE OR EARTH TEMPERATURE OR GEOMORPHOLOGY OR NATURE or ECOLOGY OR LIFE SCIENCES OR SPIECES OR OCEAN))

*Page MJ, McKenzie JE, Bossuyt PM, Boutron I, Hoffmann TC, Mulrow CD, et al. The PRISMA 2020 statement: an updated guideline for reporting systematic reviews. BMJ 2021;372:n71. doi: 10.1136/bmj.n71

Table S4 Eligibility criteria

Table S4 Eligibility criteria for screening and selection. The criteria were refined during the screening process.

<table>
<tr><th>Inclusion criteria</th><th>Exclusion criteria</th></tr>
<tr><td>
<u>I. Research Areas:</u>

Earth (System) Sciences
Geo Sciences, Environmental Sciences and involved disciplines, such as geochemistry and geophysics (can be supported by mathematical modelling)
Ecology as subdiscipline of Biology (with a focus on the biosphere; can be supported by mathematical modelling, can be on a purely theoretical level, ecosystem health and resilience, ecosystem management concerned with particular species from a biological perspective)
Biology including genetics (genetics when related to anthropogenic extinction and alteration of evolution)
Intersectional and multidisciplinary research fields and studies, such as Life Sciences and Agriculture, Forestry, Biodiversity Conservation, Meteorology, Atmosphere Sciences, Oceanology (the overall angle must not be in Social Sciences; no socio-ecological frameworks)

Note: For interdisciplinary und transdisciplinary texts it is important to identify the primary/leading discipline(s) in the record: What is the overall disciplinary perspective? What kind of knowledge is being produced: knowledge within/for the social sciences or within/for the natural and geo and Earth system sciences and/or for a broader audience? → commentary articles in which natural, geo and environmental scientists providing (natural) scientific evidence for political action taking are included (and coded with ‘policy’)

<u>II. Topics:</u>

anthropogenic climate change
anthropogenic changes in the Earth system’s spheres/Earth’s systems (cryosphere, lithosphere, biosphere, atmosphere, hydrosphere)
human-driven environmental changes, i.e., changes in eco systems/in the biosphere, regarding biodiversity
Anthropocene

</td><td>
<u>I. Research Areas and Fields of</u>

Theoretical physics and chemistry (i.e., general hypotheses in the field of thermodynamics, kinetics, and dissipative systems)
Basic research in inorganic and organic chemistry
‘Pure’ Mathematics
Material Sciences
Engineering
Energy Sciences
Pharmacology
Molecular and cell biology when not applied to extinction and evolutionary processes
Biochemistry when not applied to extinction and evolutionary processes
Medicine
Physiology
Veterinary
Social Sciences
Political and Social Ecology [= overall angle and audience is in Social Sciences, e.g., economically driven ecosystem services or management assessments/models; institutional drivers; economical sustainability; based on social-ecological frameworks]
Ecosystem management and landscape planning in terms of natural resources for human development
Economics
Law
Humanities
Philosophy (incl. Ethics)
Human Geography
Urban and regional planning
Anthropology
Archaeology

(= wrong field)

<u>II. Topics:</u>

All types of general, theoretical, nonorganic, or molecular-based branches of disciplines in the ‘hard’ natural sciences (physics, biology, chemistry)
all types of studies that do not involve anthropogenic factors (e.g. natural hazards such as earthquakes, volcanic eruption, tectonics)
all types of studies that primarily investigate technical instruments/apparatuses of measurement from a rather technical or operational angle
Irreversibility primarily demonstrated in or by mathematical, molecular, quantum or other microphysical modelling or testing.
Attitude and perception studies on ecological issues

</td></tr>
</table>

	• Studies for the purpose of improving resource exploitation<br>• Studies on research for optimising monitoring or management in terms of social involvement<br>• Educational papers<br>• Human extinction<br><br>(= wrong topic I)
III. Occurrence and Usage [mainly relevant for screening step 2]:<br><br>• Occurrence of the notion of irreversibility/irreversible/ reversibility/reversible in the full text<br>• functional usage of irreversibility (e.g., as part of definition)<br>• Definition, conceptualization, or substantial inquiry of (ir)reversible changes<br>• Definition, conceptualization, or substantial inquiry of (ir)reversible impacts of technological interventions<br>• Definition, conceptualization, or inquiry of tipping points/elements/events (‘tipping subjects’)<br><br>= conceptual and topical context according to the target research fields and topics<br><br>+++ **General rule** for title-abstract screening (screening step 1): rather generous inclusion for full-text screening, marked with the label “uncertain” +++	III. Occurrence and Usage [mainly relevant for screening step 2]:<br><br>• No or unspecified occurrence of the notion of irreversibility/irreversible/reversibility or “tipping” in the full text<br>• Wrong meaning/sense of ‘to revers’, ‘revers’, ‘reversal’, e.g. used in a technical sense of ‘reversing the parameter’, ‘the reverse is treue’, ‘reversing’ a trend or direction<br><br>(= wrong topic II)
IV. Types of records:<br>• Published scientific articles, papers, opinions, editorials, and commentary<br><br>Note: The texts have not necessarily be peer-reviewed	IV. Types of records:<br>• Non-scientific or non-professional papers or statements<br>• policy briefs, policy statements<br>• unpublished texts grey literature; preprints<br>• duplicates (= duplicate)<br>• not accessible via licences (no access)<br><br>Note: When there are two versions of a paper, a proceeding/working paper version and a peer-reviewed journal-based publication, the latter is included.<br><br>(= formal failure)
V. Language:<br>English	V. Language:<br>Language other than English (= wrong language)

List of inaccessible articles:

1. Budzianowski, W. Tetra-stable bifurcation structure of the climate system of Earth: Mechanisms triggering potential transition to the greenhouse steady state. *Int. J. of Global Warming* **5**, 152–178 (2013).
2. Buravlev, E P (1989), Estimating the ecological characteristics of the human environment, (reference data incomplete)
3. Hanson D, Fast Action Urged on Climate Change. *Chem. Eng. News Archive* **85**, 7 (2007).
4. Ilnicki, P. & Zeitz, J. Irreversible Loss of Organic Soil Functions after Reclamation. in *Organic Soils and Peat Materials for Sustainable Agriculture* (CRC Press, 2002)
5. Michaeli, E., Ivanova, M., Solár, V. & Ech, V. The Ecological Stability Evaluation of the Landscape (Case study from the Eastern Slovakia). **1**, 695–702 (2014).

Table S5 Qualitative content analysis categories of coding and data extraction

Table S5 Qualitative content analysis categories of coding and data extraction. **a)** Codebook and extraction for the in-depth analysis of articles with substantive use of the target terminology. **b)** Codebook and extraction for brief analysis of articles with non- substantive use of the target terminology.

a) Codebook and extraction for the in-depth analysis of articles with substantive use of the target terminology

Research interest	Categories of coding (bold) and extraction	Explication or example
	authors, title, publication year, abstract	Categories of basic meta data extraction
Research topic	**0_topic**	
Specification of the subject	**1_attribute**	What's the subject of irreversibility?
	**2_referred entity/relation**	e.g. climate, ice, ecosystem, extinction, ice, marine
	2_specification	
	**3_attributive notions related** to irreversibility	e.g. abrupt, disruptive, nonlinear
	**4_spatial scale** of entity	local, regional, global
	4_specification	e.g., Amazon, Arctic; subcontinental
	**5_temporal scale of irreversibility**	
Conceptual	**6_conceptual function**	e.g. element of the definition of tipping points or ecological thresholds
	6_conceptual function: **definiendum**	(ir)reversibility are defined
	6_**conceptual innovation**	a new concept entailing (ir)reversibility is given
	**6_key quotation(s)**	References provided for key definitions around tipping points, thresholds, and irreversibility
	**6_key references**/authors for the definition/conceptual use	
	**6_visualisation**	Are there visualisation strategies applied? E.g. the basin-ball-diagram?
	6_**research question** or hypothesis	Here, it's interesting to see if the research question or hypothesis contains the notions irreversibility or reversibility
	**7_scientific paradigm**/theory/framework	e.g. thermodynamics, catastrophic transitions theory
Normative implications	**8_emergency** or urgency frame	**Statement about urgent action need or emergency case regarding**
	**9_normativity**	Type a), b), c, d) (see below)
	**9_normativity_message**	statements on rights and wrongs in the way issues of anthropogenic change are discussed (=normative statement type e)
	**10_goals**	Politically agreed goals or frameworks of management; e.g. SDGs; Paris Agreement
formal	**11_type of research/text**	e.g. empirical, modelling, theoretical, review
Extension: the coding and understanding of normative statements		
• Normative statements type a): Normativity in terms of ecosystem functionality and structure (**'ecological' or 'systemic environmental normativity'**), i.e., an ecosystem is subject to change in its functionality and/or structure, described in terms of, degradation or decline [default assumption in ecology]; not: ice mass loss (a technical term)		

Irreversible change or the expected new state of an ecosystem can be *evaluated* with regard to ecosystem functionality, structure and composition ('evaluative ecological normativity'). Evaluative normativity is expressed by using terms such as 'desirable', 'negative effects/impacts on', 'non-desirable', 'harmful' states or regimes of ecosystems. In some articles, the object of evaluation remains open.

- Normative statements type b): Normativity in terms of harms to human well-being, societies, or economies when ecosystems are irreversibly degrading, or climate tipping elements are expected to cross critical thresholds ('**social normativity**'). Social normativity is also evaluative and there can be overlap with rather ecological evaluative normativity. In contrast to evaluative ecological normativity, socially oriented normative statements explicitly express negative impacts on human societies.
- Normative statements type c): Research findings are explicitly designated as valuable knowledge to inform risk assessment, ecosystem management or policy, in other words, the research findings presented in the paper *should* inform social (incl. economic, agricultural) risk assessment, managerial, social or political decision making ('**epistemic normativity**').
- Normative statements type d): When explicit or rather implicit management or **policy recommendations** are given, sometimes referred to as 'political implications' by the authors in the final section of a paper ('policy normativity')
- Normative statements type e): Additionally, we found a fifth type of statement, which we call '**message to the public'**. We understand them as statements about rights and wrongs in the way issues of anthropogenic change are discussed. These statements refer to an 'ought' in the sense of how issues and problems should (not) be better discussed and understood, thereby not necessarily addressing a specific actor).

Clarification 1
The normative statements can be clustered, building a 'type of normativity'. All clustered normative statements taken into account, we are dealing with four types of normativity, namely, ecological normativity, social normativity, epistemico-political and action-oriented normativity, whereof the two latter explicitly address management and political decision-makers, either as epistemic (knowledge-oriented) advise (c), or in the form of policy recommendations (d).
Clarification 2:
The normative statements are not inclusive or ordered by degree, that is c) and d) type normative statements do not necessarily include the occurrence of a) and b)-type normative statements in the same article.
Clarification 3:
Whereas ecological normativity (type a) refers to ecosystem norms and the avoidance of failing these norms (e.g. ecosystem functionality, structure, or a certain composition), social normativity implicitly refers to avoidance of harms through adequate action-taking (that is social normative statements declare harms to human society and by this implicitly refer to the maxim/principle of avoidance or reduction of harm). In contrast, epistemico-political (c) and political normativity (d) express action-oriented attitude or action-guiding claim about required/needed action or guidance.

Clarification 4:
The fifth type of normative statements (e) express a message to the public or decision-makers below the threshold of a policy recommendation'. These statements fall somewhere between type c) normative statements and implicit policy recommendations.

Examples of normative statements type b, c, d:

- Example of an implicit normative statement type b: "These global hotspots of irreversible changes can indicate *elevated risks of negative impacts* on developing countries." [102] (I consider the phrase "elevated risks of negative impacts" to express an evaluation of harm to human well-being, here 'countries').
- Example of an explicit normative statement type c: "The application of these concepts in future research and management applications should include evidence on the mechanistic links between pressures and consequent ecological change." [43]
- Example of an descriptive (i.e. not normative) statement: "An abrupt climate change might directly force an abrupt change in an Earth or human system." [149]
- Example of an implicit normative statement type d): "Given the irreversibility of CO2-induced warming (5, 6), every increment of avoided temperature increase represents less warming that would otherwise persist for many centuries. Although emissions reductions cannot return global temperatures to preindustrial levels, they do have the power to avert additional warming on the same time scale as the emissions reductions themselves. Climate warming tomorrow, this year, this decade, or this century is not predetermined by past CO2 emissions; it is yet to be determined by future emissions. The climate benefits of emissions reductions would thus occur on the same time scale as the political decisions that lead to the reductions." [147]
- Example of an xplicit normative statement type d): "A constructive way forward would be to engage the people of the Arctic region in the geoengineering debate through the Arctic Council (Egede-Nissen and Venema 2009)." [122]

b) Codebook and extraction for brief analysis of articles with non- substantive use of the target terminology

Research interest	Categories of coding (bold) and extraction	Explication or example
	authors, title, publication year, abstract	categories of basic meta data extraction
Research topic	**0_topic**	according to the subject and topics A to D
	**0_domain**	environmental domain, e.g. biosphere, cryosphere
conceptual	**1_potentiality**	the target terminology is used in a statement expressing a possibility, potentiality, risk of irreversibility
	**1_probability**	the target terminology is used in a statement expressing a probability or prediction of irreversibility
	**1_empirical**	the target terminology is used in a statement expressing an empirical description irreversibility or irreversible change
	**1_conceptual**	the target terminology is used in a technical conceptual sense, i.e. in a referenced definition of critical transition or tipping point
	**2_specification**	a temporal or spatial or other specification of the term is given
normative	**3_urgency**	urgency to take action is stated
	**4_warning**	warning against negative consequences of irreversible change or emphasis of negative impacts

Table S6 PRIMA 2020 checklist *[preliminary]*

Table S6 PRISMA 2020 checklist. We modified the checklist with the inclusion of some (sub-)items and elements (marked in orange) from the PRISMA 2020-EcoEvo checklist; N.A. =not applicable (as the focus is on conceptual usages and not on empirical evidence), SI= Supplementary Information.

Section and Topic	Item #	Checklist item	Where reported	Notes
**TITLE and ABSTRACT**				
Title and abstract	1.1	Identify the report as a systematic review.		The style of the title depends on the journal's re-quirements.
	1.1	Summarise the aims and scope of the review	p. 1	
	1.2	Describe the data set	p. 1	
	1.3	State the results of the primary outcome	p. 1	
	1.4	State conclusions	p.1	
**INTRODUCTION (Main)**				
Rationale	2.1	Provide a rationale for the review	p. 2	
	2.2	Reference any previous reviews or meta-analyses on the topic.	p. 2	
Objectives	2.3	Provide an explicit statement of the objective(s) or question(s) the review addresses.	p. 2	
**METHODS**				
Eligibility criteria	3.1	Report the specific criteria used for including or excluding studies when screening titles and/or abstracts, and full texts, according to the aims of the systematic review (e.g. study design, taxa, data availability)	p. 9	+ Box 1, Table S4
	3.2	Justify criteria, if necessary (i.e. not obvious from aims and scope)	p. 9	+ Figure S4
Information sources, finding articles	4.1	Specify all databases, registers, websites, organisations, reference lists and other sources searched or consulted to identify studies. Specify the date when each source was last searched or consulted.	p.9	
	4.2	Define the type of search (e.g. comprehensive search, representative sample)	p.9	
	4.3	State what sources of information were sought (e.g. published and unpublished studies, personal communications)	p. 9	+ Figure S3
	4.4	Provide enough information to repeat the equivalent search (if possible), including the timespan covered (start and end dates)	p. 9	
Search strategy	5	Present the full search strategies for all databases, registers and websites, including any filters and limits used.	p. 9, Figure S3	+ Supplementary Material 2
Selection process	6	Specify the methods used to decide whether a study met the inclusion criteria of the review, including how many reviewers screened each record and each report retrieved, whether they worked independently, and if applicable, details of automation tools used in the process.	p. 9-10	
Data collection process	7	Specify the methods used to collect data from reports, including how many reviewers collected data from each report, whether they worked independently, any processes for obtaining or confirming data from study investigators, and if applicable, details of automation tools used in the process.	p. 10	
Data items	8.1	List and define all outcomes for which data were sought. Specify whether all results that were compatible with each outcome domain in each study were sought (e.g. for all measures, time points, analyses),	Reference Lists S1, S2	

Section and Topic	Item #	Checklist item	Where reported	Notes
		and if not, the methods used to decide which results to collect.		
	8.2	List and define all other variables for which data were sought (e.g. participant and intervention characteristics, funding sources). Describe any assumptions made about any missing or unclear information.	p. 11<br>Table S5	
Study risk of bias assessment	9	Specify the methods used to assess risk of bias in the included studies, including details of the tool(s) used, how many reviewers assessed each study and whether they worked independently, and if applicable, details of automation tools used in the process.	9-10	
Effect measures	10	Specify for each outcome the effect measure(s) (e.g. risk ratio, mean difference) used in the synthesis or presentation of results.	N.A.	
Synthesis methods	11.1	Describe the processes used to decide which studies were eligible for each synthesis (e.g. tabulating the study intervention characteristics and comparing against the planned groups for each synthesis (item #6)).	N.A.	
	11.2	Describe any methods required to prepare the data for presentation or synthesis, such as handling of missing summary statistics, or data conversions.	/	
	11.3	Describe any methods used to tabulate or visually display results of individual studies and syntheses.	p. 10-11	For further details see the caption of Figure S1 (main text)
	11.4	Describe any methods used to synthesize results and provide a rationale for the choice(s). If meta-analysis was performed, describe the model(s), method(s) to identify the presence and extent of statistical heterogeneity, and software package(s) used.	p. 10-11	
	11.5	Describe any methods used to explore possible causes of heterogeneity among study results (e.g. subgroup analysis, meta-regression).	/	
	11.6	Describe any sensitivity analyses conducted to assess robustness of the synthesized results.	N.A.	
Reporting bias assessment	12	Describe any methods used to assess risk of bias due to missing results in a synthesis (arising from reporting biases).	/	p. 11 (limitations)
Certainty assessment	13	Describe any methods used to assess certainty (or confidence) in the body of evidence for an outcome.	N.A.	
**RESULTS**				
Study selection	14.1	Describe the results of the search and selection process, from the number of records identified in the search to the number of studies included in the review, ideally using a flow diagram.	Figure S3	
	14.2	Cite studies that might appear to meet the inclusion criteria, but which were excluded, and explain why they were excluded.	/	See the note on 'technical' or inadequate sense of 'reversing' in Box 1, Table S4.
Study characteristics	15	Cite each included study and present its characteristics.	Reference List (RFL) S1	See RFL2 for articles included into brief analysis
Risk of bias in studies	16	Present assessments of risk of bias for each included study.	N.A.	
Results of individual	17	For all outcomes, present, for each study: (a) summary statistics for each group (where appropriate) and (b) an effect estimate and its precision (e.g. confidence/credible interval), ideally using structured	N.A.	

Section and Topic	Item #	Checklist item	Where reported	Notes
studies		tables or plots.		
Results of syntheses	18.1	For each synthesis, briefly summarise the characteristics and risk of bias among contributing studies.	N.A.	
	18.2	Present results of all statistical syntheses conducted. If meta-analysis was done, present for each the summary estimate and its precision (e.g. confidence/credible interval) and measures of statistical heterogeneity. If comparing groups, describe the direction of the effect.	Table 1, Figure 1	
	18.3	Present results of all investigations of possible causes of heterogeneity among study results.	N.A.	
	18.4	Present results of all sensitivity analyses conducted to assess the robustness of the synthesized results.	N.A.	
Reporting biases	19	Present assessments of risk of bias due to missing results (arising from reporting biases) for each synthesis assessed.	/	
Certainty of evidence	20	Present assessments of certainty (or confidence) in the body of evidence for each outcome assessed.	N.A.	
**DISCUSSION**				
Discussion	21.1	Provide a general interpretation of the results in the context of other evidence.	p. 6-9	
	21.2	Discuss any limitations of the evidence included in the review.	N.A.	
	21.3	Discuss any limitations of the review processes used.	p. 11	See the subsection in the Methods part
	21.4	Discuss implications of the results for practice, policy, and future research.	p. 8-9	+ Figure 3
References	22.1	Provide a reference list of all studies included in the systematic review or meta-analysis	RFL S1, RFL S2	
	22.2	List included studies as referenced sources (e.g. rather than listing them in a table or supplement)	p. 11-13	
**OTHER INFORMATION**				
Registration and protocol	23.1	Provide registration information for the review, including register name and registration number, or state that the review was not registered.	9	
	23.2	Indicate where the review protocol can be accessed, or state that a protocol was not prepared.	p. 9	
	23.3	Describe and explain any amendments to information provided at registration or in the protocol.	/	
Software	24	Describe the version numbers of all software used.	p.10	
Support	25	Describe sources of financial or non-financial support for the review, and the role of the funders or sponsors in the review.		
Contributions and competing interests	26.1	Declare any competing interests of review authors.	p. 13	
	26.2	Provide names, affiliations, and funding sources of all co-authors		
	26.3	List the contributions of each co-author		
	26.4	Provide contact details for the corresponding author		
Availability of data, code and other materials	27	Report which of the following are publicly available and where they can be found: template data collection forms; data extracted from included studies; data used for all analyses; analytic code; any other materials used in the review.	Table S5	

References

1. O'Dea, R.E., Lagisz, M., Jennions, M.D., Koricheva, J., Noble, D.W., Parker, T.H., Gurevitch, J., Page, M.J., Stewart, G., Moher, D. and Nakagawa, S. (2021), Preferred reporting items for systematic reviews and meta-analyses in ecology and evolutionary biology: a PRISMA extension. Biol Rev. doi:10.1111/brv.12721
2. Page, M. J. *et al.* The PRISMA 2020 statement: an updated guideline for reporting systematic reviews. *BMJ* **372**, n71 (2021).

Supplementary Material: Reference Lists (RL)

Reference List S1 Articles with substantive uses of reversibility/reversible, irreversibility/irreversible (in-depth analysis) 2
Reference List S2 Articles with non-substantive use of reversibility/reversible, irreversibility/irreversible (brief analysis) 25

Reference List S1 Articles with substantive uses of reversibility/reversible, irreversibility/irreversible (in-depth analysis)

Reference List S1 List of articles with substantive use included into in-depth analysis including charted data. Articles were included in the in-depth analysis if they show substantive use of ir/reversibility terminology. For each included article, the environmental domain(s), subject area(s) and topic(s) were identified, on this base a thematic scheme was developed (A to D). The columns represent most of the categories for coding, some categories have been merged, see Supplemental Material Results Table M2a) for the coding categories. The columned on normativity indicated whether the paper gives explicit management or policy recommendations (normativity type d) or addresses the way scientific or public discourse should (better) be conducted (normativity type e).

**Subject areas and topics**	**Environmental domain**
**A. Systemic transition**<br>A.1 Ecological thresholds/regime shifts/tipping points<br>A.2 Tipping points and elements and threshold behaviour in/of the Earth system(s)<br>A.3 General approaches to environmental changes<br>**B. Impacts and responses**<br>B.1 Impacts of greenhouse gas emissions<br>B.2 Impacts as consequences of climate change<br>B.3 Direct impacts of human activity<br>**C. Evolution and extinction**<br>**D. Scenarios of reversal intervention**<br>D.1 Biological conservation<br>D.2 Ecosystem restoration, recovery<br>D.3 Climate engineering	biosphere<br>geosphere<br>hydrosphere, cryosphere<br>atmosphere, climate<br>across<br>theoretical, conceptual

Supplementary Material 1

	Pdf no<br>Topic	**Reference**	**Usage of the notions of irreversibility and reversibility and related issues**							**The paper…**		
			conceptual function/usage<br>(if definiendum or conceptual innovation see SM Table XX)	the subject of ir/reversibility	temporal and/or spatial definition of specification	research question or hypothesis containing ir/reversibility	theoretical framework or paradigm	visualisation of reversibility and/or irreversibility	key quotation	has normati vity type d or e	uses an urgenc y frame (yes/no)	type of research or text
1	21<br>A.2	Armstrong McKay, D. I. et al. Exceeding 1.5°C global warming could trigger multiple climate tipping points. Science 377, eabn7950 (2022)	ir/reversibility as a key criterion in the extension of the definition of climate tipping points	tipping points	ethical time horizon of 10k years, political horizon of 100 years; critical time: by 2100<br>1 million km2 or above - at least regional	no	environmental tipping points and elements	no	“(Ir)reversibility. Tipping points usually lead to irreversible qualitative change but reversible tipping points are possible as a special case (1) […].” (p. 2)	yes	yes	review (assessment)
2	35<br>A.1	Viglizzo, E. F., Nosetto, M. D., Jobbágy, E. G., Ricard, M. F. & Frank, F. C. The ecohydrology of ecosystem transitions: a meta-analysis. Ecohydrology 8, 911–921 (2015).	ir/reversibility as a key category to describe findings of modelling of ecosystem transitions from the perspective of ecohydrology	ecological regimes	human lifetime<br>regional	no	nonlinear dynamics	ball-and-basin diagram	" [...] (iii) a uni-modal, irreversible dynamics from grasses to shrubs in response to stressors such as overgrazing is likely to occur in low-rainfall regions." (p. 917)	no	no	empirical; data-driven meta-analysis; modelling
3	43<br>A.1<br>A.3	Capon, S. J. et al. Regime shifts, thresholds and multiple stable states in freshwater ecosystems; a critical appraisal of the evidence. Science of The Total Environment 534, 122–130 (2015).	irreversibility as a definiens in the definition of ecological regime shifts	ecosystem states	no	no	multiple-stable-state theory	ball-and-basin diagram	"[W]e retain the term ‘regime shifts’ to describe the process by which ecosystems change state irreversibly across thresholds or tipping point." (p. 124)	no	yes	review on empirical evidence

4	54<br>A.1	Gianfreda, L. & Rao, M. A. Interactions Between Xenobiotics and Microbial and Enzymatic Soil Activity. Critical Reviews in Environmental Science and Technology 38, 269–310 (2008).	ir/reversibility as definiens and key criterion in the differentiation between reversible and irreversible effects of xenobiotic pollution	Alteration; change of natural environmental balance; effects	no<br>global/general	no	frameworks that assess biodegradability	no	“Xenobiotics may have direct and indirect effects on enzyme activities... A direct irreversible effect, instead, will lead to an irreversible inhibition of the enzymatic action by covalent binding of the xenobiotic (or its derivatives) with functional, catalytic enzymatic groups.” (p. 286)	yes	no	review (assessment)
5	55<br>A.3	Gibbins, G. & Haigh, J. D. Entropy Production Rates of the Climate. Journal of the Atmospheric Sciences 77, 3551–3566 (2020).	irreversibility used in the development of the concept of global entropy production (= definitional extension)	processes that transfer heat within the climate	no<br>global/general	no	2nd law of thermodynamics, theory of entropy production	no	"The climate consists of energy transfers that are mediated by irreversible processes, such as rain, wind, radiation." (p. 3551)	no	no	theoretical
6	56<br>A.2	Good, P. et al. Recent progress in understanding climate thresholds: Ice sheets, the Atlantic meridional overturning circulation, tropical forests and responses to ocean acidification. Progress in Physical Geography: Earth and Environment 42, 24–60 (2018).	irreversibility as a key aspect in the characterisation of research findings (assessment of climate tipping points)	loss; collapse	millennia (varying timescales depending on the system)<br>regional<br>(Greenland, East and West Antarctic Ice Sheets)	no	no	no	“For ice sheets, some degree of irreversible loss (timescales of millennia) of part of the West Antarctic Ice Sheet (WAIS) may have already begun, but the rate and eventual magnitude of this irreversible loss is uncertain.” (p. 25)	no	no	review (assessment)
7	59<br>A.1<br>A.3	Grainger, A. The prospect of global environmental relativities after an Anthropocene tipping point. Forest Policy and Economics 79, 36–49 (2017).	i/reversibility as a key criterion to characterise research findings in the assessment of tipping points	tipping points	no passage of irr. TP in the next few decades; consequences for hundreds of years, perhaps millennia<br>regional (Amazonian forests)	“This framework generates the hypothesis that above a threshold level of carbon dioxide in the atmosphere the planet will pass through a reversible tipping point and move from the special condition of relative ‘stationarity’ in climate […].” (p. 37)	Anthropocene determination debate	no	“Another weakness in the existing three stage chronology is a lack of clarity in how its key concepts of ‘criticality’ and an ‘irreversible threshold’, or ‘tipping point’, are linked together in time and by various mechanisms" (p. 39)	yes	yes	conceptual

8	73<br>A.2	Heinze, C. et al. The quiet crossing of ocean tipping points. Proceedings of the National Academy of Sciences 118, e2008478118 (2021).	ir/reversibility as a key criterion in the characterisation of research findings on tipping points in the ocean and as a component in the definitional extension of abrupt changes	alteration of the deep ocean; anthropogenic environmental changes	"human time scale"<br>global (but local reversal of deoxygenation is possible)	no	no	no	"Abrupt changes can potentially be reversible, so that one system state occurs for a unique type of conditions directly following the forcing. […] In this case [hysteresis], a certain system state is not coupled to one unique forcing. Strong negative forcing may be needed to enable a return to the initial system state (or such a return may be completely impossible in case of irreversibility)." (p. 3)	yes (a list of policy recomm endatio ns)	no	assessment
9	102<br>B.1<br>D.3	Kim, S.-K. et al. Widespread irreversible changes in surface temperature and precipitation in response to CO2 forcing. Nat. Clim. Chang. 12, 834–840 (2022).	ir/versibility as the key criterion for (simulation) research on global and regional climate; reversibility as definiendum; irreversibility as definiendum	global surface temperatures due to climate change; precipitation patterns	centuries - irreversibility of temperature and precipitation, "human-perceptible timescale"<br>global, regional	"[H]ow do the global patterns of hysteresis and reversibility look; which specific regions of the world exhibit irreversible changes; what is the key mechanism that shapes global hysteresis and reversibility patterns?" (p. 834)	Earth system science; climate science	conceptual framework for climate hysteresis and reversibility: schematic state-driver diagram of hysteresis	"[A] climate system can follow a distinct trajectory during periods of greenhouse gas emission and removal. Such a path-dependent behaviour is referred to as hysteresis. Similarly, the ability for the climate system to be restored to its initial state is referred to as reversibility." (p. 834)	no	no	modelling
10	107<br>A.1	Kramer, I. & Mau, Y. Soil Degradation Risks Assessed by the SOTE Model for Salinity and Sodicity. Water Resources Research 56, e2020WR027456 (2020).	irreversibility as key criterion for modelling of soil degradation; reversible processes (and hysteresis) as definiendum	soil salinity; soil processes; degradation; condition; changes	unclear; implied meaning: permanent<br>regional (data from Portugal, Israel)	no	ecology, natural resource management	model simulations	"In this paper, we also demonstrated how consideration of irreversibility in Ks radically altered the probability of degradation in our simulations. A better account of the partial reversibility of Ks must be developed and incorporated to all models if we are to accurately assess long-term degradation risks." (p. 15)	no	no	modelling
11	119<br>A.2	Lenton, T. Tipping Elements from a Global Perspective. in Addressing Tipping Points for a Precarious Future (eds. O'Riordan, T. & Lenton, T.) 0 (British Academy, 2013). doi:10.5871/bacad/97801972655 36.003.0002.	ir/reversibility as a key aspect of the definitional extension + as a key category for research assessment of tipping points + reversibility as definiendum	climate system, tipping points	policy-relevant timescale: "this century", a 1000 year ethical time horizon<br>global	no	applied physical theory; dynamical systems theory; earth system science; bifurcation theory in mathematics and physics	ball-and-basin-diagram	"Tipping point change also includes transitions that are slower than their cause (in both cases the rate is determined by the system itself). In either case the change in state may be reversible or irreversible. Reversible means that when the forcing is returned below the tipping point the system recovers its original state (either abruptly or gradually). Irreversible means that it does not (it takes a larger change in forcing to recover). Reversibility in principle does not mean that changes will be reversible in practice." (p. 25-6)	yes	yes	risk assessment, summary

12	122<br>A.2	Lenton, T. M. Arctic Climate Tipping Points. AMBIO 41, 10–22 (2012).	irreversibility as a component of the definitional extension of tipping points: abrupt or/and irreversible; differentiation between reversible and irreversible tipping points	meltdown; transition; change; tipping point; bifurcation	years to centuries; Greenland Ice Sheet as a permanent irreversible loss	"What shapes global hysteresis and reversibility patterns? We specifically focus on the response of two key climatic variables—surface temperature and precipitation—to CO2 forcing.") (p. 834)	Earth system science; climate science	parameter-state-diagrams	"a 'tipping point' need not involve an irreversible bifurcation (Lenton et al. 2008)" (p. 16)	no	yes	theoretical, risk assessment
13	124<br>A.3<br>D.3	Lenton, T. M. Can emergency geoengineering really prevent climate tipping points? in Geoengineering our Climate? (Routledge, 2018).	ir/reversibility of changes as key criterion for the evaluation of climate engineering	change	years to centuries	no	Earth system science; climate science	bifurcation diagram	"Once in this alternative state, the system has to be taken to a different and distant tipping point to trigger recovery. Even after that, it is not back where it started. Irreversibility is even stronger in the case of tipping points in ecological systems, for example dieback of the Amazon or boreal forests (Lenton et al., 2008)." (p. 44)	yes	yes	perspective
14	126<br>A.2	Lenton, T. M. et al. Tipping elements in the Earth's climate system. Proceedings of the National Academy of Sciences 105, 1786–1793 (2008).	ir/reversibility as a key element in the extended definition of tipping elements compared to tipping points + as a key criterion for the assessment of phase changes: there are reversible and irreversible phase changes	phase transitions/changes	''political time horizon'' (TP) = 100y, critical time within this millennium by 2100; ''ethical time horizon'' (TE) ~ 1,000 y;<br>at least subcontinental in scale (length scale of order ≈1,000 km)	no	Earth system science; climate science, bifurcation theory	ball-and-basin-diagram; time series function	"We have formulated a much broader definition of a tipping element, because [...] (iv) for several important phase changes, state-of-the-art models differ as to whether the transition is reversible or irreversible (in principle)" (p. 1786)	yes	yes	review
15	145<br>D.2	Mao, F. & Richards, K. Irreversible river water quality and the concept of the reference condition. Area 44, 423–431 (2012).	i/reversibility as the key criterion for (meta-)analysis of the quality change of river ecosystems	water quality after intensive agricultural; colonization	historical baselines of river water quality - changes following industrial agriculture found to be practically irreversible on "relevant timescales"<br>local, regional	"The aim of this paper is to analyse whether it is appropriate to regard a reference condition (or any status determined relative to a reference condition) as a recovery target, and particularly to ask whether river water quality is reversible or not." (p. 423)	empirical, ecology, meta-analysis	schematic degradation-recovery model	"If the water quality is practically irreversible, it is not helpful to use historical references as the management goal for the water quality standard, and a dilemma appears." (p. 428)	yes	no	meta-analysis of empirical evidence

16	147<br>D.3	Matthews, H. D. & Solomon, S. Irreversible Does Not Mean Unavoidable. Science 340, 438–439 (2013)	irreversibility as the key criterion for evaluation of climate change	warming from past emissions	1000 y timescale of irreversible warming from current CO_2emissions	no	no	schematic times series on showing how climate system responds to CO_2	“Thus, although the CO_2-induced warming O2 already present on our planet—the cumulative result of past emissions—is irreversible, any further increase in CO_2-induced warming is entirely the result of current CO_2 emissions.” (p. 438)	yes	no	perspective
17	149<br>A.2	McNeall, D., Halloran, P. R., Good, P. & Betts, R. A. Analyzing abrupt and nonlinear climate changes and their impacts. WIREs Climate Change 2, 663–686 (2011).	ir/reversibility as a criterion for assessment of abrupt and non-linear changes	change; state; tipping point	differs by system, but often 1000 years<br>general	no	dynamic system theory/nonlinear dynamics	3d ball-and-basin diagram, schematic and bifurcation forcing-state function	“For example, slow- or nonmoving systems such as coral reefs, or rainforests are vulnerable to passing thresholds in local climate. In such circumstances, a reversible change in the climate system (such as the disappearance of sea ice) may lead to an irreversible change in a related Earth system (e.g., the population of polar bears)." (p. 664)	yes	yes	assessment
18	167<br>B.2	Munson, S. M., Bradford, J. B. & Hultine, K. R. An Integrative Ecological Drought Framework to Span Plant Stress to Ecosystem Transformation. Ecosystems 24, 739–754 (2021).	ir/reversibility as a key category and component of an analytical framework for drought analysis; distinguishing stress from an irreversible threshold response + reversibility as a definiendum	thresholds	decadal time periods - ecosystem/plant changes reversible up to a point, but become permanent past that point;<br>local, regional, global	no	ecological drought framework	schematic functions of drought intensity and ecosystem performance	“An irreversible threshold is characterized as a critical proportion of xylem hydraulic conductivity lost resulting in hydraulic failure, conservatively the water potential at which there is 88% loss in conductivity (W88) (Domec and Gartner 2001) though a threshold can occur at lower losses in conductivity (McDowell and others 2013).” (p. 747)	yes	no	conceptual, theoretical
19	176<br>A.2	Notz, D. The future of ice sheets and sea ice: Between reversible retreat and unstoppable loss. Proceedings of the National Academy of Sciences 106, 20590–20595 (2009).	ir/reversibility as the key criterion for conceptual assessment of Artic Ice Loss	irreversible sea ice and ice sheet loss	time series inquired: 1953-2008; implied meaning: permanent<br>global, regional	no	sea ice model, earth system science	figures of ice loss	“By using conceptual arguments, we have rationalized in this section some recent results from more complex studies as to why […]; (iii) the loss of sea ice experienced during the last years is likely to be reversible if the climate were to become cooler again; and (iv) this reversibility exists only if significant amounts of sea ice still form during winter.” (p. 20593)	yes	no	conceptual review (of empirical evidence)
20	180<br>A.3	O’Keeffe, P. E. & Wieczorek, S. Tipping Phenomena and Points of No Return in Ecosystems: Beyond Classical Bifurcations. SIAM J. Appl. Dyn. Syst. 19, 2371–2402 (2020).	ir/reversibility as a key concept for tipping point theory from an applied dynamical systems perspective	tipping behaviour	no	no	dynamic system theory, bifurcation theory	tipping, bifurcation diagrams	“This framework aims to be easily accessible to applied scientists, addressing two questions of relevance: critical factors for tipping and the possibility of preventing tipping by a trend reversal.” (p. 2399)	no	no	theoretical

21	184<br>A.3	Ong, T. W. Y. & Vandermeer, J. Multiple hysteretic patterns from elementary population models. Theor Ecol 11, 433–439 (2018).	ir/reversibility as a component of the definitional extension of the definition of hysteresis	transitions	unclear, varies, but often described as permanent	no	ecology; predator-prey modelling	visualisations of critical transitions-hysteresis in predator-prey relations	“Here, we focus on the irreversibility of these transitions, formally known as hysteresis.” (p. 433)	yes	no	theoretical, modelling
22	201<br>B.3	Quick, I. & Brunotte, E. The significance of irreversible changes in natural environmental conditions for the development of geomorphological watercourse models on large rivers: A case study of the Lower Rhine (Germany). 142, 245–264 (2006).	irreversibility as a key criterion of analysis of river ecosystem responses	changes; depressions	implied meaning: permanent<br>local (Germany, North-Rine Westphalia)	“This article aims to explain the significance of irreversible, anthropogenic changes to natural environmental conditions in the development of watercourse models and typologies.” (p. 247)	geo-morphology	no	“Irreversible changes to the natural experiments exists which contribute to determine today's potential natural conditions.” (p. 247)	no	no	empirical, modelling
23	211<br>A.2	Ridley, J., Gregory, J. M., Huybrechts, P. & Lowe, J. Thresholds for irreversible decline of the Greenland ice sheet. Clim Dyn 35, 1049–1057 (2010).	ir/reversibility as a key criterion of (modelling) analysis of Greenland ice sheet decline + definiendum + conceptual innovation (“threshold of irreversibility”)	decline; thresholds; removal; elements	millennia, permanent loss;<br>regional (Greenland, Arctic)	“This raises the question of whether the decline would be reversible: would the ice sheet regrow if the climate cooled down?” (p. 1049)	climate modelling	schematic diagrams of scenarios warming along time	“Our aim is to look for a threshold of irreversibility in the size of the ice-sheet, above which it could regrow in pre-industrial CO_2, and below which it would be inevitably lost.” (p. 1050)	no	no	modelling
24	212<br>B.1	Ridley, J. K. & Hewitt, H. T. A mechanism for lack of sea ice reversibility in the Southern Ocean. Geophysical Research Letters 41, 8404–8410 (2014).	reversibility as the key criterion of analysis of Antarctic sea ice decline	decline; thresholds; removal; elements	millennia, permanent loss<br>regional (Antarctic), global	“To examine the climate reversibility in HadGEM2-ES, CO_2 concentration is ramped up from year zero of the preindustrial control, by 1% per annum, over 140 years to 4 times preindustrial level (1144 ppm).") (p. 8405)	climate modelling	time series function	“In this respect, the Arctic sea ice area and volume (not shown) are mostly reversible within the context of our experimental design.” (p. 8406)	no	no	modelling
25	226<br>D.2	Sasaki, T., Furukawa, T., Iwasaki, Y., Seto, M. & Mori, A. S. Perspectives for ecosystem management based on ecosystem resilience and ecological thresholds against multiple and stochastic disturbances. Ecological Indicators 57, 395–408 (2015).	i/reversibility as a key criterion (for an evaluation framework of thresholds)	ecological thresholds; threshold relationships between ecosystem states and disturbances	“various”, “from short-term to long-term”;<br>general	no	ecology	parameter-state function diagram, ball and basin diagram	“Ecological managers need to pay more attention to the possibility of state transitions in a target ecological system, because such changes are often irreversible, thus preventing restoration of the original system.” (p. 403)	yes	yes	review

26	234<br>A.2	Schleussner, C.-F., Divine, D. V., Donges, J. F., Miettinen, A. & Donner, R. V. Indications for a North Atlantic ocean circulation regime shift at the onset of the Little Ice Age. Clim Dyn 45, 3623–3633 (2015).	irreversibility as the key criterion for (modelling) inquiry (of a regime shift in the NAOC) + definiendum	time series	240-480y (between 1300 and 1500 and 1400 and 1600)<br>regional (North Atlantic Ocean)	"Here we test the hypothesis of a non-linear regime shift in the North Atlantic ocean circulation during the MCA–LIA transition [...] using a novel statistical test for time-irreversibility." (p. 3624)	climate modelling	data-based functions	"Since the concept of irreversibility refers to a time series, not to a specific point in time, a concrete timing of "the irreversible dynamics is not trivial." (p. 3626)	no	no	modelling
27	237<br>A.1	Sguotti, C. et al. Irreversibility of regime shifts in the North Sea. Frontiers in Marine Science 9, (2022).	irreversibility as the key category of review and modelling inquiry of regime shifts in the North Sea + definitional extension of hysteresis	regime shift, the new regime	implicit meaning: permanent (not proven as reversible in 40y)<br>regional (North Sea)	"Yet an outstanding question is whether regime changes in marine ecosystems are irreversible." (p. 1)	regime shifts; catastrophe theory	no	"We showed for the first time that the North Sea underwent a true, irreversible regime shift and that the community completely restructured after 2003" (p. 10)	yes	no	systematic review of empirical evidence; modelling
28	238<br>A.1<br>B.3	Siano, R. et al. Sediment archives reveal irreversible shifts in plankton communities after World War II and agricultural pollution. Current Biology 31, 2682-2689.e7 (2021).	irreversibility as a key criterion for characterisation of (empirical) inquiry (on coastal ecosystem and pollution)	shift	timescale over 1400 years; implicit meaning: change in post WW2 period is permanent	no	paleoecology	no	"[T]his study demonstrates the potential irreversible consequence of multiple and cumulated pollutions for themicrobial-compartment of the coastal ecosystem." (p. 2687)	no	no	empirical (field study)
29	243<br>B.1	Solomon, S., Plattner, G.-K., Knutti, R. & Friedlingstein, P. Irreversible climate change due to carbon dioxide emissions. Proceedings of the National Academy of Sciences 106, 1704–1709 (2009).	irreversibility as the key category for assessment of climate change due to CO2 emissions + definiendum (irreversible = a millennium)	climate change, warming; change, impacts; warming	a millennium; exceeding the year of the millennium in year 3000<br>global	no	climate science, climate modelling	time series; peak graphs	"This paper shows that the climate change that takes place due to increases in carbon dioxide concentration is largely irreversible for 1,000 years after emissions stop." (p. 1704)	yes	yes	empirical, modelling
30	250<br>A.2	Steffen, W. et al. Trajectories of the Earth System in the Anthropocene. Proceedings of the National Academy of Sciences 115, 8252–8259 (2018).	irreversibility as a key characteristic for analysis of Earth system pathways	pathway	2 timespans: glacial-interglacial cycle: 100,000y; implicit meaning of permanent<br>global	no	Earth system science	schematic depiction of irreversible trajectories that the Earth system might take	"(i) Is humanity at risk for pushing the system across a planetary threshold and irreversibly down a Hothouse Earth pathway?" (p. 8258)	yes	yes	analysis/persp ective

31	262<br>A.2	Tietsche, S., Notz, D., Jungclaus, J. H. & Marotzke, J. Recovery mechanisms of Arctic summer sea ice. Geophysical Research Letters 38, (2011).	irreversibility as the key characteristic for assessment of Arctic summer sea ice	change, loss	reversibility 2 years, seasonal ice loss and recovery<br>Arctic	"[T]hese experiments answer the question of whether perturbations of sea-ice cover alone are able to trigger an irreversible climate change in the Arctic". p. 4)	climate modelling	times series graph	"Our results suggest that anomalous loss of Arctic sea ice during a single summer is reversible, as the ice–albedo feedback is alleviated by large-scale recovery mechanisms." (p. 1)	no	no	modelling
32	286<br>B.1<br>B.2	Weber, S. et al. Quantifying irreversible movement in steep, fractured bedrock permafrost on Matterhorn (CH). The Cryosphere 11, 567–583 (2017).	i/reversibility as the key criterion for assessment (of rock slope stability + key category for describing empirical findings + conceptual innovation	displacement, fractures, components, kinematics	8y time series of data<br>local	"1. How can we statistically separate reversible from irreversible fracture kinematics?" (p. 570)	/	time series data	"The observed fracture kinematics usually consists of a reversible (elastic) and irreversible (plastic, creep and rupture) component."<br>(p. 568)	yes	no	empirical
33	298<br>A.1	Yaron, B., Dror, I. & Berkowitz, B. Contaminant geochemistry—a new perspective. Naturwissenschaften 97, 1–17 (2010).	irreversibility as the key research category of the review on anthropogenic chemical pollution of soil/the subsurface + definiendum	changes, retention, attribution, reduction; contaminants	'human timescale' of irreversibility, contrasted with geophysical	"In this review, we draw attention to a new perspective of contaminant geochemistry, namely, irreversible changes in the subsurface as a result of anthropogenic chemical pollution." (p. 1)	contaminant geochemistry	/	"By irreversible changes—on a human time scale—we refer to long-term, stable, and persistent transformations of subsurface structure and properties, which are also resistant to remediation procedures and to natural attenuation." (p. 2)	no	no	review
34	300<br>B.3	Zeng, L. et al. Longitudinal spread of bicomponent contaminant in wetland flow dominated by bank-wall effect. Journal of Hydrology 509, 179–187 (2014).	ir/reversibility as the key criterion in theory building on contamination of wetlands	reactions	long-time decay; parameter dependent	"This work is to investigate the behavior of longitudinal spread of bicomponent contaminant under the combined action of reversible and irreversible reactions, as well as hydraulic dispersion."<br>(p. 180)	chemical hydrology	Schematic parameter-state-functions	"The result shows that the length increases with time to reach maximum and then decreases to zero, and the duration is sensitive to the variation of $€_k$ which reflects the relative importance of irreversible reaction and lateral mass dispersion." (p. 186)	no	no	theoretical
35	320<br>A.1	Xu et al Deforestation triggering irreversible transition in Amazon hydrological cycle 2022 Environ. Res. Lett. 17 034037	irreversibility as the key concept to describe empirical findings on the transition of the Amazon rain forest	tipping points, alternate state	implied to be permanent<br>regional, global	"In this study, we show with observational evidences how deforestation induced atmospheric moisture changes interact with external water supplies from Atlantic Ocean and how these interactions may drive an irreversible transition in Amazon hydrological system." (p. 2)	ecology, hydrology	no	"Finally, the severe atmospheric desiccation in the southern and eastern Amazon cannot be compensated by enhanced water supplies from the Atlantic Ocean, indicating that the Amazon hydrological system is approaching an irreversible transition exacerbated by rapid deforestation."	yes	yes	empirical

36	332<br>A.3	Alkhayuon, H., Tyson, R. C. & Wieczorek, S. Phase tipping: how cyclic ecosystems respond to contemporary climate. Proceedings of the Royal Society A: Mathematical, Physical and Engineering Sciences 477, 20210059 (2021).	irreversibility as a key criterion for theorising on ecosystem response behaviour to climate change + definiendum	phase tipping, change	“in the long term”<br>no	n. a.	predator–prey modelling	schematic functions	“Here, we construct a general mathematical framework to analyse irreversible P-tipping from periodic base states. By ‘irreversible’ we mean that the system approaches a different state in the long term.” (p. 2)	no	no	theoretical
37	335<br>A.3	Lenton, T. M. Environmental Tipping Points. Annu. Rev. Environ. Resour. 38, 1–29 (2013).	ir/reversibility as a key component in the definitional extension of the definition of catastrophic bifurcation + definiendum	tipping points in the Earth system	different kinds of reversibility over different temporal scales<br>general	no	Earth system paradigm, bifurcation theory	schematic state-forcing functions	“Catastrophic bifurcations and some phase transitions involve irreversible changes. [...] However, other noncatastrophic bifurcations and phase transitions are reversible at the same value of the control”. (p. 6)	yes	yes	review
38	337<br>B.1<br>D.3	Li, X., Zickfeld, K., Mathesius, S., Kohfeld, K. & Matthews, J. B. R. Irreversibility of Marine Climate Change Impacts Under Carbon Dioxide Removal. Geophysical Research Letters 47, e2020GL088507 (2020).	ir/reversibility as the key criterion of inquiry of marine climate change under CO_2 overshoot and CDR scenarios	marine environment changes	geological timescale: >10,000; human timescale: decades to a century, multi-centennial	“This research aims to further investigate the reversibility of changes in ocean conditions after the implementation of net-negative CO_2 emissions using an Earth system model of intermediate complexion." (p. 2)	climate science, climate modelling	scenario maps	“These results suggest that negative CO_2 emissions are ineffective at reversing changes in the marine environment on human timescales (decades to a century), particularly following high levels of cumulative CO_2 emissions overshoot.”	yes	no	modelling
39	355<br>A.2	Yin, J., Schlesinger, M. E., Andronova, N. G., Malyshev, S. & Li, B. Is a shutdown of the thermohaline circulation irreversible? Journal of Geophysical Research: Atmospheres 111, (2006).	ir/reversibility as a key category in the modelling inquiry of the THC	shutdown of the Atlantic thermohaline circulation (THC)	decadal timescale (0-250y) and millennial timescale (0-5000y); response time of the ocean: within about 50y	“Is the shutdown of the THC irreversible?” (title)	(nonlinear) dynamical system theory	hysteresis loop/ stability diagram	“This study focuses on the reversibility/irreversibility of the THC after its shutdown, simulated by two different models.” (p. 14)	yes	no	modelling
40	367<br>A.1<br>A.3	Wu, S., Liang, Z. & Liu, Y. Quantifying the risk of irreversible degradation for ecosystems: A probabilistic method based on Bayesian inference. Ecological Indicators 107, 105621 (2019).	irreversibility as the key criterion for (stochastic) analysis (of the risk of ecosystem degradation) + conceptual innovation + definiendum	ecosystem degradation	no<br>local	“We further hypothesized an extreme scenario where ecosystems could be subject to a high risk of irreversible degradation, making the recovery extremely difficult or even impossible." (p. 2)	Bayesian inference	ecosystem state curve	“[W]e proposed a probabilistic indicator, RID, to quantify the risk of irreversible degradation. It is defined as the probability of the threshold for (T2) recovery being unattainable under real world scenarios.” (p. 2)	yes	no	empirical (case study), methodologic al

41	377<br>A.2	Swingedouw, D. et al. Early Warning from Space for a Few Key Tipping Points in Physical, Biological, and Social-Ecological Systems. Surv Geophys 41, 1237–1284 (2020).	ir/reversibility as a key category in the assessment of various tipping elements + irreversible as definiendum	change; irreversibility potential of change	decade(s) centuries, millennia, depending on the tipping element<br>regional, global	no	multidisciplinary: dynamical system theory, remote sensing/Earth observation, early warning analysis	no	“The possible irreversible characteristic of this change of state is related to hysteresis behaviour, meaning that a return to the same value of the driving parameter does not necessarily lead to a return of the system to its former state.” (p. 1238)	yes	yes	review, analysis
42	387<br>A.1<br>D.2	Gao, Y., Zhong, B., Yue, H., Wu, B. & Cao, S. A degradation threshold for irreversible loss of soil productivity: a long-term case study in China. Journal of Applied Ecology 48, 1145–1154 (2011).	irreversibility as a definiendum + conceptual innovation ('irreversible loss of soil services’) + the key concept for empirical soil assessment	irreversible loss of soil ecosystem services due to degradation	decades<br>local	no	applied ecology	figure showing recovery of vegetation on degraded soil	“[...] we refer to as the ‘irreversible loss of soil services’: when vegetation cover decreases below an ecological degradation threshold, leading to sustained degeneration of the vegetation community, erosion of the surface soil and declining soil fertility occur.” (p. 1146)	no	no	empirical study
43	398<br>A.1	Sguotti, C. et al. Stable landings mask irreversible community reorganizations in an overexploited Mediterranean ecosystem. Journal of Animal Ecology 91, 2465–2479 (2022).	reversibility as a key characteristics for empirical modelling of past marine ecosystem regime shifts	community changes	implicit meaning: permanent, empirical data over 40y<br>regional, local	“Here, we investigated whether the fish and macroinvertebrate community in the Northern Adriatic Sea, as represented by the landings at the Chioggia harbour in Italy, has undergone regime shifts and the potential of the community to reverse to earlier configurations.”<br>(p. 2466)	catastrophe theory, regime shift theory, stochastic cusp model	Schematic time series of reversibility of species recovery, and hysteresis	“We here show for the first time how the Northern Adriatic Sea fish and macroinvertebrate community, as represented by fishery landings, present nonlinear dynamics and has undergone truly discontinuous regime shifts over the last 40 years, which eventually led to a new, potentially irreversible state (due to hysteresis).”(p. 2475)	yes	no	empirical, modelling
44	402<br>B.1	Frölicher, T. L. & Joos, F. Reversible and irreversible impacts of greenhouse gas emissions in multi-century projections with the NCAR global coupled carbon cycle-climate model. Clim Dyn 35, 1439–1459 (2010).	ir/reversibility as the key category or modelling of GHG impacts on climate change	climate, ocean acidification, regional climate changes	human timescales; centennial to millennial, multi-millennia	“The primary goal of this study is to quantify the reversibility and irreversibility of impacts by 21st century greenhouse gas (GHG) emissions on the regional to continental scale […].” (p. 1439)	climate modelling	no	"We show that the influence of historical emissions and of non- CO2 agents is largely reversible on the regional scale. [...] In contrast, continued carbon emissions over the 21st century cause irreversible climate change on centennial to millennial timescales in most regions and impacts related to ocean acidification and sea level rise continue to aggravate for centuries even if emissions are stopped in year 2100.”	yes	yes	modelling

45	451<br>A.1	Ma, M. et al. Seed banks trigger ecological resilience in subalpine meadows abandoned after arable farming on the Tibetan Plateau. Ecological Applications 29, e01959 (2019).	reversibility as a key category of empirical inquiry of biodiversity loss in alpine meadows	biodiversity loss	1y to 20y	"Can the biodiversity/ecosystem could be reversible to the natural state after a 30-yr perturbation?" (p. 9)	conceptual model of vegetation change	alternative stable state theory framework of plant community assembly theory	"We determined the extent to which cessation of agricultural activities led to recovery of the degraded subalpine meadows to the natural meadow state, or whether the system had irreversibly shifted from a high-diversity to a low-diversity/degraded state." (p. 4)	no	no	empirical case study; conceptual
46	472<br>D.1	Griffiths, R. A. & Pavajeau, L. Captive Breeding, Reintroduction, and the Conservation of Amphibians. Conservation Biology 22, 852–861 (2008).	ir/reversibility as a key category for analysis of threats species are facing	threats of endangered species	no	no	conservation biology	no	“We then classified amphibian species into 1 of 4 categories: (1) no threats reversible, (2) some threats reversible, (3) all threats reversible, (4) unknown [...].” (p. 854)	no	no	review
47	491<br>B.2	Vedyushkin, M. A. Vegetation response to global warming: The role of hysteresis effect. Water Air Soil Pollut 95, 1–12 (1997).	irreversibility as definiendum + as a component of the definitional extension of the definition of hysteresis	change	no<br>global; the place under consideration	no	mathematical morphology, climate modelling data	no		no	yes	modelling
48	501<br>A.1	Albrich, K., Rammer, W. & Seidl, R. Climate change causes critical transitions and irreversible alterations of mountain forests. Global Change Biology 26, 4013–4027 (2020).	reversibility as a key criterion of research on simulated threshold response + irreversibility as the key category to characterise findings (simulated impacts of climate change on mountain forest)	climate impacts; effects; alterations	millennial timescales: <1000y; also depending on the topography<br>European Alps	“Here we quantified the resilience of these attributes to climate change, asking […] (c) whether climate-mediated changes in the ecosystem are reversible once the climate forcing is removed.” (p. 4015-6)	ecological resilience research; landscape modelling	state-driver charts	“Our results highlight that climate warming above critical thresholds can have irreversible impacts on forest ecosystems at millennial time scales.” (p. 4023)	yes	no	modelling
49	506<br>A.2	Armour, K. C., Eisenman, I., Blanchard-Wrigglesworth, E., McCusker, K. E. & Bitz, C. M. The reversibility of sea ice loss in a state-of-the-art climate model. Geophysical Research Letters 38, (2011).	reversibility as the key criterion of modelling research on sea ice loss; irreversibility as the key category to describe findings	Earth system transition: cryosphere, sea ice loss	450y-simulation<br>Arctic and Antarctic	“We test sea ice reversibility within a state-of-the-art atmosphere–ocean global climate model […].” (p. 1)	climate modelling	state-driver charts	“We find no evidence of irreversibility or multiple ice-cover states over the full range of simulated sea ice conditions between the modern climate and that with an annually ice-free Arctic Ocean.” (p. 1)	yes	no	modelling

50	509<br>A.3	Bagchi, S. et al. Quantifying long-term plant community dynamics with movement models: implications for ecological resilience. Ecological Applications 27, 1514–1528 (2017).	reversibility as a key concept for the description of findings (using a typology of changes and resilience conceptions), definiendum + as a key criterion for simulation analysis	change	reversible dynamics often required 2–3 decades<br>North America, Mount St. Helens	no	ecological resilience research; movement ecology	ball-and-basin-diagrams	"Resilience, and its interpretation. Our results also indicate that researchers and managers should use caution when referencing ecosystems as irreversibly altered, when they are perhaps just slow to recover (Bestelmeyer et al. 2013), taking longer than two to three decades (Table 4)." (p. 1525)	yes	no	simulation
51	511<br>B.1	Bartsev, S. I., Degermendzhi, A. G. & Erokhin, D. V. Principle of the worst scenario in the modelling past and future of biosphere dynamics. Ecological Modelling 216, 160–171 (2008).	irreversibility as the key category of inquiry of biosphere-climate dynamics + conceptual innovation	biosphere change; date; phenomenon	500y simulation<br>global	"Can these influences lead to irreversible negative changes in the climate–biosphere system or a global ecological catastrophe?" (p. 160)	conceptual and minimal mathematical climate modelling	scenario charts with irreversibility dates, ball-and-basin diagrams	"The experiments showed (Fig. 6) that under climate sensitivity to doubling of CO2 less than 4.5., no avalanche-like processes occur in the 'biosphere–climate' system, but some changes that are quite catastrophic in the ordinary sense can take place. However, at higher sensitivity values, irreversible changes do occur in the system; these changes are catastrophic in scientific terms and the 'irreversibility dates' may come very soon." (p. 166)	no	yes	modelling experiment
52	520<br>A.1<br>A.3	Bielski, C. H., Scholtz, R., Donovan, V. M., Allen, C. R. & Twidwell, D. Overcoming an "irreversible" threshold: A 15-year fire experiment. Journal of Environmental Management 291, 112550 (2021).	irreversibility as the key category of (experimental) inquiry, component of typology of thresholds, and the description of empirical research + irreversible as definiendum	threshold, regime shift	15y experiment<br>Loess Canyons, Nebraska, USA	"In this study, we assess whether the grassland-juniper woodland regime shift is irreversible or hysteretic by tracking herbaceous resurgence following stand-consuming extreme fire in juniper woodlands." (p. 2)	resilience theory	conceptual framework	"Grassland-juniper woodland regime shifts are hysteretic rather than irreversible, based on measures of aboveground productivity as an indicator of regime identity." (p. 4)	no	no	empirical (field experiment)
53	526<br>C	Botero, C. A., Weissing, F. J., Wright, J. & Rubenstein, D. R. Evolutionary tipping points in the capacity to adapt to environmental change. Proceedings of the National Academy of Sciences 112, 184–189 (2015).	ir/reversibility as a key criterion in modelling of evolutionary adaption due to climate change + as component of a definitional extension in the definition of phenotypic plasticity	phenotypic plasticity	"timescale of environmental variation"; time of the individual's development	no	evolutionary ecology	theoretical model (function)	"We first show that the parameter space […] is partitioned into distinct regions where a single mode of response (reversible phenotypic plasticity, irreversible phenotypic plasticity, bet-hedging, or adaptive tracking) has a clear selective advantage over all others."<br>(p. 184)	no	yes	theoretical modelling

54	527<br>B.1<br>D.3	Boucher, O. et al. Reversibility in an Earth System model in response to CO_2 concentration changes. Environ. Res. Lett. 7, 024013 (2012).	ir/reversibility as the key object of modelling assessment of CO_2 concentration changes) + definiendum of irreversibility	changes	“timescale far longer than those normally considered practical from a human perspective” (p. 2)	"The recent advent of second generation of Earth models […] allows reversibility and hysteresis effects to be examined in a self-consistent framework and for a much wider range of Earth System components than has previously been possible. The objective of this study is to provide an overview of these effects in the HadGEM2-ES model […].” (p. 2)	Earth system science, climate modelling	data functions	“In this study irreversibility implies hysteresis, but hysteresis does not necessarily imply irreversibility.” (p. 2)	yes	yes	climate modelling
55	564<br>A.1<br>A.3	Davis, J., Sim, L. & Chambers, J. Multiple stressors and regime shifts in shallow aquatic ecosystems in antipodean landscapes. Freshwater Biology 55, 5–18 (2010).	irreversibility as a key research category in conceptual assessment of regime shifts in aquatic ecosystems	changes in form of regime shifts	time lead to irreversible changes: >100y	“Has irreversible change occurred in urban wetlands and shallow lakes?”; “Has irreversible change occurred in agricultural wetlands and river pools?”) (p. 14)	critical/catastrophic regime shifts	conceptual diagram regime state - external stressor/driver	“Models depicting gradual ecological change and three types of regime shift (simple thresholds, hysteresis and irreversible changes) were examined in the context of shallow inland aquatic ecosystems (wetlands, shallow lakes and temporary river pools) in southwestern Australia subject to multiple anthropogenic impacts (hydrological change, eutrophication, salinisation and acidification).” (p. 5)	no	no	conceptual (testing of conceptual models in a case study)
56	565<br>A.1	De Faria, B. L. et al. Climate change and deforestation increase the vulnerability of Amazonian forests to post-fire grass invasion. Global Ecology and Biogeography 30, 2368–2381 (2021).	ir/reversibility as a key criterion for the inquiry and assessment of grass invasion after fire in the Amazon forests	shift, transition	recovery 20 years after medium to severe disturbances<br>Amazonian basin	“1. Are there large areas in the Amazon under threat of grass invasion and irreversible shifts to novel grass-dominated ecosystem states in response to fire?” (p. 2370)	resilience analysis; multi-methods	no	“Thus, in addition to climate change mitigation, intensive fire inhibition policies, especially in more vulnerable areas, could help to prevent irreversible shifts to grass-dominated biome states.” (p. 2376)	yes	yes	multi-methods modelling, resilience assessment
57	569<br>A.1	Dervaux, J., Mejean, A. & Brunet, P. Irreversible collective migration of cyanobacteria in eutrophic conditions. PLoS One 10, e0120906 (2015).	irreversibility as key criterion for experimental) analysis of bacteria migration	collective migration. formation	<6 hours<br>in vitro	“The approach followed in the present paper is to investigate quantitatively to what extent such thick scum can irreversibly form out of a collective migration and agglomeration, from well-controlled initial conditions compatible with environmental ones.” (p. 2)	experimental biology	no	“By carrying out systematic milliliter-scale experiments in test tubes, we investigate a range of environmental conditions (initial concentration of microorganisms, light intensity, salinity, nutrients chemical composition and concentration) and we identify those leading to an irreversible migration toward the surface in cultures of the cyanobacteria Microcystis aeruginosa PCC 7005”. (p. 2)	yes	no	laboratory experiment

58	612<br>B.2	Hugentobler, M., Aaron, J., Loew, S. & Roques, C. Hydro-Mechanical Interactions of a Rock Slope With a Retreating Temperate Valley Glacier. Journal of Geophysical Research: Earth Surface 127, e2021JF006484 (2022).	ir/reversibility as a key category in the description of monitoring findings of glacial rock slopes due to global warming + component of definitional extension of the definition of strains	damage, strain magnitude, deformation	hours to decades<br>Aletsch Gletscher	"We investigate these mechanical and hydromechanical responses and discuss the dominant drivers for reversible deformation, irreversible rock mass damage, and landslide formation on timescales ranging from hours to years." (p. 3)	geophysics	no	"While reversible strain probably is related to elastic deformation, we relate irreversible strain components with plastic deformation and progressive rock mass damage (Hugentobler et al., 2020, 2021). The differentiation between reversible and irreversible strain is not always trivial, because strain occurs in positive and negative direction and at different timescales." (p. 8)	no	no	monitoring study
59	620<br>A.1<br>A.3<br>B.3	Jamsranjav, C. et al. Applying a dryland degradation framework for rangelands: the case of Mongolia. Ecological Applications 28, 622–642 (2018).	ir/reversibility as a key category for empirical assessment of rangeland degradation due to livestock + component of the definitional extension of the definition of ecosystem degradation + irreversibility as definiendum	change, degradation	reversibility (recovery): 1y to > 1dec<br>irreversibility: permanent<br>Mongolia	"We predicted that livestock-induced changes (both reversible and irreversible) would be greatest in the wetter equilibrium rangelands of the mountain and forest steppe, least in the desert steppe, and moderate in the steppe and eastern steppe." (p. 624)	non-equilibrium rangeland dynamics theory	conceptual model (degradation framework)	"In this conceptualization of degradation, different levels of departure from reference conditions exist, some of which are reversible, and others of which denote permanent and irreversible changes in ecological structure and/or function. Once a rangeland is degraded irreversibly, its pre-degradation state may not be attained even with significant management efforts, due to changes in soil physical properties and the resulting feedbacks to plant communities (Bestelmeyer et al. 2013)." (p. 623)	yes	yes	empirical (field study); conceptual; degradation assessment
60	634<br>B.3<br>C	de Baan, L., Mutel, C. L., Curran, M., Hellweg, S. & Koellner, T. Land Use in Life Cycle Assessment: Global Characterization Factors Based on Regional and Global Potential Species Extinction. Environ. Sci. Technol. 47, 9281–9290 (2013).	ir/reversibility as the key category of modelling of land use and definiens in the concept of local, regional, global extinction	land use; extinction, impacts, damage	infinite (10^6y), recovery time >500y<br>global, regional, local	no	a species-area relationship<br>(SAR) model; Life Cycle Assessment	no	"Land use activities can contribute to local, regional, or even global species extinction, which show different degrees of reversibility." (p. 9282)	yes	no	modelling
61	714<br>B.1	Charbit, S., Paillard, D. & Ramstein, G. Amount of CO_2 emissions irreversibly leading to the total melting of Greenland. Geophysical Research Letters 35, (2008).	irreversibility of ice loss as the key criterion and category of modelling	the process of melting	centuries and millennia<br>Greenland	"This raises the question as to whether a complete melting of GIS is an irreversible process. Namely, will Greenland remain ice-free once the atmospheric CO_2 concentration will return to its present-day level?" (p. 1)	climate modelling	time series functions	"[T]he aim of the present study is to assess with appropriate tools which amount of CO_2 emissions could lead to an irreversible melting of GIS and to investigate, for the first time, whether the rate at which CO_2 is released to the atmosphere during centuries has a huge impact on the behavior of Greenland at the centennial to millennial time scale." (p. 1)	yes	yes	modelling, assessment

62	733<br>A.2<br>B.1<br>D.3	Ehlert, D. & Zickfeld, K. Irreversible ocean thermal expansion under carbon dioxide removal. Earth System Dynamics 9, 197–210 (2018).	ir/reversibility as the key category for analysis and description of findings of modelling sea level rise in relation to $C0_2$ concentration	thermal expansion, thermosteric sea level rise	not reversible on human timescales (decades to a century)<br>global	“The decline in radiative forcing induced by CDR raises the question of the reversibility of anthropogenic climate change, i.e. to what extent it is possible to revert to either a preindustrial climate or another warming target such as 2° C […] by artificially removing CO_2 from the atmosphere.” (p. 205)	Earth system science, climate modelling	time series functions	“We conclude that sea level rise from thermal expansion is not reversible even under strong decreases in atmospheric CO_2 on timescales relevant to human civilization.” (p. 207)	yes	no	modelling
63	738<br>C	Fardeeva, M. B. & Chizhikova, N. A. Features of Spatial and Temporal Dynamics of Tuberous Orchid Populations. Contemp. Probl. Ecol. 12, 71–82 (2019).	irreversibility as a key criterion in empirical analysis of plant dynamics	population changes	2005-2016 monitoring<br>implied meaning: permanent<br>Volga-Kama Natural Reserve, Kazanka River (Russia)	“The main purpose of our study was to elucidate the reasons and features of spatial and temporal dynamics of tuberous orchid populations. To do this, the following tasks were stated: to analyze the data of a long-term monitoring of the spatial and ontogenetic population structures, reveal the features of the fluctuation and irreversible dynamics, and determine the mechanisms of population stability of rare orchid species under various conditions.” (p. 72)	fluctuation and irreversible dynamics	no	“From 1996 to 2009 (after pasture cessa on lowland meadows), the population dynamics became irreversible, which reflected the extinction of the D. incarnata population because of the interspecific competition with meadow grasses (Figs. 5a–5d).” (p. 78)	no	no	empirical (field study)
64	754<br>A.2	Gregory, J. M., George, S. E. & Smith, R. S. Large and irreversible future decline of the Greenland ice sheet. The Cryosphere 14, 4299–4322 (2020).	irreversibility as the key category in the description of findings (modelling of Greenland ice loss) + conceptual innovation ("threshold of irreversibility")	damage, mass loss, commitment	tens of millennia<br>Greenland ice sheet	no	climate modelling	time series, maps of ice loss	“In order to avoid irreversible GMSLR [global-mean sea level rise], it would be necessary to restore the late 20th-century climate, in which the ice sheet was near mass balance, before the threshold is crossed.” (p. 4313)	yes	no	modelling

65	764<br>A.1<br>C	Hutchins, D. A. et al. Irreversibly increased nitrogen fixation in Trichodesmium experimentally adapted to elevated carbon dioxide. Nat Commun 6, 8155 (2015).	irreversibility as a key notion in the description of experimental laboratory research on the evolutionary responses of cyanobacterium Trichodesmium to elevated CO_2 levels	increase, shift, effects, fixation	by 2100	no	chemical biology	bar function	“[O]ur results demonstrate that this altered periodicity is an integral feature of the observed irreversible effects of long-term adaptation to elevated CO_2.” (p. 3)	yes	no	modelling
66	770<br>A.1	Khishigbayar, J. et al. Mongolian rangelands at a tipping point? Biomass and cover are stable but composition shifts and richness declines after 20 years of grazing and increasing temperatures. Journal of Arid Environments 115, 100–112 (2015).	ir/reversibility as definiens in the definition of degradation + operationalisation of the conceptual framework of ir/reversible degradation to characterize research findings	degradation, changes	“permanent”<br>30y study period<br>Mongolian rangeland, Bayankhongor province	implicit in the hypothesis that “that climate change, coupled with increasing livestock densities, would lead to increasing degradation indicators across all ecological zones [...].“ (p. 106)	grazing ecology, Non-equilibrium (NE) theory of rangeland dynamics	no	“Following Milton et al. (1994) and Whisenant (1999), we view degradation as a continuum of conditions from reversible and potentially temporary changes in biotic communities, to irreversible and permanent changes in both biotic and abiotic indicators and processes.” (p. 101)	yes	yes	empirical (field study)
67	776<br>B.2	Lenaerts, J. T. M. et al. Irreversible mass loss of Canadian Arctic Archipelago glaciers. Geophysical Research Letters 40, 870–874 (2013).	irreversibility as key criterion in the description of research findings	glacier mass loss	by 2100, "in the foreseeable future"; "persistent […] throughout the remainder of the 21st century”<br>Canadian Arctic Archipelago	no	climate modelling	time series	“More impor tantly,CAA glacier growth (SMB 0) is predicted in less than 1% of the ensemble members, considering an SMB uncertainty of 67 Gt yr–1 (Supporting Information), which makes it highly unlikely that CAA glacier mass loss will reverse in the coming century.” (p. 873)	no	no	modelling
68	799<br>B.3	Notebaert, B., Broothaerts, N. & Verstraeten, G. Evidence of anthropogenic tipping points in fluvial dynamics in Europe. Global and Planetary Change 164, 27–38 (2018).	ir/reversibility as a key criterion in analysis of fluvial style of rivers + irreversibility as a definiens of geomorphic tipping points	thresholds	> 8000y to remove anthropogenic sediments<br>the Dijle and Amblève catchments (Belgium) and the Valdaine Region (France)	no	geomorphic thresholds	illustration	“Increase in sediment delivery to the fluvial system has irreversibly altered the flood plains, and likely precludes a return to the original fluvial style.” (p. 33)	no	no	analysis of case studies

69	807<br>A.3	Ratajczak, Z. et al. The interactive effects of press/pulse intensity and duration on regime shifts at multiple scales. Ecological Monographs 87, 198–218 (2017)	reversibility as a key criterion in theoretical and modelling research	ecosystem transition	spatiotemporal heterogeneity	“[W]e argue that (1) the reversibility of ecosystem transitions could hinge upon the duration of change in drivers as much, or more than the change in press intensity […].” (p. 199)	regime shift theory	bifurcation graph	“Considering that slow regime shifts are potentially reversible over some time frame (Figs. 3–9; Hughes et al. 2013), it would be useful to identify indicators that signal the onset of slow regime shifts." (p. 215)	no	no	theoretical, modelling, experiment
70	819<br>A.1<br>B.3	Sharp, B. R. & Whittaker, R. J. The irreversible cattle-driven transformation of a seasonally flooded Australian savanna. Journal of Biogeography 30, 783–802 (2003).	irreversibility as a key notion in the description of empirical research findings on vegetation change	changes, transformation	irreversible in the short-to medium-term; transformation irreversible at least in the medium term<br>Victoria River, Angallarri River (Australia)	no	nonlinear transition modelling	diagram (demographic distribution)	“Observed changes are clearly irreversible in the short- to medium-term.” (p. 799)	yes	yes	empirical (field study, data analysis)
71	834<br>A.1<br>A.3	Van De Koppel, J. & Rietkerk, M. Herbivore regulation and irreversible vegetation change in semi-arid grazing systems. Oikos 90, 253–260 (2000).	irreversibility as a key criterion of modelling of vegetation changes in semi-arid grasslands	vegetation changes and shifts, loss	no	no	ecological alternate state modelling	conceptual parameter-state function	“This supports the notion that, in general, irreversible vegetation changes are less likely to occur in systems with dynamic herbivore populations compared to systems with constant herbivore numbers.” (p. 258)	no	no	conceptual modelling
72	840<br>C	Vestergård, M., Rønn, R. & Ekelund, F. Above–belowground interactions govern the course and impact of biological invasions. AoB PLANTS 7, plv025 (2015).	ir/reversibility as a key category for (review) analysis (of changes of invaded ecosystems)	change, invasion, effects, impacts	"long-term"<br>/	“[W]e claim that invasions fall into two broad categories. Some invasions irreversibly change pools and pathways of matter and energy in the invaded system; even if the abundance of the invader is reduced or it is completely removed, the system will not return to its former state. […] However, invasions may also be reversible, where the exotic organism dominates the system for a period, but in the longer term it either disappears, declines or its negative impact decreases.” (p. 1)	invasion ecology	no	“We discuss which features of invasive species and invaded systems determine the probability of irreversible impacts on the invaded ecosystem.” (p. 2)	no	no	review

73	844<br>B.3	Wang, Z. B. et al. Human impacts on morphodynamic thresholds in estuarine systems. Continental Shelf Research 111, 174–183 (2015).	ir/reversibility as a key criterion in analysis of the thresholds in estuarine systems + irreversibility as a definiens of geomorphological tipping points	thresholds	various: years to decades<br>Scheldt Estuary, Wadden Sea basin (Netherlands), Yangtze Estuary (China)	"Our objective is to identify and quantify where possible thresholds for morphodynamic developments [sic]. […] Such a change can sometimes be irreversible, resulting in a loss of the natural characteristics of the system." (p. 176)	geomorphology morphodynamics	no	"In some cases the morphological development becomes irreversible if a threshold is exceeded. Such a threshold is then a tipping point towards a regime shift, characterized by a different morphodynamic equilibrium state." (p 181)	no	no	empirical (field study)
74	870<br>A.2	Rosier, S. H. R. et al. The tipping points and early warning indicators for Pine Island Glacier, West Antarctica. The Cryosphere 15, 1501–1516 (2021).	irreversibility as a key notion to describe research findings in modelling of mass loss of West Antarctic Ice Sheet + conceptual innovation (differentiation between two types of irreversible changes)	loss, perturbation, response, tipping points; retreat	"longer periods of time"; "permanent; "on human timescale"<br>West Antarctica, Pine Island Glacier	no	using statistical tools from dynamical systems theory	schematic parameter-state functions; "ball on a slope" analogy/cup-and-basin-diagram	"Crossing each tipping point initiates periods of irreversible and self-sustained retreat of the grounding line (MISI) with significant contributions to global sea-level rise." (p. 1151)	no	no	conceptual; modelling
75	875<br>B.3<br>A.3	Rietkerk, M., Van Den Bosch, F. & Van De Koppel, J. Site-Specific Properties and Irreversible Vegetation Changes in Semi-Arid Grazing Systems. Oikos 80, 241 (1997).	irreversibility as a key criterion of theoretical analysis of vegetation changes	vegetation changes	implied meaning: permanent	"In this paper we will investigate which site-specific properties may lead to these changes, with the aim to elucidate the concept of resilience as applied to semi-arid grazing systems." (p. 242)	cusp catastrophe modelling, bifurcation theory	parameter-state diagrams	"Herbivory is likely to trigger discontinuous and irreversible changes in plant density on soils with a high erodibility." (p. 249)	yes	yes	theoretical, conceptual modelling
76	891<br>B.2	Feller, U. Drought stress and carbon assimilation in a warming climate: Reversible and irreversible impacts. Journal of Plant Physiology 203, 84–94 (2016).	i/reversibility as the key category of a review analysis of plant behaviour	effects, impacts, conditions, inactivation, damage, loss	/	"All activities must be integrated over space (including the various organs) and over time (including stress and recovery phases) to evaluate the overall performance of a plant (Xu et al., 2013; Bollig and Feller, 2014). Therefore the reversibility of damages is a key aspect in this context and is the focus of this review." (p. 85)	plant biology	no	"The decrease in net CO_2 assimilation during a heat period is initially reversible, but depending on the leaf temperature reached and the duration of the stress it may be only partially reversible or even irreversible […]." (p. 89)	no	no	review

77	896<br>A.1<br>D.2	Fischer, S., Henderson, D. C. & Koper, N. A resilient system: North American mixed-grass prairie responds rapidly to livestock exclusion. Biological Conservation 243, 108453 (2020).	ir/reversibility as a key criterion in empirical inquiry of grazing-induced changes	changes	4-yr data<br>National Park, Saskatchewan, Canada	"We hypothesized that, (1) if effects of livestock (cattle) grazing are reversible, then after livestock are removed, vegetation and bird communities would return to baseline levels observed prior to reintroduction of cattle grazing (hereafter referred to as pre-grazing levels)." (p. 2)	biological conservation and restoration management	parameter-state-functions	"Our results indicate that in northern mixed-grass prairies, grazing-induced changes were reversible, even at high stocking rates applied for up to four years." (p. 1)	no	no	empirical (field-based experiment)
78	903<br>B.2	Bragazza, L. A climatic threshold triggers the die-off of peat mosses during an extreme heat wave. Global Change Biology 14, 2688–2695 (2008).	irreversibility as a key criterion in empirical inquiry of desiccation of peat mosses + in the description of research findings	desiccation	absence of recovery after> 4y<br>Italian Alps	no	plant biology	no	"[...]the uneven distribution of the Sphagnum die-off can be explained by a climatic threshold of aridity which triggered an irreversible desiccation when the mean monthly P : T ratio dropped below 6.5 during the period from May to September 2003." (p. 2693)	no	no	empirical (field study)
79	924<br>A.1	Ratajczak, Z., Nippert, J. B. & Ocheltree, T. W. Abrupt transition of mesic grassland to shrubland: evidence for thresholds, alternative attractors, and regime shifts. Ecology 95, 2633–2645 (2014).	reversibility as a key criterion in empirical inquiry of regime shifts in mesic grasslands	transition, threshold process	28y data set<br>Great Plains, Kansas, USA	"In this study, we quantify multiple system dynamics to determine whether the transition of mesic grassland to shrubland, a widespread phenomenon, represents a linear reversible process, a nonlinear but reversible threshold process, or a transition between alternative attractors that is nonlinear and prone to hysteresis." (p. 2633)	regime shift theory	schematic parameter-state-functions	"This study provides the first empirical evidence that the transition between mesic grasslands and shrublands is nonlinear, representing either a threshold transition or regime shift between alternative attractors." (p. 2639)	no	no	empirical (experimental)
80	938<br>A.2	Caillet, J., Jourdain, N. C., Mathiot, P., Hellmer, H. H. & Mouginot, J. Drivers and Reversibility of Abrupt Ocean State Transitions in the Amundsen Sea, Antarctica. Journal of Geophysical Research: Oceans 128, e2022JC018929 (2023).	reversibility as the key research category + definiendum + concept in the characterisation of research findings	transitions	multi-decadal<br>Amundsen Sea, Antarctica	"(W)e analyze under which atmospheric forcing conditions warm-to-cold, cold-to-warm, and warm-to-warmer ocean transitions in the Amundsen Sea have occurred or could occur, and we test the reversibility of these transitions, that is, the presence of hysteresis." (p. 2)	climate science	data diagrams	"We conclude that all our warm-to-cold transitions in the Amundsen Sea are reversible. This also means that our description of the warm-to-cold transitions can be reverted to describe the cold-to-warm transitions." (p. 7)	no	no	modelling (empirical data based and experimental)

81	967<br>B.1<br>D.3	Mondal, S. K. et al. Hysteresis and irreversibility of global extreme precipitation to anthropogenic CO_2 emission. Weather and Climate Extremes 40, 100561 (2023).	ir/reversibility as the key category for research + definiendum+ conceptual innovation + characteristics of findings (of modelling of extreme precipitation)	precipitation change	varying; 140y<br>global: “Approximately 57%, 75%, and 76% of the global area are expected to experience irreversible change […].” (p. 5)	“This study endeavors some new aspects compared with the previous works. Notably, this study explores hysteresis and reversibility effect of extreme precipitation focusing on different climatic zones.” (p. 2)	climate modelling	spatial structure global maps	“The exposed population is prominent in South Africa and Asia. Notably, the population change effect is the principal factor in global exposure change, while it is the climate change effect over the hotspots of irreversibility.” (p. 1)	yes	yes	modelling
82	983<br>A.2	Wang, S. et al. Mechanisms and Impacts of Earth System Tipping Elements. Reviews of Geophysics 61, e2021RG000757 (2023).	ir/reversibility as definiendum + the key research category + key category for the classification of tipping elements	transition, ice sheet collapse	reversible: < a century, irreversible: < hundreds of years<br>global	no	environmental sciences	maps, functions	“The classification of these and other tipping elements as irreversible or reversible depends heavily upon the times cale of interest and the degree to which applied climate forcings are reversed. Many studies model one or multiple systems' response to reversed forcing over sufficiently long timescales to assess equilibrium stable states [...].” (p. 3)	yes	no	review
83	987<br>B.2	Feller, U. Drought stress and carbon assimilation in a warming climate: Reversible and irreversible impacts. Journal of Plant Physiology 203, 84–94 (2016).	ir/reversibility as the key category to describe experimental research findings	damage	17d drought, 11d rewatering with recovery	no	plant physiology	times series based on data	“The damage caused by a drought period of 17 days was irreversible in older leaves of *R. obtusifolius*, and those leaves died after the drought period.” (p. 78)	no	no	empirical<br>(pot experiment)
84	988<br>B.1<br>D.3	MacDougall, A. H. Reversing climate warming by artificial atmospheric carbon-dioxide removal: Can a Holocene-like climate be restored? Geophysical Research Letters 40, 5480–5485 (2013).	reversibility of the global climate as the key research criterion and notion in the description of research findings	climate warming	millennial ("very long time"); reversibility by 3000CE<br>global	“In this study, experiments are conducted using the University of Victoria Earth system climate model forced with novel future scenarios to explore the reversibility of climate warming as a response to a gradual return to preindustrial radiative forcing.” (p. 5480)	climate modelling	time series scenarios	“Due to hysteresis in the permafrost carbon pool, the quantity of carbon that must be removed from the atmosphere is larger than the quantity that was originally emitted (115–180% of original emissions). In all the reversibility simulations with a moderate climate sensitivity, a climate resembling that of the Holocene can be restored by 3000 CE.” (p. 5480)	yes	no	modelling

85	989<br>A.2<br>D.3	Wu, P., Ridley, J., Pardaens, A., Levine, R. & Lowe, J. The reversibility of CO_2 induced climate change. Clim Dyn 45, 745–754 (2015).	reversibility of the global climate as the key research criterion and notion in the description of research findings	climate change	varying across Earth system components: decades up to 1,000y<br>global	"In this paper, we systematically investigate inertias, thresholds and reversibility of the Earth's climate system to different rates of change in atmospheric CO2 concentrations." (p. 746)	Earth system science, climate modelling	time series scenarios	"The question is whether man-made climate change is reversible. Which components of the climate system are reversible and which are not? Is there a threshold beyond which changes to certain components of the climate system become irreversible? If in the future we can reduce atmospheric CO_2, what is the best path to reverse man-made climate change? These are questions that policy makers, environmental planners and societies have a vested interest in." (p. 746)	no	no	modelling
86	990<br>B.1<br>D.3	Tokarska, K. B. & Zickfeld, K. The effectiveness of net negative carbon dioxide emissions in reversing anthropogenic climate change. Environ. Res. Lett. 10, 094013 (2015).	reversibility of the global climate as the key research criterion and notion in the description of research findings	climate change	irreversible on centennial to millennial timescales; removal relevant on timescales relevant to human civilization: 2100 to 3000 CE<br>global	"The purpose of this study is to explore the reversibility of anthropogenic climate change and the carbon cycle response using a set of emission scenarios, which follow a gradual transition from a fossil-fuel driven economy to a zero-emission energy system with implementation of net negative CO_2 emissions." (p. 2)	climate modelling	time series scenarios	"In summary, our study suggests that it is possible, in principle, to revert global mean temperature to 2°C on centennial timescales after different levels of overshoot with the implementation of net negative emissions." (p. 9)	yes	no	modelling
87	992<br>A.1<br>D.2	Miller, J. E. D., Damschen, E. I., Ratajczak, Z. & Özdoğan, M. Holding the line: three decades of prescribed fires halt but do not reverse woody encroachment in grasslands. Landscape Ecol 32, 2297–2310 (2017).	reversibility as a key criterion in empirical inquiry of reverse transition	woody encroachment	75y observation<br>"long-term"<br>grasslands, Missouri, USA	"4. We hypothesized that landscapes undergoing encroachment would exhibit patterns suggestive of bistability, where internal thresholds may lead to limited reversibility of woody encroachment, even with frequent fires." (p. 2299)	restoration ecology, critical transitions	no	"This study fills a key knowledge gap because most studies on woody encroachment are either too short to test for long-term reversibility or are focused on conditions that prevent encroachment, rather than conditions under which it can be reversed (Briggs et al. 2005; Twidwell et al. 2016)." (p. 2299)	yes	no	empirical (historical photography)
88	993<br>A.2	Bouttes, N., Gregory, J. M. & Lowe, J. A. The Reversibility of Sea Level Rise. Journal of Climate 26, 2502–2513 (2013).	reversibility as the key research criterion and notion in the description of research findings	sea level rise	reversibility of thermal expansion on centennial time scales	no	climate modelling	time series scenarios, world maps	"The reversibility of thermal expansion cannot be explained without this distinction [between surface temperature and sea level], which arises because surface temperature relates to the temperature of the upper ocean only, and thermal expansion to the full depth of the ocean." (p. 2511)	yes	no	modelling

89	994<br>A.1<br>B.3<br>D.1	Porensky, L. M., Mueller, K. E., Augustine, D. J. & Derner, J. D. Thresholds and gradients in a semi-arid grassland: long-term grazing treatments induce slow, continuous and reversible vegetation change. Journal of Applied Ecology 53, 1013–1022 (2016).	reversibility as a key concept in the description of empirical research findings on possible state transitions	vegetation change	33y experiment, reversibility on decadal timescales<br>mixed-grass prairie in Wyoming, USA	no	non-equilibrium dynamics, alternative stable states theory	no	“Although our study cannot eliminate the possibility of alternative stable states in this ecosystem, our results are most consistent with a temporal gradient model in which grazing induces slow, continuous and reversible changes in plant species abundance.” (p. 1019)	no	no	empirical (experiment)
90	995<br>A.1<br>A.3	Gordon, L. J., Peterson, G. D. & Bennett, E. M. Agricultural modifications of hydrological flows create ecological surprises. Trends in Ecology & Evolution 23, 211–219 (2008).	ir/reversibility as a key category in conceptual modelling of regime shifts + a key category in the description of research findings + irreversible as definiendum	regime shifts	varying: field to sub-continent	no	catastrophic transition theory	equilibria diagrams	“Agriculture-aquatic system regime shifts occur at the watershed to river basin scales but vary from years to millennia in their reversibility.” (p. 215)	yes	yes	review, conceptual modelling
91	996<br>D.3	Schwinger, J., Asaadi, A., Steinert, N. J. & Lee, H. Emit now, mitigate later? Earth system reversibility under overshoots of different magnitudes and durations. Earth System Dynamics 13, 1641–1665 (2022).	ir/reversibility as a key category in conceptual modelling of regime shifts + as a key category in the description of research findings + reversibility as definiendum	climate change; Earth system	100y to 200y after the cessation of al emissions; 400y;<br>global	“Are there critical limits to the duration and/or magnitude of an overshoot beyond which (aspects of) climate change become irreversible? Are there tipping points, beyond which self-accelerating feedback make a return to a safe climate state impossible or at least difficult?”) (p. 1642)	Earth system science	time series scenarios	“Consistent with previous studies, we find irreversibility in permafrost carbon and deep ocean properties like seawater temperature, pH, and O2 concentrations.” (p. 1641)	yes	no	climate modelling

Reference List S2 Articles with non-substantive use of reversibility/reversible, irreversibility/irreversible (brief analysis)

Reference List S2 List of articles with non-substantive use included into short analysis. Reference list lists alphabetically articles with non-substantive use of reversibility/reversible, irreversibility/irreversible (short analysis). These articles were included in the short analysis as they did not show substantial use of ir/reversibility terminology. For each included article, the environmental domain(s), subject area and topic were identified using the thematic grid developed in the substantive analysis (A to D). In addition, the occurrences of the terms *revers* were screened and only the sentences and close textual context containing the terms, that is the statements containing ir/reversibility terminology, were charted. The categories for coding were developed inductively from the material, see Table S5b) for the coding categories.

1.
Toth, L. T., Kuffner, I. B., Stathakopoulos, A. & Shinn, E. A. A 3,000-year lag between the geological and ecological shutdown of Florida's coral reefs. *Global Change Biology* **24**, 5471–5483 (2018).
2.
McWethy, D. B. *et al.* A conceptual framework for predicting temperate ecosystem sensitivity to human impacts on fire regimes. *Global Ecology and Biogeography* **22**, 900–912 (2013).
3.
Fu, W., Moore, J. K., Primeau, F. W., Lindsay, K. & Randerson, J. T. A Growing Freshwater Lens in the Arctic Ocean With Sustained Climate Warming Disrupts Marine Ecosystem Function. *JGR Biogeosciences* **125**, e2020JG005693 (2020).
4.
Zamani, B., Koch, M. & Hodges, B. R. A potential tipping point in the thermal regime of a warm monomictic reservoir under climate change using three-dimensional hydrodynamic modeling. *Inland Waters* **11**, 315–334 (2021).
5.
Fidelus-Orzechowska, J., Strzyżowski, D., Cebulski, J. & Wrońska-Wałach, D. A Quantitative Analysis of Surface Changes on an Abandoned Forest Road in the Lejowa Valley (Tatra Mountains, Poland). *Remote Sensing* **12**, 3467 (2020).
6.
Livina, V. N. & Lenton, T. M. A recent tipping point in the Arctic sea-ice cover: abrupt and persistent increase in the seasonal cycle since 2007. *The Cryosphere* **7**, 275–286 (2013).
7.
Milner, A. M. *et al.* A regime shift from erosion to carbon accumulation in a temperate northern peatland. *Journal of Ecology* **109**, 125–138 (2021).
8.
Heide-Jørgensen, M. P. *et al.* A regime shift in the Southeast Greenland marine ecosystem. *Global Change Biology* **29**, 668–685 (2023).
9.
Good, P. *et al.* A review of recent developments in climate change science. Part I: Understanding of future change in the large-scale climate system. *Progress in Physical Geography: Earth and Environment* **35**, 281–296 (2011).
10.
Doblas-Miranda, E. *et al.* A review of the combination among global change factors in forests, shrublands and pastures of the Mediterranean Region: Beyond drought effects. *Global and Planetary Change* **148**, 42–54 (2017).
11.
Ursino, N. Above and below ground biomass patterns in arid lands. *Ecological Modelling* **220**, 1411–1418 (2009).
12.
Ratajczak, Z. *et al.* Abrupt Change in Ecological Systems: Inference and Diagnosis. *Trends in Ecology & Evolution* **33**, 513–526 (2018).
13.
Boulton, C. A., Ritchie, P. D. L. & Lenton, T. M. Abrupt changes in Great Britain vegetation carbon projected under climate change. *Global Change Biology* **26**, 4436–4448 (2020).

14.
Lohmann, J., Castellana, D., Ditlevsen, P. D. & Dijkstra, H. A. Abrupt climate change as a rate-dependent cascading tipping point. *Earth Syst. Dynam.* **12**, 819–835 (2021).
15.
Bathiany, S., Scheffer, M., Van Nes, E. H., Williamson, M. S. & Lenton, T. M. Abrupt Climate Change in an Oscillating World. *Sci Rep* **8**, 5040 (2018).
16.
Zhang, P. *et al.* Abrupt shift to hotter and drier climate over inner East Asia beyond the tipping point. *Science* **370**, 1095–1099 (2020).
17.
Chaparro Pedraza, P. C., Matthews, B., De Meester, L. & Dakos, V. Adaptive Evolution Can Both Prevent Ecosystem Collapse and Delay Ecosystem Recovery. *The American Naturalist* **198**, E185–E197 (2021).
18.
Lamentowicz, M. *et al.* Always on the tipping point – A search for signals of past societies and related peatland ecosystem critical transitions during the last 6500 years in N Poland. *Quaternary Science Reviews* **225**, 105954 (2019).
19.
Henne, P. D. *et al.* An empirical perspective for understanding climate change impacts in Switzerland. *Reg Environ Change* **18**, 205–221 (2018).
20.
Price, D. T. *et al.* Anticipating the consequences of climate change for Canada's boreal forest ecosystems. *Environ. Rev.* **21**, 322–365 (2013).
21.
Veit, R., Mcgowan, J., Ainley, D., Wahl, T. & Pyle, P. Apex marine predator declines ninety percent in association with changing oceanic climate. *Global Change Biology* **3**, 23–28 (1997).
22.
BioProtection Aotearoa, T. K. P. K. | S. of B. S., Te Whare Wānanga o Waitaha |. University of Canterbury, New Zealand *et al.* Applying ecological research to improve long-term outcomes of wilding conifer management. *NZJE* (2022) doi:10.20417/nzjecol.46.23.
23.
Mace, G. M. *et al.* Approaches to defining a planetary boundary for biodiversity. *Global Environmental Change* **28**, 289–297 (2014).
24.
Rao, M. P. *et al.* Approaching a thermal tipping point in the Eurasian boreal forest at its southern margin. *Commun Earth Environ* **4**, 247 (2023).
25.
Eslami-Andergoli, L., Dale, P. E. R., Knight, J. M. & McCallum, H. Approaching tipping points: a focussed review of indicators and relevance to managing intertidal ecosystems. *Wetlands Ecol Manage* **23**, 791–802 (2015).
26.
DeWeaver, E. T. Arctic Sea Ice Decline: Introduction. in *Arctic Sea Ice Decline: Observations, Projections, Mechanisms, and Implications* 1–5 (American Geophysical Union (AGU), 2008).
27.
Wassmann, P. & Lenton, T. M. Arctic Tipping Points in an Earth System Perspective. *AMBIO* **41**, 1–9 (2012).
28.
Sambaraju, K. R. & Côté, C. Are Climates in Canada and the United States Suitable for the European Spruce Bark Beetle, Ips typographus, and Its Fungal Associate, Endoconidiophora polonica? *Forests* **12**, 1725 (2021).
29.
Everard, M., Pinder, A. C., Raghavan, R. & Kataria, G. Are well-intended Buddhist practices an under-appreciated threat to global aquatic biodiversity? *Aquatic Conservation* **29**, 136–141 (2019).
30.
Angeler, D. G. *et al.* Assessing and managing freshwater ecosystems vulnerable to environmental change. *AMBIO* **43**, 113–125 (2014).
31.
Magliozzi, C. *et al.* Assessing invasive alien species in European catchments: Distribution and impacts. *Science of The Total Environment* **732**, 138677 (2020).
32.

Lux, K., Ashwin, P., Wood, R. & Kuehn, C. Assessing the impact of parametric uncertainty on tipping points of the Atlantic meridional overturning circulation. *Environ. Res. Lett.* **17**, 075002 (2022).
33.
Jørgensen, S. V., Hauschild, M. Z. & Nielsen, P. H. Assessment of urgent impacts of greenhouse gas emissions—the climate tipping potential (CTP). *Int J Life Cycle Assess* **19**, 919–930 (2014).
34.
Restrepo-Coupe, N. *et al.* Asymmetric response of Amazon forest water and energy fluxes to wet and dry hydrological extremes reveals onset of a local drought-induced tipping point. *Global Change Biology* **29**, 6077–6092 (2023).
35.
Xuan, L. *et al.* Bacterioplankton community responses and the potential ecological thresholds along disturbance gradients. *Science of The Total Environment* **696**, 134015 (2019).
36.
Wunderling, N., Gelbrecht, M., Winkelmann, R., Kurths, J. & Donges, J. F. Basin stability and limit cycles in a conceptual model for climate tipping cascades. *New J. Phys.* **22**, 123031 (2020).
37.
Lenton, T. M. Beyond 2°C: redefining dangerous climate change for physical systems. *WIREs Climate Change* **2**, 451–461 (2011).
38.
Bathiany, S. *et al.* Beyond bifurcation: using complex models to understand and predict abrupt climate change. *climatesystem* dzw004 (2016) doi:10.1093/climsys/dzw004.
39.
Higgins, P. A. T. Biodiversity loss under existing land use and climate change: an illustration using northern South America. *Global Ecology and Biogeography* **16**, 197–204 (2007).
40.
Howison, R. A., Olff, H., van de Koppel, J. & Smit, C. Biotically driven vegetation mosaics in grazing ecosystems: the battle between bioturbation and biocompaction. *Ecological Monographs* **87**, 363–378 (2017).
41.
Wills, T. J., Retallick, R. W. R., Greet, J. & Bennett, A. Browsing by non-native invasive sambar deer dramatically impacts forest structure. *Forest Ecology and Management* **543**, 121153 (2023).
42.
Gould, R. *et al.* Building on Spash's critiques of monetary valuation to suggest ways forward for relational values research. *Environmental Values* **33**, 139–162 (2024).
43.
Wiesmeier, M. *et al.* Carbon storage capacity of semi-arid grassland soils and sequestration potentials in northern China. *Global Change Biology* **21**, 3836–3845 (2015).
44.
Marengo, J. A. *et al.* Changes in Climate and Land Use Over the Amazon Region: Current and Future Variability and Trends. *Front. Earth Sci.* **6**, 228 (2018).
45.
Hunsicker, M. E. *et al.* Characterizing driver–response relationships in marine pelagic ecosystems for improved ocean management. *Ecological Applications* **26**, 651–663 (2016).
46.
Yao, H. Characterizing landuse changes in 1990–2010 in the coastal zone of Nantong, Jiangsu province, China. *Ocean & Coastal Management* **71**, 108–115 (2013).
47.
Matern, K. & Mansfeldt, T. Chromium Release from a COPR-Contaminated Soil at Varying Water Content and Redox Conditions. *J Environ Qual* **45**, 1259–1267 (2016).
48.
Rinawati, F., Stein, K. & Lindner, A. Climate Change Impacts on Biodiversity—The Setting of a Lingering Global Crisis. *Diversity* **5**, 114–123 (2013).
49.
Belcu, M., Stefan, D. S., Stefan, M., Untea, I. & Dancila, A. M. Climate change: some insights from mean surface temperature statistics. *International Journal of Sustainable Development & World Ecology* 1–8 (2015) doi:10.1080/13504509.2015.1084065.
50.
Hare, W. L., Cramer, W., Schaeffer, M., Battaglini, A. & Jaeger, C. C. Climate hotspots: key vulnerable regions, climate change and limits to warming. *Reg Environ Change* **11**, 1–13 (2011).
51.
Thompson, J. M. T. & Sieber, J. Climate tipping as a noisy bifurcation: a predictive technique. *IMA Journal of Applied Mathematics* **76**, 27–46 (2011).

52.
Bahn, M., Reichstein, M., Dukes, J. S., Smith, M. D. & McDowell, N. G. Climate–biosphere interactions in a more extreme world. *New Phytologist* **202**, 356–359 (2014).
53.
Toniazzo, T., Gregory, J. M. & Huybrechts, P. Climatic Impact of a Greenland Deglaciation and Its Possible Irreversibility. *Journal of Climate* **17**, 21–33 (2004).
54.
Yacine, Y., Allhoff, K. T., Weinbach, A. & Loeuille, N. Collapse and rescue of evolutionary food webs under global warming. *Journal of Animal Ecology* **90**, 710–722 (2021).
55.
Bergstrom, D. M. *et al.* Combating ecosystem collapse from the tropics to the Antarctic. *Global Change Biology* **27**, 1692–1703 (2021).
56.
Wohl, E., Gerlak, A. K., Poff, N. L. & Chin, A. Common Core Themes in Geomorphic, Ecological, and Social Systems. *Environmental Management* **53**, 14–27 (2014).
57.
Seekell, D. A., Carpenter, S. R., Pace, M. L., Bolnick, A. E. D. I. & McPeek, E. M. A. Conditional Heteroscedasticity as a Leading Indicator of Ecological Regime Shifts. *The American Naturalist* **178**, 442–451 (2011).
58.
Timofeev, I., Kosheleva, N. & Kasimov, N. Contamination of soils by potentially toxic elements in the impact zone of tungsten-molybdenum ore mine in the Baikal region: A survey and risk assessment. *Science of The Total Environment* **642**, 63–76 (2018).
59.
Domec, J.-C. *et al.* Conversion of natural forests to managed forest plantations decreases tree resistance to prolonged droughts. *Forest Ecology and Management* **355**, 58–71 (2015).
60.
Holbrook, S. J., Schmitt, R. J., Adam, T. C. & Brooks, A. J. Coral Reef Resilience, Tipping Points and the Strength of Herbivory. *Sci Rep* **6**, 35817 (2016).
61.
Kaur, T. & Sharathi Dutta, P. Critical rates of climate warming and abrupt collapse of ecosystems. *Proceedings of the Royal Society A: Mathematical, Physical and Engineering Sciences* **478**, 20220086 (2022).
62.
Dakos, V. & Bascompte, J. Critical slowing down as early warning for the onset of collapse in mutualistic communities. *Proc. Natl. Acad. Sci. U.S.A.* **111**, 17546–17551 (2014).
63.
Ma, S. *et al.* Critical transitions and ecological resilience of large marine ecosystems in the Northwestern Pacific in response to global warming. *Global Change Biology* **27**, 5310–5328 (2021).
64.
Wang, R., Dearing, J. A. & Langdon, P. G. Critical Transitions in Lake Ecosystem State May Be Driven by Coupled Feedback Mechanisms: A Case Study from Lake Erhai, China. *Water* **14**, 85 (2022).
65.
Trifilò, P., Abate, E., Petruzzellis, F., Azzarà, M. & Nardini, A. Critical water contents at leaf, stem and root level leading to irreversible drought-induced damage in two woody and one herbaceous species. *Plant Cell & Environment* **46**, 119–132 (2023).
66.
Thrush, S. F. *et al.* Cumulative stressors reduce the self-regulating capacity of coastal ecosystems. *Ecological Applications* **31**, e02223 (2021).
67.
Harvey, L. D. D. Declining Temporal Effectiveness of Carbon Sequestration: Implications for Compliance with the United National Framework Convention on Climate Change. *Climatic Change* **63**, 259–290 (2004).
68.
Palacín, C., Farias, I. & Alonso, J. C. Detailed mapping of protected species distribution, an essential tool for renewable energy planning in agroecosystems. *Biological Conservation* **277**, 109857 (2023).
69.
Lenton, T. M. & Livina, V. N. Detecting and Anticipating Climate Tipping Points. in *Extreme Events* 51–62 (American Geophysical Union (AGU), 2015).
70.
Spake, R. *et al.* Detecting Thresholds of Ecological Change in the Anthropocene. *Annual Review of Environment and Resources* **47**, 797–821 (2022).
71.

Saatchi, S. *et al.* Detecting vulnerability of humid tropical forests to multiple stressors. *One Earth* **4**, 988–1003 (2021).
72.
Forio, M. A. E. *et al.* Determining Tipping Points and Responses of Macroinvertebrate Traits to Abiotic Factors in Support of River Management. *Biology* **12**, 593 (2023).
73.
Harding, G., Griffiths, R. A. & Pavajeau, L. Developments in amphibian captive breeding and reintroduction programs. *Conservation Biology* **30**, 340–349 (2016).
74.
Janssen, P. *et al.* Divergence of riparian forest composition and functional traits from natural succession along a degraded river with multiple stressor legacies. *Science of The Total Environment* **721**, 137730 (2020).
75.
Burthe, S. J. *et al.* Do early warning indicators consistently predict nonlinear change in long-term ecological data? *Journal of Applied Ecology* **53**, 666–676 (2016).
76.
Brook, B. W., Ellis, E. C., Perring, M. P., Mackay, A. W. & Blomqvist, L. Does the terrestrial biosphere have planetary tipping points? *Trends in Ecology & Evolution* **28**, 396–401 (2013).
77.
Chen, N. & Wang, X. Driver-system state interaction in regime shifts: A model study of desertification in drylands. *Ecological Modelling* **339**, 1–6 (2016).
78.
Martínez-Vilalta, J. & Lloret, F. Drought-induced vegetation shifts in terrestrial ecosystems: The key role of regeneration dynamics. *Global and Planetary Change* **144**, 94–108 (2016).
79.
Bitencourt, B. S., Da Silva, P. G., Morato, E. F. & De Lima, Y. G. Dung beetle responses to successional stages in the Amazon rainforest. *Biodivers Conserv* **28**, 2745–2761 (2019).
80.
De Moura, R. S., Noriega, J. A., Cerboncini, R. A. S., Vaz-de-Mello, F. Z. & Klemann Junior, L. Dung beetles in a tight-spot, but not so much: Quick recovery of dung beetles assemblages after low-impact selective logging in Central Brazilian Amazon. *Forest Ecology and Management* **494**, 119301 (2021).
81.
Chen, A., Sanchez, A., Dai, L. & Gore, J. Dynamics of a producer-freeloader ecosystem on the brink of collapse. *Nat Commun* **5**, 3713 (2014).
82.
Schwörer, C., Colombaroli, D., Kaltenrieder, P., Rey, F. & Tinner, W. Early human impact (5000–3000 BC) affects mountain forest dynamics in the A lps. *Journal of Ecology* **103**, 281–295 (2015).
83.
Lenton, T. M. Early warning of climate tipping points. *Nature Clim Change* **1**, 201–209 (2011).
84.
Doncaster, C. P. *et al.* Early warning of critical transitions in biodiversity from compositional disorder. *Ecology* **97**, 3079–3090 (2016).
85.
Wilkinson, G. M. *et al.* Early warning signals precede cyanobacterial blooms in multiple whole-lake experiments. *Ecological Monographs* **88**, 188–203 (2018).
86.
Wang, C., Wang, Z.-H. & Sun, L. Early-Warning Signals for Critical Temperature Transitions. *Geophysical Research Letters* **47**, e2020GL088503 (2020).
87.
Horita, J., Iwasa, Y. & Tachiki, Y. Eco-evolutionary dynamics may show an irreversible regime shift, illustrated by salmonids facing climate change. *Theor Ecol* **14**, 345–357 (2021).
88.
Klaus, J., Monk, W. A., Zhang, L. & Hannah, D. M. Ecohydrological interactions during drought. *Ecohydrology* **15**, e2456 (2022).
89.
Gałka, B., Novak, A., Novak, M., Vinogradov, D. & Polechoński, R. Ecological Parameters of Water Bodies in the Northern Part of the Upper Volga Region with River Flow Regulations. *Water* **13**, 3586 (2021).
90.
Huang, H., Tu, C. & D’Odorico, P. Ecosystem complexity enhances the resilience of plant-pollinator systems. *One Earth* **4**, 1286–1296 (2021).

91.
Plagányi, É. *et al.* Ecosystem modelling provides clues to understanding ecological tipping points. *Mar. Ecol. Prog. Ser.* **512**, 99–113 (2014).
92.
DeBoer, J. A., Thoms, M. C. & Delong, M. D. Ecosystem Response Through a Resilience Lens: Do Differences in the Illinois River Over 150 Y Indicate Regime Shifts? *JGR Biogeosciences* **127**, e2021JG006553 (2022).
93.
Cortina, J. *et al.* Ecosystem structure, function, and restoration success: Are they related? *Journal for Nature Conservation* **14**, 152–160 (2006).
94.
Dakos, V. *et al.* Ecosystem tipping points in an evolving world. *Nat Ecol Evol* **3**, 355–362 (2019).
95.
Suz, L. M., Bidartondo, M. I., van der Linde, S. & Kuyper, T. W. Ectomycorrhizas and tipping points in forest ecosystems. *New Phytologist* **231**, 1700–1707 (2021).
96.
Dakos, V. & Hastings, A. Editorial: special issue on regime shifts and tipping points in ecology. *Theor Ecol* **6**, 253–254 (2013).
97.
Brook, B. W., Ellis, E. C. & Buettel, J. C. Effective Conservation Science: Data Not Dogma. in *What is the evidence for planetary tipping points?* (eds. Kareiva, P., Marvier, M. & Silliman, B.) vol. 1 (Oxford University Press, 2017).
98.
Dessu, S. B., Price, R. M., Troxler, T. G. & Kominoski, J. S. Effects of sea-level rise and freshwater management on long-term water levels and water quality in the Florida Coastal Everglades. *Journal of Environmental Management* **211**, 164–176 (2018).
99.
Olesen, J. M. Ego network analysis of the trophic structure of an island land bird through 300 years of climate change and invaders. *Ecology and Evolution* **12**, e8916 (2022).
100.
Wu, Y. *et al.* Emerging water pollution in the world's least disturbed lakes on Qinghai-Tibetan Plateau. *Environmental Pollution* **272**, 116032 (2021).
101.
Santos, M. J. Encroachment of upland Mediterranean plant species in riparian ecosystems of southern Portugal. *Biodivers Conserv* **19**, 2667–2684 (2010).
102.
Bridgewater, P., Higgs, E. S., Hobbs, R. J. & Jackson, S. T. Engaging with novel ecosystems. *Frontiers in Ecology and the Environment* **9**, 423–423 (2011).
103.
Kim, S.-H., Kim, D. E., Lee, H., Jung, S. & Lee, W.-H. Ensemble evaluation of the potential risk areas of yellow-legged hornet distribution. *Environ Monit Assess* **193**, 601 (2021).
104.
Várbíró, G. *et al.* Environmental filtering and limiting similarity as main forces driving diatom community structure in Mediterranean and continental temporary and perennial streams. *Science of The Total Environment* **741**, 140459 (2020).
105.
Kilic, S., Evrendilek, F., Berberoglu, S. & Demirkesen, A. C. Environmental Monitoring of Land-Use and Land-Cover Changes in a Mediterranean Region of Turkey. *Environ Monit Assess* **114**, 157–168 (2006).
106.
Gonçalves, A. M. M., Rocha, C. P., Marques, J. C. & Gonçalves, F. J. M. Enzymes as useful biomarkers to assess the response of freshwater communities to pesticide exposure – A review. *Ecological Indicators* **122**, 107303 (2021).
107.
Zhang, H., Wang, Q., Zhang, W., Havlin, S. & Gao, J. Estimating comparable distances to tipping points across mutualistic systems by scaled recovery rates. *Nat Ecol Evol* **6**, 1524–1536 (2022).
108.
Rietkerk, M. *et al.* Evasion of tipping in complex systems through spatial pattern formation. *Science* **374**, eabj0359 (2021).
109.

Gillson, L. Evidence of a tipping point in a southern African savanna? *Ecological Complexity* **21**, 78–86 (2015).
110.
Rindi, L., Dal Bello, M. & Benedetti-Cecchi, L. Experimental evidence of spatial signatures of approaching regime shifts in macroalgal canopies. *Ecology* **99**, 1709–1715 (2018).
111.
Williams, J. W., Blois, J. L. & Shuman, B. N. Extrinsic and intrinsic forcing of abrupt ecological change: case studies from the late Quaternary. *Journal of Ecology* **99**, 664–677 (2011).
112.
Ridolfi, L., Laio, F. & D'Odorico, P. Fertility Island Formation and Evolution in Dryland Ecosystems. *Ecology and Society* **13**, (2008).
113.
Dwomoh, F. K. & Wimberly, M. C. Fire regimes and forest resilience: alternative vegetation states in the West African tropics. *Landscape Ecol* **32**, 1849–1865 (2017).
114.
Bickford, D., Ng, T. H., Qie, L., Kudavidanage, E. P. & Bradshaw, C. J. A. Forest Fragment and Breeding Habitat Characteristics Explain Frog Diversity and Abundance in Singapore. *Biotropica* **42**, 119–125 (2010).
115.
Au, J. *et al.* Forest productivity recovery or collapse? Model-data integration insights on drought-induced tipping points. *Global Change Biology* **29**, 5652–5665 (2023).
116.
Reyer, C. P. O. *et al.* Forest resilience and tipping points at different spatio-temporal scales: approaches and challenges. *Journal of Ecology* **103**, 5–15 (2015).
117.
Alados, C. L. *et al.* Fractal analysis of plant spatial patterns: a monitoring tool for vegetation transition shifts. *Biodivers Conserv* **14**, 1453–1468 (2005).
118.
Kovač, Ž. & Sathyendranath, S. Fragility of marine photosynthesis. *Front. Mar. Sci.* **9**, 963395 (2022).
119.
Zhao, S. *et al.* Frequent locally absent rings indicate increased threats of extreme droughts to semi-arid Pinus tabuliformis forests in North China. *Agricultural and Forest Meteorology* **308–309**, 108601 (2021).
120.
Krause, S., Lewandowski, J., Dahm, C. N. & Tockner, K. Frontiers in real-time ecohydrology – a paradigm shift in understanding complex environmental systems. *Ecohydrology* **8**, 529–537 (2015).
121.
Duarte, C. M. Global change and the future ocean: a grand challenge for marine sciences. *Front. Mar. Sci.* **1**, (2014).
122.
Wall, D. H. Global Change Tipping Points: Above- and Below-Ground Biotic Interactions in a Low Diversity Ecosystem. *Philosophical Transactions: Biological Sciences* **362**, 2291–2306 (2007).
123.
Ling, S. D. *et al.* Global regime shift dynamics of catastrophic sea urchin overgrazing. *Phil. Trans. R. Soc. B* **370**, 20130269 (2015).
124.
Dirzo, R. & Raven, P. H. Global State of Biodiversity and Loss. *Annu. Rev. Environ. Resour.* **28**, 137–167 (2003).
125.
Wunderling, N. *et al.* Global warming overshoots increase risks of climate tipping cascades in a network model. *Nat. Clim. Chang.* **13**, 75–82 (2023).
126.
Goreau, T. J. F. & Hayes, R. L. Global warming triggers coral reef bleaching tipping point. *Ambio* **50**, 1137–1140 (2021).
127.
Siero, E. *et al.* Grazing Away the Resilience of Patterned Ecosystems. *The American Naturalist* **193**, 472–480 (2019).
128.
Kath, J. *et al.* Groundwater decline and tree change in floodplain landscapes: Identifying non-linear threshold responses in canopy condition. *Global Ecology and Conservation* **2**, 148–160 (2014).
129.

Yang, X. *et al.* Groundwater sapping as the cause of irreversible desertification of Hunshandake Sandy Lands, Inner Mongolia, northern China. *Proceedings of the National Academy of Sciences* **112**, 702–706 (2015).
130.
Sardanyés, J., Piñero, J. & Solé, R. Habitat loss-induced tipping points in metapopulations with facilitation. *Population Ecology* **61**, 436–449 (2019).
131.
Muthukrishnan, R. *et al.* Harnessing NEON to evaluate ecological tipping points: Opportunities, challenges, and approaches. *Ecosphere* **13**, e3989 (2022).
132.
Jiang, J., Hastings, A. & Lai, Y.-C. Harnessing tipping points in complex ecological networks. *J. R. Soc. Interface.* **16**, 20190345 (2019).
133.
Heistermann, M. HESS Opinions: A planetary boundary on freshwater use is misleading. *Hydrol. Earth Syst. Sci.* **21**, 3455–3461 (2017).
134.
Duffy, K. A. *et al.* How close are we to the temperature tipping point of the terrestrial biosphere? *Sci Adv* **7**, eaay1052 (2021).
135.
Nikolaidis, N. P. Human impacts on soils: Tipping points and knowledge gaps. *Applied Geochemistry* **26**, S230–S233 (2011).
136.
Ochoa, C. F., Baldwin, E. M., Casarín, R. S. & Martínez, G. R. Hydro-morphologic Revision of the Cuautla Channel at Nayarit, Mexico. *CLEAN Soil Air Water* **40**, 920–925 (2012).
137.
Faassen, E. J. *et al.* Hysteresis in an experimental phytoplankton population. *Oikos* **124**, 1617–1623 (2015).
138.
Mengel, M. & Levermann, A. Ice plug prevents irreversible discharge from East Antarctica. *Nature Clim Change* **4**, 451–455 (2014).
139.
Brugger, S. O. *et al.* Ice records provide new insights into climatic vulnerability of Central Asian forest and steppe communities. *Global and Planetary Change* **169**, 188–201 (2018).
140.
Di Pane, J. *et al.* Identification of tipping years and shifts in mesozooplankton community structure using multivariate analyses: a long-term study in southern North Sea. *ICES Journal of Marine Science* fsad071 (2023) doi:10.1093/icesjms/fsad071.
141.
Ciemer, C., Winkelmann, R., Kurths, J. & Boers, N. Impact of an AMOC weakening on the stability of the southern Amazon rainforest. *Eur. Phys. J. Spec. Top.* **230**, 3065–3073 (2021).
142.
Bickford, D., Howard, S. D., Ng, D. J. J. & Sheridan, J. A. Impacts of climate change on the amphibians and reptiles of Southeast Asia. *Biodivers Conserv* **19**, 1043–1062 (2010).
143.
Graham, C. T. & Harrod, C. Implications of climate change for the fishes of the British Isles. *Journal of Fish Biology* **74**, 1143–1205 (2009).
144.
Van Looy, K., Lejeune, M. & Verbeke, W. Indicators and mechanisms of stability and resilience to climatic and landscape changes in a remnant calcareous grassland. *Ecological Indicators* **70**, 498–506 (2016).
145.
Wunderling, N., Donges, J. F., Kurths, J. & Winkelmann, R. Interacting tipping elements increase risk of climate domino effects under global warming. *Earth System Dynamics* **12**, 601–619 (2021).
146.
Kerns, B. K. *et al.* Invasive grasses: A new perfect storm for forested ecosystems? *Forest Ecology and Management* **463**, 117985 (2020).
147.
Gaertner, M. *et al.* Invasive plants as drivers of regime shifts: identifying high-priority invaders that alter feedback relationships. *Diversity and Distributions* **20**, 733–744 (2014).
148.
Ritchie, P., Karabacak, Ö. & Sieber, J. Inverse-square law between time and amplitude for crossing tipping thresholds. *Proc. R. Soc. A.* **475**, 20180504 (2019).

149.
Mendez, A. & Farazmand, M. Investigating climate tipping points under various emission reduction and carbon capture scenarios with a stochastic climate model. *Proceedings of the Royal Society A: Mathematical, Physical and Engineering Sciences* **477**, (2021).
150.
Clayton, K. M. & Schmutz, J. K. Is the decline of Burrowing Owls Speotyto cunicularia in prairie Canada linked to changes in Great Plains ecosystems? *Bird Conservation International* **9**, 163–185 (1999).
151.
Newton, B. & Spence, C. JAMES BUTTLE REVIEW: A resilience framework for physical hydrology. *Hydrological Processes* **37**, e14926 (2023).
152.
Wang, B. *et al.* Keystone taxa of water microbiome respond to environmental quality and predict water contamination. *Environmental Research* **187**, 109666 (2020).
153.
Saade, C., Fronhofer, E. A., Pichon, B. & Kéfi, S. Landscape Structure Affects Metapopulation-Scale Tipping Points. *The American Naturalist* **202**, E17–E30 (2023).
154.
Loreto, F. & Centritto, M. Leaf carbon assimilation in a water-limited world. *Plant Biosystems - An International Journal Dealing with all Aspects of Plant Biology* **142**, 154–161 (2008).
155.
Clark, G. F. *et al.* Light-driven tipping points in polar ecosystems. *Global Change Biology* **19**, 3749–3761 (2013).
156.
Koch, F., Tietjen, B., Tielbörger, K. & Allhoff, K. T. Livestock management promotes bush encroachment in savanna systems by altering plant–herbivore feedback. *Oikos* **2023**, e09462 (2023).
157.
Wu, H. *et al.* Long-term habitat loss in a lightly-disturbed population of the Indo-Pacific humpback dolphin, Sousa chinensis. *Aquatic Conservation* **27**, 1198–1208 (2017).
158.
Boulton, C. A. Looking to the (far) future of climate projection. *Global Change Biology* **28**, 346–348 (2022).
159.
Bellard, C., Bernery, C. & Leclerc, C. Looming extinctions due to invasive species: Irreversible loss of ecological strategy and evolutionary history. *Global Change Biology* **27**, 4967–4979 (2021).
160.
Génin, A., Lee, S. R., Berlow, E. L., Ostoja, S. M. & Kéfi, S. Mapping hotspots of potential ecosystem fragility using commonly available spatial data. *Biological Conservation* **241**, 108388 (2020).
161.
Zhao, H. *et al.* Mapping the distribution of invasive tree species using deep one-class classification in the tropical montane landscape of Kenya. *ISPRS Journal of Photogrammetry and Remote Sensing* **187**, 328–344 (2022).
162.
Lessmann, J., Muñoz, J. & Bonaccorso, E. Maximizing species conservation in continental E cuador: a case of systematic conservation planning for biodiverse regions. *Ecology and Evolution* **4**, 2410–2422 (2014).
163.
Westman, W. E. Measuring the Inertia and Resilience of Ecosystems. *BioScience* **28**, 705–710 (1978).
164.
Stanford, B., Jones, H. & Zavaleta, E. Meta-analysis of the effects of upstream land cover on stream recovery. *Conservation Biology* **33**, 351–360 (2019).
165.
Breidenbach, A. *et al.* Microbial functional changes mark irreversible course of Tibetan grassland degradation. *Nat Commun* **13**, 2681 (2022).
166.
Summers, P. T., Elsworth, C. W., Dow, C. F. & Suckale, J. Migration of the Shear Margins at Thwaites Glacier: Dependence on Basal Conditions and Testability Against Field Data. *JGR Earth Surface* **128**, e2022JF006958 (2023).
167.
Gang, C. *et al.* Modeling the dynamics of distribution, extent, and NPP of global terrestrial ecosystems in response to future climate change. *Global and Planetary Change* **148**, 153–165 (2017).

168.
André, J., Haddon, M. & Pecl, G. T. Modelling climate-change-induced nonlinear thresholds in cephalopod population dynamics. *Global Change Biology* **16**, 2866–2875 (2010).
169.
Yiou, P. & Viovy, N. Modelling forest ruin due to climate hazards. *Earth Syst. Dynam.* **12**, 997–1013 (2021).
170.
Barnard, P. L. *et al.* Multiple climate change-driven tipping points for coastal systems. *Sci Rep* **11**, 15560 (2021).
171.
Hughes, T. P., Carpenter, S., Rockström, J., Scheffer, M. & Walker, B. Multiscale regime shifts and planetary boundaries. *Trends in Ecology & Evolution* **28**, 389–395 (2013).
172.
Thom, D. Natural disturbances as drivers of tipping points in forest ecosystems under climate change – implications for adaptive management. *Forestry* **96**, 305–315 (2023).
173.
Holme, P. & Rocha, J. C. Networks of climate change: connecting causes and consequences. *Appl Netw Sci* **8**, 10 (2023).
174.
Bush, M. B. New and Repeating Tipping Points: The Interplay of Fire, Climate Change, and Deforestation in Neotropical Ecosystems. *annals* **105**, 393–404 (2020).
175.
Unger, N. & Pan, J. L. New Directions: Enduring ozone. *Atmospheric Environment* **55**, 456–458 (2012).
176.
Huang, H., Zinnert, J. C., Wood, L. K., Young, D. R. & D'Odorico, P. Non-linear shift from grassland to shrubland in temperate barrier islands. *Ecology* **99**, 1671–1681 (2018).
177.
Brisset, E. *et al.* Non-reversible geosystem destabilisation at 4200 cal. BP: Sedimentological, geochemical and botanical markers of soil erosion recorded in a Mediterranean alpine lake. *The Holocene* **23**, 1863–1874 (2013).
178.
McGLATHERY, K. J. *et al.* Nonlinear Dynamics and Alternative Stable States in Shallow Coastal Systems. *Oceanography* **26**, 220–231 (2013).
179.
Sieber, J. & Thompson, J. M. T. Nonlinear softening as a predictive precursor to climate tipping. *Philosophical Transactions: Mathematical, Physical and Engineering Sciences* **370**, 1205–1227 (2012).
180.
Eisenman, I. & Wettlaufer, J. S. Nonlinear threshold behavior during the loss of Arctic sea ice. *Proceedings of the National Academy of Sciences* **106**, 28–32 (2009).
181.
Lindegren, M., Blenckner, T. & Stenseth, N. C. Nutrient reduction and climate change cause a potential shift from pelagic to benthic pathways in a eutrophic marine ecosystem. *Global Change Biology* **18**, 3491–3503 (2012).
182.
Guinotte, J. M. & Fabry, V. J. Ocean Acidification and Its Potential Effects on Marine Ecosystems. *Annals of the New York Academy of Sciences* **1134**, 320–342 (2008).
183.
Stranne, C. & Björk, G. On the Arctic Ocean ice thickness response to changes in the external forcing. *Clim Dyn* **39**, 3007–3018 (2012).
184.
Cantin, G., Delahaye, B. & Funatsu, B. M. On the degradation of forest ecosystems by extreme events: Statistical Model Checking of a hybrid model. *Ecological Complexity* **53**, 101039 (2023).
185.
Sirota, J., Baiser, B., Gotelli, N. J. & Ellison, A. M. Organic-matter loading determines regime shifts and alternative states in an aquatic ecosystem. *Proc. Natl. Acad. Sci. U.S.A.* **110**, 7742–7747 (2013).
186.
Rosser, A. M. & Mainka, S. A. Overexploitation and Species Extinctions. *Conservation Biology* **16**, 584–586 (2002).
187.

Llope, M. *et al.* Overfishing of top predators eroded the resilience of the Black Sea system regardless of the climate and anthropogenic conditions: BLACK SEA SHIFTS MODELLING. *Global Change Biology* **17**, 1251–1265 (2011).
188.
Ritchie, P. D. L., Clarke, J. J., Cox, P. M. & Huntingford, C. Overshooting tipping point thresholds in a changing climate. *Nature* **592**, 517–523 (2021).
189.
Brovkin, V. *et al.* Past abrupt changes, tipping points and cascading impacts in the Earth system. *Nat. Geosci.* **14**, 550–558 (2021).
190.
Chen, Y., Kolokolnikov, T., Tzou, J. & Gai, C. Patterned vegetation, tipping points, and the rate of climate change. *European Journal of Applied Mathematics* **26**, 945–958 (2015).
191.
Otero, X. L. *et al.* Phosphorus in seagull colonies and the effect on the habitats. The case of yellow-legged gulls (Larus michahellis) in the Atlantic Islands National Park (Galicia-NW Spain). *Science of The Total Environment* **532**, 383–397 (2015).
192.
Faith, D. P. Phylogenetic diversity, functional trait diversity and extinction: avoiding tipping points and worst-case losses. *Phil. Trans. R. Soc. B* **370**, 20140011 (2015).
193.
Levermann, A. *et al.* Potential climatic transitions with profound impact on Europe: Review of the current state of six 'tipping elements of the climate system'. *Climatic Change* **110**, 845–878 (2012).
194.
Gaucherel, C. & Moron, V. Potential stabilizing points to mitigate tipping point interactions in Earth's climate: POTENTIAL STABILIZING POINTS IN EARTH'S CLIMATE. *Int. J. Climatol.* **37**, 399–408 (2017).
195.
Weissmann, H. & Shnerb, N. M. Predicting catastrophic shifts. *Journal of Theoretical Biology* **397**, 128–134 (2016).
196.
THOMPSON, J. M. T. & SIEBER, J. Predicting climate tipping as a noisy bifurcation: a review. *International Journal of Bifurcation and Chaos* (2011) doi:10.1142/S0218127411028519.
197.
Sunny, E. M., Balakrishnan, J. & Kurths, J. Predicting climatic tipping points. *Chaos: An Interdisciplinary Journal of Nonlinear Science* **33**, 021101 (2023).
198.
Nacci, D. E., Champlin, D., Coiro, L., McKinney, R. & Jayaraman, S. Predicting the occurrence of genetic adaptation to dioxinlike compounds in populations of the estuarine fish Fundulus heteroclitus. *Environ Toxicol Chem* **21**, 1525–1532 (2002).
199.
Cooke, B. J. & Carroll, A. L. Predicting the risk of mountain pine beetle spread to eastern pine forests: Considering uncertainty in uncertain times. *Forest Ecology and Management* **396**, 11–25 (2017).
200.
Guo, H., Cai, R. & Tan, H. Projected harmful algal bloom frequency in the Yangtze River Estuary and adjacent waters. *Marine Environmental Research* **183**, 105832 (2023).
201.
Volery, L., Jatavallabhula, D., Scillitani, L., Bertolino, S. & Bacher, S. Ranking alien species based on their risks of causing environmental impacts: A global assessment of alien ungulates. *Global Change Biology* **27**, 1003–1016 (2021).
202.
Norby, R. J., Childs, J., Hanson, P. J. & Warren, J. M. Rapid loss of an ecosystem engineer: Sphagnum decline in an experimentally warmed bog. *Ecology and Evolution* **9**, 12571–12585 (2019).
203.
Fensham, R. J. *et al.* Rarity or decline: Key concepts for the Red List of Australian eucalypts. *Biological Conservation* **243**, 108455 (2020).
204.
Arumugam, R., Chandrasekar, V. K. & Senthilkumar, D. V. Rate-induced tipping and regime shifts in a spatial ecological system. *Eur. Phys. J. Spec. Top.* **230**, 3221–3227 (2021).
205.
Polyakov, I. V. *et al.* Recent oceanic changes in the Arctic in the context of long-term observations. *Ecological Applications* **23**, 1745–1764 (2013).

206.
Chahouri, A., Elouahmani, N. & Ouchene, H. Recent progress in marine noise pollution: A thorough review. *Chemosphere* **291**, 132983 (2022).
207.
Kaufman, D. S. *et al.* Recent Warming Reverses Long-Term Arctic Cooling. *Science* **325**, 1236–1239 (2009).
208.
Jordan, T. E. *et al.* Recommendations for Interdisciplinary Study of Tipping Points in Natural and Social Systems. *Eos, Transactions American Geophysical Union* **91**, 143–144 (2010).
209.
Molina, M. *et al.* Reducing abrupt climate change risk using the Montreal Protocol and other regulatory actions to complement cuts in CO2 emissions. *Proceedings of the National Academy of Sciences* **106**, 20616–20621 (2009).
210.
Brandt, J. S., Haynes, M. A., Kuemmerle, T., Waller, D. M. & Radeloff, V. C. Regime shift on the roof of the world: Alpine meadows converting to shrublands in the southern Himalayas. *Biological Conservation* **158**, 116–127 (2013).
211.
Alibakhshi, S., Groen, T., Rautiainen, M. & Naimi, B. Remotely-Sensed Early Warning Signals of a Critical Transition in a Wetland Ecosystem. *Remote Sensing* **9**, 352 (2017).
212.
Gao, S. *et al.* Research progress on habitat connectivity in coastal waters: A review. *Ecohydrology* **16**, e2479 (2023).
213.
Nanda, A. V. V. *et al.* Resilience and Adaptive Capacity of the Swan Coastal Plain Wetlands. *Front. Water* **3**, 754564 (2021).
214.
Vasilakopoulos, P. & Marshall, C. T. Resilience and tipping points of an exploited fish population over six decades. *Global Change Biology* **21**, 1834–1847 (2015).
215.
Rolando, J. L. *et al.* Restoration and resilience to sea level rise of a salt marsh affected by dieback events. *Ecosphere* **14**, e4467 (2023).
216.
Serreze, M. C. Rethinking the sea-ice tipping point. *Nature* **471**, 47–48 (2011).
217.
Filbee-Dexter, K. & Wernberg, T. Rise of Turfs: A New Battlefront for Globally Declining Kelp Forests. *BioScience* **68**, 64–76 (2018).
218.
Bhandary, S., Deb, S. & Sharathi Dutta, P. Rising temperature drives tipping points in mutualistic networks. *R. Soc. open sci.* **10**, 221363 (2023).
219.
Chen, N., Jayaprakash, C., Yu, K. & Guttal, V. Rising Variability, Not Slowing Down, as a Leading Indicator of a Stochastically Driven Abrupt Transition in a Dryland Ecosystem. *The American Naturalist* **191**, E1–E14 (2018).
220.
Lohmann, J. & Ditlevsen, P. D. Risk of tipping the overturning circulation due to increasing rates of ice melt. *Proc. Natl. Acad. Sci. U.S.A.* **118**, e2017989118 (2021).
221.
Jaiswal, D. & Pandey, J. River ecosystem resilience risk index: A tool to quantitatively characterize resilience and critical transitions in human-impacted large rivers. *Environmental Pollution* **268**, 115771 (2021).
222.
Guttal, V., Jayaprakash, C. & Tabbaa, O. P. Robustness of early warning signals of regime shifts in time-delayed ecological models. *Theor Ecol* **6**, 271–283 (2013).
223.
Heidari, M. Role of Natural Flocculation in Eliminating Toxic Metals. *Arch Environ Contam Toxicol* **76**, 366–374 (2019).
224.
Falk, D. A., Watts, A. C. & Thode, A. E. Scaling Ecological Resilience. *Front. Ecol. Evol.* **7**, 275 (2019).

225.
Kéfi, S., Saade, C., Berlow, E. L., Cabral, J. S. & Fronhofer, E. A. Scaling up our understanding of tipping points. *Philosophical Transactions of the Royal Society B: Biological Sciences* **377**, 20210386 (2022).
226.
Spasojevic, M. J. *et al.* Scaling up the diversity–resilience relationship with trait databases and remote sensing data: the recovery of productivity after wildfire. *Global Change Biology* **22**, 1421–1432 (2016).
227.
Clarke, J. J., Huntingford, C., Ritchie, P. D. L. & Cox, P. M. Seeking more robust early warning signals for climate tipping points: the Ratio of Spectra method (ROSA). *Environ. Res. Lett.* (2023) doi:10.1088/1748-9326/acbc8d.
228.
McGuire, A. D. *et al.* Sensitivity of the carbon cycle in the Arctic to climate change. *Ecological Monographs* **79**, 523–555 (2009).
229.
Hiddink, J. G., Valanko, S., Delargy, A. J. & van Denderen, P. D. Setting thresholds for good ecosystem state in marine seabed systems and beyond. *ICES Journal of Marine Science* **80**, 698–709 (2023).
230.
Keane, A., Krauskopf, B. & Lenton, T. M. Signatures Consistent with Multifrequency Tipping in the Atlantic Meridional Overturning Circulation. *Phys Rev Lett* **125**, 228701 (2020).
231.
Skelton, A., Kirchner, N. & Kockum, I. Skewness of Temperature Data Implies an Abrupt Change in the Climate System Between 1985 and 1991. *Geophysical Research Letters* **47**, e2020GL089794 (2020).
232.
Wang, L., D'Odorico, P., Manzoni, S., Porporato, A. & Macko, S. Soil carbon and nitrogen dynamics in southern African savannas: the effect of vegetation-induced patch-scale heterogeneities and large scale rainfall gradients. *Climatic Change* **94**, 63–76 (2009).
233.
Padmanabhan, E., Eswaran, H. & Reich, P. F. Soil carbon stocks in Sarawak, Malaysia. *Science of The Total Environment* **465**, 196–204 (2013).
234.
Gómez Ortiz, A. M., Okada, E., Bedmar, F. & Costa, J. L. Sorption and desorption of glyphosate in Mollisols and Ultisols soils of Argentina. *Enviro Toxic and Chemistry* **36**, 2587–2592 (2017).
235.
Nijp, J. J. *et al.* Spatial early warning signals for impending regime shifts: A practical framework for application in real-world landscapes. *Global Change Biology* **25**, 1905–1921 (2019).
236.
Weijer, W. *et al.* Stability of the Atlantic Meridional Overturning Circulation: A Review and Synthesis. *JGR Oceans* **124**, 5336–5375 (2019).
237.
Zinnert, J. C. *et al.* State changes: insights from the U.S. Long Term Ecological Research Network. *Ecosphere* **12**, e02014 (2021).
238.
Bathiany, S. *et al.* Statistical indicators of Arctic sea-ice stability – prospects and limitations. *The Cryosphere* **10**, 1631–1645 (2016).
239.
Fan, J. *et al.* Statistical physics approaches to the complex Earth system. *Physics Reports* **896**, 1–84 (2021).
240.
Miehe, G., Miehe, S., Kaiser, K., Liu, J. & Zhao, X. Status and dynamics of the Kobresia pygmaea ecosystem on the Tibetan plateau. *Ambio* **37**, 272–279 (2008).
241.
Hazarika, L. P., Baruah, D. & Dutta, R. Status, Distribution and Conservation Threats of Endangered Cetacean Platanista gangetica gangetica Roxb. in Subansiri River, Northeastern India. *Nature Environment and Pollution Technology* **9**, (2010).
242.
Steffen, W. *et al.* Stratigraphic and Earth System approaches to defining the Anthropocene. *Earth's Future* **4**, 324–345 (2016).
243.

Khodzher, T. V. *et al.* Study of Aerosol Nano- and Submicron Particle Compositions in the Atmosphere of Lake Baikal During Natural Fire Events and Their Interaction with Water Surface. *Water Air Soil Pollut* **232**, 266 (2021).
244.
Ward, P. L. Sulfur dioxide initiates global climate change in four ways. *Thin Solid Films* **517**, 3188–3203 (2009).
245.
Velasco, J. A. *et al.* Synergistic impacts of global warming and thermohaline circulation collapse on amphibians. *Commun Biol* **4**, 1–7 (2021).
246.
Schaphoff, S., Reyer, C. P. O., Schepaschenko, D., Gerten, D. & Shvidenko, A. Tamm Review: Observed and projected climate change impacts on Russia's forests and its carbon balance. *Forest Ecology and Management* **361**, 432–444 (2016).
247.
Liu, T. *et al.* Teleconnections among tipping elements in the Earth system. *Nat. Clim. Chang.* **13**, 67–74 (2023).
248.
Hari, M. & Tyagi, B. Terrestrial carbon cycle: tipping edge of climate change between the atmosphere and biosphere ecosystems. *Environ. Sci.: Atmos.* **2**, 867–890 (2022).
249.
Laurance, W. F. *et al.* The 10 Australian ecosystems most vulnerable to tipping points. *Biological Conservation* **144**, 1472–1480 (2011).
250.
Hou, X. *et al.* The alpine meadow around the mining areas on the Qinghai-Tibetan Plateau will degenerate as a result of the change of dominant species under the disturbance of open-pit mining. *Environmental Pollution* **254**, 113111 (2019).
251.
Princiotta, F. T. The Climate Mitigation Challenge-Where Do We Stand? *Journal of the Air & Waste Management Association* **71**, 1234–1250 (2021).
252.
McPherson, G. R., Sirmacek, B. K., Massa, J. R., Kallfelz, W. & Vinuesa, R. The commonly overlooked environmental tipping points. *Results in Engineering* **18**, 101118 (2023).
253.
Wilcox, J. L. & Martin, A. P. The devil's in the details: genetic and phenotypic divergence between artificial and native populations of the endangered pupfish (Cyprinodon diabolis). *Animal Conservation* **9**, 316–321 (2006).
254.
Schellnhuber, H. J. & Martin, M. A. The Earth System and Climate Science: Understanding a Very Complex Entity. in *Encyclopedia of Ecology* 35–41 (Elsevier, 2019).
255.
Weller, S. G. *et al.* The effects of introduced ungulates on native and alien plant species in an island ecosystem: Implications for change in a diverse mesic forest in the Hawaiian Islands. *Forest Ecology and Management* **409**, 518–526 (2018).
256.
Steffen, W. *et al.* The emergence and evolution of Earth System Science. *Nat Rev Earth Environ* **1**, 54–63 (2020).
257.
Michaeli, E., Ivanová, M. & Koco, Š. The evaluation of anthropogenic impact on the ecological stability of landscape. *J Environ Biol* **36 Spec No**, 1–7 (2015).
258.
Meireles, R. C., Lopes, L. E., Brito, G. R. & Solar, R. The future of suitable habitats of an endangered Neotropical grassland bird: A path to extinction? *Ecology and Evolution* **13**, e9802 (2023).
259.
Kanellopoulos, T. D. *et al.* The impact of flash-floods on the adjacent marine environment: the case of Mandra and Nea Peramos (November 2017), Greece. *J Coast Conserv* **24**, 56 (2020).
260.
Spencer, K. L. *et al.* The impact of pre-restoration land-use and disturbance on sediment structure, hydrology and the sediment geochemical environment in restored saltmarshes. *Science of The Total Environment* **587–588**, 47–58 (2017).

261.
Ohashi, H. *et al.* The Impact of Sika Deer on Vegetation in Japan: Setting Management Priorities on a National Scale. *Environmental Management* **54**, 631–640 (2014).
262.
Härer, A., Torres-Dowdall, J. & Meyer, A. The imperiled fish fauna in the Nicaragua Canal zone. *Conservation Biology* **31**, 86–95 (2017).
263.
Turney, C. & Fogwill, C. The implications of the recently recognized mid-20th century shift in the Earth system. *The Anthropocene Review* **9**, 403–410 (2022).
264.
Klamt, A.-M., Jensen, H. S., Mortensen, M. F., Schreiber, N. & Reitzel, K. The importance of catchment vegetation for alkalinity, phosphorus burial and macrophytes as revealed by a recent paleolimnological study in a soft water lake. *Science of The Total Environment* **580**, 1097–1107 (2017).
265.
Okin, G. S., Sala, O. E., Vivoni, E. R., Zhang, J. & Bhattachan, A. The Interactive Role of Wind and Water in Functioning of Drylands: What Does the Future Hold? *BioScience* **68**, 670–677 (2018).
266.
Jørgensen, S. V., Hauschild, M. Z. & Nielsen, P. H. The potential contribution to climate change mitigation from temporary carbon storage in biomaterials. *Int J Life Cycle Assess* **20**, 451–462 (2015).
267.
Synodinos, A. D. *et al.* The rate of environmental change as an important driver across scales in ecology. *Oikos* **2023**, e09616 (2023).
268.
Tyagi, K. & Kumar, M. The resilience of Indian Western Himalayan forests to regime shift: Are they reaching towards no return point? *Ecological Informatics* **69**, 101644 (2022).
269.
De Oliveira, A. C. P., Nunes, A., Rodrigues, R. G. & Branquinho, C. The response of plant functional traits to aridity in a tropical dry forest. *Science of The Total Environment* **747**, 141177 (2020).
270.
Ellingsen, K. E. *et al.* The rise of a marine generalist predator and the fall of beta diversity. *Global Change Biology* **26**, 2897–2907 (2020).
271.
Latty, T. & Dakos, V. The risk of threshold responses, tipping points, and cascading failures in pollination systems. *Biodivers Conserv* **28**, 3389–3406 (2019).
272.
Wilk, P., Orlińska-Woźniak, P. & Gębala, J. The river absorption capacity determination as a tool to evaluate state of surface water. *Hydrol. Earth Syst. Sci.* **22**, 1033–1050 (2018).
273.
Alfaro, R. I. *et al.* The role of forest genetic resources in responding to biotic and abiotic factors in the context of anthropogenic climate change. *Forest Ecology and Management* **333**, 76–87 (2014).
274.
Minor, E. S., Tessel, S. M., Engelhardt, K. A. M. & Lookingbill, T. R. The Role of Landscape Connectivity in Assembling Exotic Plant Communities: A Network Analysis. *Ecology* **90**, 1802–1809 (2009).
275.
Rose, B. E. J., Ferreira, D. & Marshall, J. The Role of Oceans and Sea Ice in Abrupt Transitions between Multiple Climate States. *Journal of Climate* **26**, 2862–2879 (2013).
276.
Jorgenson, M. T. *et al.* The Roles of Climate Extremes, Ecological Succession, and Hydrology in Repeated Permafrost Aggradation and Degradation in Fens on the Tanana Flats, Alaska. *JGR Biogeosciences* **125**, e2020JG005824 (2020).
277.
Nel, W., Hedding, D. W. & Rudolph, E. M. The sub-Antarctic islands are increasingly warming in the 21st century. *Antarctic Science* **35**, 124–126 (2023).
278.
Dickinson, W. R. The times are always changing: The Holocene saga. *GSA Bulletin* **107**, 1–7 (1995).
279.
Khamis, K., Hannah, D. M., Brown, L. E., Tiberti, R. & Milner, A. M. The use of invertebrates as indicators of environmental change in alpine rivers and lakes. *Science of The Total Environment* **493**, 1242–1254 (2014).

280.
Ciezkowski, W., Kleniewska, M. & Chormanski, J. Thermal and Optical Indices for Wetland Habitats, are They Showing the Same Thing? *IEEE J. Sel. Top. Appl. Earth Observations Remote Sensing* **13**, 3951–3957 (2020).
281.
Mildrexler, D. J. *et al.* Thermal Anomalies Detect Critical Global Land Surface Changes. *Journal of Applied Meteorology and Climatology* **57**, 391–411 (2018).
282.
Hillebrand, H., Kuczynski, L., Kunze, C., Rillo, M. C. & Dajka, J.-C. Thresholds and tipping points are tempting but not necessarily suitable concepts to address anthropogenic biodiversity change—an intervention. *Mar. Biodivers.* **53**, 43 (2023).
283.
Veblen, K. E. & Porensky, L. M. Thresholds are in the eye of the beholder: plants and wildlife respond differently to short-term cattle corrals. *Ecological Applications* **29**, e01982 (2019).
284.
Thomas, Z. A. *et al.* Tipping elements and amplified polar warming during the Last Interglacial. *Quaternary Science Reviews* **233**, 106222 (2020).
285.
Duarte, C. M. *et al.* Tipping Elements in the Arctic Marine Ecosystem. *AMBIO* **41**, 44–55 (2012).
286.
Schellnhuber, H. J. Tipping elements in the Earth System. *Proceedings of the National Academy of Sciences* **106**, 20561–20563 (2009).
287.
Braghiere, R. K. *et al.* Tipping point in North American Arctic-Boreal carbon sink persists in new generation Earth system models despite reduced uncertainty. *Environ. Res. Lett.* **18**, 025008 (2023).
288.
Jassey, V. E. J. *et al.* Tipping point in plant–fungal interactions under severe drought causes abrupt rise in peatland ecosystem respiration. *Global Change Biology* **24**, 972–986 (2018).
289.
Carrier-Belleau, C., Pascal, L., Nozais, C. & Archambault, P. Tipping points and multiple drivers in changing aquatic ecosystems: A review of experimental studies. *Limnology & Oceanography* **67**, (2022).
290.
Phillips, J. D. Tipping points in Texas rivers. *Earth Surf Processes Landf* **43**, 1768–1781 (2018).
291.
Ditlevsen, P. Tipping Points in the Climate System. in *Nonlinear and Stochastic Climate Dynamics* (eds. Franzke, C. L. E. & O'Kane, T. J.) 33–53 (Cambridge University Press, Cambridge, 2017).
292.
Lenton, T. M. Tipping points in the climate system. (2021) doi:10.1002/wea.4058.
293.
Ban, Z., Hu, X. & Li, J. Tipping points of marine phytoplankton to multiple environmental stressors. *Nat. Clim. Chang.* **12**, 1045–1051 (2022).
294.
Ditlevsen, P. D. & Johnsen, S. J. Tipping points: Early warning and wishful thinking. *Geophysical Research Letters* **37**, 2010GL044486 (2010).
295.
Nobre, C. A. & Borma, L. D. S. 'Tipping points' for the Amazon forest. *Current Opinion in Environmental Sustainability* **1**, 28–36 (2009).
296.
Lenton, T. M. Tipping positive change. *Phil. Trans. R. Soc. B* **375**, 20190123 (2020).
297.
Camarero, J. J., Gazol, A., Sangüesa-Barreda, G., Oliva, J. & Vicente-Serrano, S. M. To die or not to die: early warnings of tree dieback in response to a severe drought. *Journal of Ecology* **103**, 44–57 (2015).
298.
Tan, Y.-H. *et al.* Transcriptomic analysis reveals distinct mechanisms of adaptation of a polar picophytoplankter under ocean acidification conditions. *Marine Environmental Research* **182**, 105782 (2022).
299.

Hoover, D. L. *et al.* Traversing the Wasteland: A Framework for Assessing Ecological Threats to Drylands. *BioScience* **70**, 35–47 (2020).
300.
Wood, T. E., Cavaleri, M. A. & Reed, S. C. Tropical forest carbon balance in a warmer world: a critical review spanning microbial- to ecosystem-scale processes. *Biological Reviews* **87**, 912–927 (2012).
301.
Anantanarayanan, R. & Prasad, S. Two-hundred year changes in plant-species composition: A case study of Madras city in the coromandel coast, peninsular India. *International Journal of Ecology and Environmental Sciences* **36**, 205–214 (2010).
302.
Li, L., Jiang, E., Yin, H., Wu, K. & Dong, G. Ultrashort-term responses of riparian vegetation restoration to adjacent cycles of ecological water conveyance scheduling in a hyperarid endorheic river basin. *Journal of Environmental Management* **320**, 115803 (2022).
303.
Devisscher, T., Malhi, Y., Rojas Landívar, V. D. & Oliveras, I. Understanding ecological transitions under recurrent wildfire: A case study in the seasonally dry tropical forests of the Chiquitania, Bolivia. *Forest Ecology and Management* **360**, 273–286 (2016).
304.
Falkenmark, M., Wang-Erlandsson, L. & Rockström, J. Understanding of water resilience in the Anthropocene. *Journal of Hydrology X* **2**, 100009 (2019).
305.
Turnbull, L. *et al.* Understanding the role of ecohydrological feedbacks in ecosystem state change in drylands. *Ecohydrology* **5**, 174–183 (2012).
306.
Jager, H. I., Novello, R. C., Dale, V. H., Villnas, A. & Rose, K. A. Unnatural hypoxic regimes. *Ecosphere* **9**, e02408 (2018).
307.
Krause, B. & Farina, A. Using ecoacoustic methods to survey the impacts of climate change on biodiversity. *Biological Conservation* **195**, 245–254 (2016).
308.
Hu, Z., Dakos, V. & Rietkerk, M. Using functional indicators to detect state changes in terrestrial ecosystems. *Trends in Ecology & Evolution* **37**, 1036–1045 (2022).
309.
Lenton, T. M. *et al.* Using GENIE to study a tipping point in the climate system. *Philosophical Transactions of the Royal Society A: Mathematical, Physical and Engineering Sciences* **367**, 871–884 (2008).
310.
van Belzen, J. *et al.* Vegetation recovery in tidal marshes reveals critical slowing down under increased inundation. *Nat Commun* **8**, 15811 (2017).
311.
Whitford, W. G., De Soyza, A. G., Van Zee, J. W., Herrick, J. E. & Havstad, K. M. Vegetation, Soil, and Animal Indicators of Rangeland Health. *Environ Monit Assess* **51**, 179–200 (1998).
312.
Lamsal, P., Kumar, L., Atreya, K. & Pant, K. P. Vulnerability and impacts of climate change on forest and freshwater wetland ecosystems in Nepal: A review. *Ambio* **46**, 915–930 (2017).
313.
Marques, M., Da Costa, M. F., Mayorga, M. I. D. O. & Pinheiro, P. R. C. Water Environments: Anthropogenic Pressures and Ecosystem Changes in the Atlantic Drainage Basins of Brazil. *AMBIO: A Journal of the Human Environment* **33**, 68–77 (2004).
314.
Li, X., Song, K. & Liu, G. Wetland Fire Scar Monitoring and Its Response to Changes of the Pantanal Wetland. *Sensors* **20**, 4268 (2020).
315.
Lenton, T. M. What early warning systems are there for environmental shocks? *Environmental Science & Policy* **27**, S60–S75 (2013).
316.
Brook, B. W., Ellis, E. C. & Buettel, J. C. What is the evidence for planetary tipping points? in *Effective Conservation Science: Data Not Dogma* (eds. Kareiva, P., Marvier, M. & Silliman, B.) vol. 1 51–57 (Oxford University Press, 2018).
317.

Denning, A. S. Where Has All the Carbon Gone? *Annual Review of Earth and Planetary Sciences* **50**, 55–78 (2022).
318.
Baker, A. G. & Catterall, C. Where has all the fire gone? Quantifying the spatial and temporal extent of fire exclusion in Byron Shire, Australia. *Ecological Management & Restoration* **16**, 106–113 (2015).
319.
Johannessen, O. M. & Shalina, E. V. Will the summer sea ice in the Arctic reach a tipping point? *Atmospheric and Oceanic Science Letters* **16**, 100352 (2023).

Supplementary Material 2

Search protocol for a systematic literature search

This protocol is based on the search protocol as provided by J Hirt and T Nordhausen (original German version) as an appendix of the RefHunter information platform. Original title: Rechercheprotokoll für eine systematische Literaturrecherche; version 4.0 30 June 2022 [accessed on 10 October 2022]. The protocol template has been translated into English and slightly modified (shortened).

Content

General Information about the development of the search strategy 2
Research question(s) 2
Inclusion and exclusion criteria (eligibility criteria) 2
1 Principle of search Strategy 3
2 Determination of the search components 4
3 Selection of databases 4
4 Keywords 5
5 Identification of subject headings 6
6 Development of the final search string 7
8 Conduction of the research 10
9 Documentation of the search 11
9.1 Database 1 (Web of Science) 11
9.2 Database 2 (Scopus) 11
9.3 Database 3 (GreenFILE) 11
10 Additional research options 12

Reference for the German version:

Hirt, J., Nordhausen, T. (2022). Rechercheprotokoll für eine systematische Literaturrecherche. In: Nordhausen, T., Hirt, J. RefHunter. Systematische Literaturrecherche. https://refhunter.org/research_support/rechercheprotokoll/ [accessed on 10 October 2022]

Version 4.0

30 June 2022

BY SA

General Information about the development of the search strategy

Name(s) of the person(s) developing the search strategy: Lorina Buhr

Date or period of development: 15 Nov 2022 – 14 Jan 2023

Research question(s)

(1) How is the term and concept of irreversibility and related terminology used and how it is conceptualized in scientific articles that contribute to debates in the Earth System, geo, life, and biological sciences on anthropogenic changes?

(2) What are thematic foci and conceptual linkages in scientific articles that use the concept of 'irreversibility' and related terms contributing to debates on anthropogenic changes regarding the earth, life, and nature in the Earth System, geo, life, and biological sciences?

(3) What are implicit and explicit normative aspects of the usages of the notion of irreversibility?

Inclusion and exclusion criteria (eligibility criteria)

Domain	Inclusion criteria	Exclusion criteria
**Domain 1:** Research Areas/Fields	I. Research Areas:<br>• Earth (System) sciences<br>• Geo Sciences and involved disciplines, such as applied chemistry and geophysics<br>• Ecology<br>• Biology including genetics<br>• Intersectional and multidisciplinary research fields and studies, such as Life Sciences and Agriculture, Forestry, Biodiversity Conservation, Meteorology, Atmosphere Sciences, Oceanology<br><br>II. Topics:<br>• anthropogenic climate change<br>• anthropogenic changes in the Earth system's spheres/Earth's systems<br>• human-driven environmental changes, i.e., changes in eco systems/in the biosphere, regarding biodiversity<br>• Anthropocene	I. Research Areas and Fields of<br>• Theoretical physics and chemistry<br>• Basic research in inorganic chemistry<br>• Mathematics<br>• Material Sciences<br>• Engineering<br>• Energy Sciences<br>• Pharmacology<br>• Molecular and cell biology & biochemistry<br>• Medicine<br>• Veterinary<br>• Social Sciences<br>• Humanities<br>• Human Geography<br>• Anthropology<br>• Archaeology<br><br>(= wrong research field)<br><br>II. Topics:<br>• All types of general, theoretical, nonorganic, or molecular-based branches of disciplines in the natural sciences<br>• Irreversibility demonstrated in or by mathematical, molecular, quantum or other microphysical modelling<br><br>(= wrong topic I)
**Domain 2: Specification**	III. Occurrence and Usage:<br>• Occurrence of the notion of irreversibility/irreversible/ no reversibility/non-reversible in the full text	III. Occurrence and Usage:<br>• No or unspecified occurrence of the notion of irreversibility/irreversible/ reversibility in the full text<br>• No definitional or conceptual work on tipping subjects (i.e. tipping

	• Definition, conceptualization, or substantial inquiry of (ir)reversible changes<br>• Definition, conceptualization, or substantial inquiry of (ir)reversible impacts of technological interventions<br>• Definition, conceptualization, or inquiry of tipping points/elements/events ('tipping subjects')<br><br>= conceptual and topical context according to the target research fields and topics<br>General rule for title-abstract screening (screening step 1): rather generous inclusion for full-text screening, marked with uncertainty	points/elements/events), climate change, environmental/eco system changes<br><br>(= wrong topic II)
**Domain 3: Type of publication**	IV. Types of records:<br>Published scientific articles, papers, proceeding paper, opinions, editorials, and commentary	IV. Types of records:<br>• Non-scientific or non-professional papers or statements<br>• policy briefs, policy statements<br>• unpublished texts grey literature; preprints<br><br>(= formal failure)
**Domain 4: Language**	V. Language:<br>English	V. Language:<br>Language other than English<br>(= formal failure)
**Domain 5: Period**	No limitation	/

Reasons for inclusion and exclusion criteria:

☒ The research focus is on 'applied' usages of the notion of irreversibility in the determined scientific discourses and research fields. All results from theoretical physics and chemistry, or mathematics, material sciences etc. (see the list above) are excluded as they are beyond the focus of the research questions. The same applies to Material Sciences, Engineering, Medicine, and Veterinary, they are not of interest. Results from Social Sciences and the Humanities are excluded from analysis but considered for the interpretation and discussion of the results. This is a review of the *scientific* debates; accordingly, the focus is exclusively on scientific publications; the societal, political or public debates and media are not subject matter. Thus, all types of non-scientific types of texts are excluded.

☐ No justification necessary.

1 Principle of search Strategy

Reasons for the decision:

☐ Sensitive search principle

Goal: Find as many relevant hits as possible.

State the reason or aim of the search.

☐ Precise search principle

Goal: Find the most important relevant hits as quickly as possible.

State the reason or aim of the search.

☒ Mixed form (partially sensitive and precise search strategy)

Goal: Find as many relevant hits as possible with an optimised cost/effort ratio.

Balanced research between sensitive and precise research is targeted. However, the overall accent lies on the precise research principle.

2 Determination of the search components

Determination of the the search components from the research question. If a justification for (individual) defined or non-defined search components appears necessary, this can be recorded below the table.

Search component	Name
Search component 1	irreversibility / irreversible OR tipping (concept)
Search component 2	nature (subject matter)
Search component 3	/
Search component 4	/

Reasons for the decision:

☒ The review focusses exclusively on the term and concept of irreversibility respective non-reversibility as the primary search component. Therefore, the first research componentf is restricted to terminology that refer to the concept of irreversibility. To clarify this point: the purposes of the study are (1) to identify exploratively the conceptual relations and functions of the (precise) concept and term of irreversibility and normative aspects of the usages of the term of irreversibility/irreversible/ no(n) reversibility/reversible (i.e., inflected forms of the lexical root of [ir]reversib*). Thus, (2) the study aims to reveal how the *terminology of irreversibility* is used in scientific research fields on 'nature' in the broadest sense, and what are the conceptual underpinnings of this use.

Accordingly, alternative terms and synonyms are not considered in the original research strategy. The analysis aims to identify conceptual linkages and phrases in connection with the notion of irreversibility, an illustrating example for this would be the phrase 'irreversible change(s)'. However, one exception from this strict research criteria must be made. An initial search has revealed that in the Earth System and Geo Sciences the concept of irreversibility is often used in connection with the notion of tipping points/elements/events (hereafter: 'tipping subjects'), but not always indicated as keyword or used in the title and abstract. Since the articles that deal with tipping subjects are relevant for the analysis, the research string needs to anticipate this challenge.

☐ No justification necessary.

3 Selection of databases

Determination of the databases to be searched and, if applicable, the search engines to be used to search the databases. The decision can be recorded with a justification for the selection of the respective database.

Database	Name / Reasoning
Database 1	**Web of Science** (WoS), Science Citation Index Expanded + Emerging Source Citation Index
	covers a broad range of disciplines; the chosen citation indices serve as a preliminary filter which makes the research more precise
Database 2	**Scopus (Elsevier),**
	covers a broad range of disciplines and journals, complementary to Web of Science;
Database 3	**GREENFILE**
	GreenFILE is a literature database concerned with the human impact on the environment. It covers many relevant journals that meet the research questions; it is recommended as a database complementary to the large databases of WoS and Scopus

4 Keywords

Identification of keywords and their synonyms for each search component. If a justification for (individual) selected or unselected keywords appears necessary, this can be recorded below the table.

Search component	Keywords
**Search component 1: concept**	IRREVERSIBILITY, IRREVERSIBLE,<br>NON-REVERSIBILITY, NON-REVERSIBLE, NOT REVERSIBLE<br>TIPPING
**Search component 2: nature**	CLIMATE<br>EARTH<br>PLANET<br>NATURE<br>ENVIRONMENT<br>ECOSYSTEM<br>ECOLOGY<br>LIFE<br>SPECIES<br>ATMOSPHERE<br>OCEAN

Reasons for (individual) selected or unselected keywords (reasons for selection):

☒ The review focusses exclusively on the concept and term of irreversibility respective non-reversibility.

Keyword 1: Considering the rationale to focus on irreversibility, the first research item is restricted to terms that refer to the concept directly. To clarify this point: the aim is to identify the conceptual relations and functions of the (precise) concept and term of irreversibility and normative aspects of the usage of the precise term of irreversibility/irreversible/ no(n) reversibility/reversible (and inflected forms of the lexical root of [ir]reversib*). Thus, the analysis aims to identify conceptual linkages and phrases in connection with the term of irreversibility; an illustrating example for this is the phrase ‘irreversible change(s)’. Accordingly, alternative terms and synonyms are not considered in the original research strategy. However, one exception from this strict research criteria must be made. An initial search has revealed that in the Earth System and Geo Sciences the concept of irreversibility is often used in connection with the notion of tipping points/elements/events (tipping subjects) but not always indicated as keyword or used in the title and abstract. The inclusion of “tipping” increases the database results significantly. On the one hand, the significant difference in results requires the inclusion of tipping in the search string, on the other hand the increased number of articles requires a strategy in the screening. To keep the screening manageable, only articles on tipping subjects will be included that provide theoretical and conceptual work and that refer to the concept of irreversibility. It is expected that during the title and abstract screening (screening step 1) a considerable number of the articles on tipping subjects can be removed as they do not meet the research question and inclusion criteria sufficiently. Yet, in many cases probably only the full-text screening (step 2) will allow the decision on whether to finally include or exclude an article on tipping subjects.

Sear Item 2: We looked for synonyms and notions in the sphere of ‘nature’ broadly understood. Generic items such as ‘bio*’ and ‘geo*’ caused massive and non-manageable increase in results, thus they turned out to be inapplicable.

Sear Item 2:

☐ No justification necessary.

5 Identification of subject headings

Identification of the subject terms for the keywords defined in the previous step for each search component and database. If a justification for (individual) selected or unselected keywords appears necessary, this can be recorded under the table.

Keywords	Search component	Subject terms: Web of Science	Subject terms: Scopus	Subject terms: GreenFILE
IRREVERSIBILITY<br>IRREVERSIBILITIES<br>IRREVERSIBLE<br>NO REVERSIBILITY<br>NO REVERSIBILITIES<br>NON-REVERSIBILITY<br>NON-REVERSIBLE<br>NOT REVERSIBLE<br>TIPPING	Search component 1:<br>Irreversibility (concept)	/	/	/
CLIMATE<br>EARTH<br>PLANET<br>NATURE<br>ENVIRONMENT<br>ECOSYSTEM<br>ECOLOGY<br>LIFE<br>SPECIES<br>OCEAN<br>ATHMOSPHERE	Search component 2:<br>nature (subject matter)	/	/	CLIMATOLOGY<br>CLIMATE CHANGE<br>ATMOSPHERE, EARTH TEMPERATURE<br>GEOMORPHOLOGY<br>NATURE<br>ECOLOGY<br>ECOLOGY<br>SPECIES<br>LIFE SCIENCES<br>OCEAN

Reasons for (individual) selected or unselected subject terms:

☒ Justification:

WoS: The “Web of Science Core Collection employs no controlled vocabulary or thesaurus in assigning subject terms. Natural language indexing (where every word in the title is searchable) is used.“ (https://support.clarivate.com/ScientificandAcademicResearch/s/article/Web-of-Science-Core-Collection-No-controlled-vocabulary-or-thesaurus-in-assigning-subject-terms?language=en_US) This means that the identification of subject terms is not applicable.

Scopus: The database offers subject terms from MEDLINE and EMBASE, however, these are not relevant for this study.

GreenFILE: This database uses subject terms (SU), which were used where applicable. We identified the Subject terms by research in the Green Thesaurus

☐ No justification necessary.

6 Development of the final search string

Combination of the keywords and subject terms identified in the previous two steps for each search component and database in columns to form a search string. Definition of search techniques, e.g. wildcards (e.g. truncations), phrase search (e.g. inverted commas) and search commands or search fields into which the search string is inserted (syntax). If a justification for (individual) used or unused search techniques and/or search commands or search fields appears necessary, this can be recorded under the table.

Search component	Search String: **Web of Science**	Search String: **Scopus**	Suchstring: **GreenFILE**
**Search component 1: irreversibility** (concept)	TI=(IRREVERSIB* OR (NO REVERSIB*) OR "TIPPING")) OR (KP=(IRREVERSIB* OR (NO REVERSIB*) OR "TIPPING")) OR AK=(IRREVERSIB* OR (NO REVERSIB*) OR "TIPPING"))	((TITLE (IRREVERSIB*) OR TITLE ((NO OR NON OR NOT) PRE/2 REVERSIB*) OR TITLE ("TIPPING"))) OR ((KEY (IRREVERSIB*) OR KEY ((NO OR NON OR NOT) PRE/2 REVERSIB*) OR KEY ("TIPPING")))	((TI IRREVERSIB* OR "TIPPING" OR (NO* REVERSIB*)) OR (AB IRREVERSIB* OR "TIPPING" OR (NO* REVERSIB*) OR KW IRREVERSIB* OR "TIPPING" OR (NO* REVERSIB*))
	AND	AND	AND
**Search component 2: nature** (subject matter)	TS=(CLIMAT* OR EARTH* OR PLANET* OR NATUR* OR ENVIRONMENT* OR ECOSYSTEM* OR ECOLOG* OR LIFE* OR LIV* OR SPECIES OR OCEAN* OR ATMOSPHER*)	TITLE-ABS-KEY (CLIMAT* OR EARTH* OR PLANET* OR NATUR* OR ENVIRONMENT* OR ECOSYSTEM* OR ECOLOG* OR LIFE* OR LIV* OR SPECIES OR OCEAN* OR ATMOSPHER*)	((TI CLIMAT* OR EARTH* OR PLANET* OR NATUR* OR ENVIRONMENT* OR ECOSYSTEM* OR ECOLOG* OR LIFE* OR LIV* OR SPECIES OR OCEAN* OR ATMOSPHER*) OR (AB CLIMAT* OR EARTH* OR PLANET* OR NATUR* OR ENVIRONMENT* OR ECOSYSTEM* OR ECOLOG* OR LIFE* OR LIV* OR SPECIES OR OCEAN* OR ATMOSPHER*) OR (KW CLIMAT* OR EARTH* OR PLANET* OR NATUR* OR ENVIRONMENT* OR ECOSYSTEM* OR ECOLOG* OR LIFE* OR LIV* OR SPECIES OR OCEAN* OR ATMOSPHER*) OR (SU CLIMATOLOGY OR CLIMATE CHANGE OR ATMOSPHERE OR EARTH TEMPERATURE OR GEOMORPHOLOGY OR NATURE OR ECOLOGY OR LIFE SCIENCES OR SPIECES OR OCEAN)
	AND	AND	AND
**Search component 3: Citation Index (if applicable)**	SCIENCE CITATION INDEX EXPANDED (SCI-EXPANDED)<br>EMERGING SOURCES CITATION INDEX (ESCI)	/	/
	ADDITIONALLY	AND	AND
**Search component 4: restriction to research domains and fields (if applicable)**	/	/	
	/	/	/
**Search filters (a posterior)**	ENGLISH	ENGLISH	ENGLISH

Reasons for (individual) used or unused search items and/or keywords and search fields:

☒ Webs of Science: It turned out that the terms irreversible/irreversibility and tipping produced too many and fuzzy results when used with the field operator Topic (TS). We therefore restricted the field research by limiting the items to the field operators title (TI), WoS Keyword plus (KP) and author keyword (AKP). As the terms used in the title and the (author) keywords are supposed to reflect the primary conceptual and topical focus, the limitation meets the research question more accurately. Additionally, the limitation released the research results from articles from the excluded fields significantly, thus it turned the research strategy much more efficient and precise. We tested the proximity operator offered by WoS (NEAR). However, as the use of the proximity operator (NEAR/5) showed no relevant effect compared to the phrase search "no reversib*", we decided to waive the use of the proximity operator.

Scopus: To keep consistency equivalent field operators were uses (TITLE, KEY) for the concept item and the TITLE-ABS-KEY field operator was used for the subject matter. As Scopus offers a proximity operator that searches for terms before terms, this was used for the non-reversibility item; the distance of up to two words showed the most accurate results. Therefore, we used PRE/2 as proximity operator.

☐ No justification necessary.

7 Check of the search string

In our case, the search strings were subject of discussion and investigation with two experts for systematic literature reviews from Utrecht University Library.

Review of the search string or search strings based on criteria, if possible by a person not previously involved (PRESS 2015 Guideline Evidence-Based Checklist)[1].

[…].

Correction of the search string or further consequences for the search project

Determination of the necessary or recommended corrections resulting from the review or further consequences for the search project.

The search item ‘revers*’ seemed too broad as it included term such as ‘reversal’, ‘in reverse’, which muddled the results, so we decided to shift the truncation slightly to ‘reversib*’ as named above.

[1] Modifikation nach: McGowan, J., Sampson, M., Salzwedel, D. M., Cogo, E., Foerster, V. & Lefebvre, C. (2016). PRESS Peer Review of Electronic Search Strategies: 2015 Guideline Statement. Journal of Clinical Epidemiology, 75, 40-46. Augenscheinliche Übersetzung, welche nicht durch Dritte geprüft wurde.

8 Conduction of the research

Documentation of the search filters (e.g. with regard to the inclusion and exclusion criteria for record selection) used in the search as well as any special features that arose when conducting the search in databases. If a justification for the use of search filters appears necessary, this can be recorded here.

Filter options chosen: English (as the shared working language of the international project team)

Particularities: No.

9 Documentation of the search

Documentation of the database-specific search strings with details of the search date and the search hits.

9.1 Database 1 (Web of Science)

Date: 20 January 2023 + 23 September 2023 (time filter: 2023)

Search string stored online: ☒ Yes ☐ No

#	Search string entry	Number of records
20 Jan	((TI=(IRREVERSIB* OR (NO REVERSIB*) OR "TIPPING")) OR (KP=(IRREVERSIB* OR (NO REVERSIB*) OR "TIPPING")) OR AK=(IRREVERSIB* OR (NO REVERSIB*) OR "TIPPING")) AND TS=((CLIMAT* OR EARTH* OR PLANET* OR NATUR* OR ENVIRONMENT* OR ECOSYSTEM* OR ECOLOG* OR LIFE* OR LIV* OR SPECIES OR OCEAN* OR ATMOSPHER*)	6779
23 Sept	SEE ABOVE	372

9.2 Database 2 (Scopus)

Date: 20 January 2023 + 23 September 2023 (time filter: 2023)

Search string stored online: ☒ Yes ☐ No

#	Search string entry	Number of records
	(TITLE-ABS-KEY (CLIMAT* OR EARTH* OR PLANET* OR NATUR* OR ENVIRONMENT* OR ECOSYSTEM* OR ECOLOG* OR LIFE* OR LIV* OR SPECIES OR OCEAN* OR ATMOSPHER*)) AND (((TITLE (IRREVERSIB*) OR TITLE ((NO OR NON OR NOT) PRE/2 REVERSIB*) OR TITLE ("TIPPING"))) OR ((KEY (IRREVERSIB*) OR KEY ((NO OR NON OR NOT) PRE/2 REVERSIB*) OR KEY ("TIPPING")))) AND (LIMIT-TO (LANGUAGE , "ENGLISH"))	9417
23 Sept	SEE ABOVE	462

9.3 Database 3 (GreenFILE)

Date: 20 January 2023 + 23 September 2023 (time filter: 2023)

Search string stored online: ☒ Yes ☐ No

#	Search string entry	Number of records
20 Jan	((TI IRREVERSIB* OR "TIPPING" OR (NO* REVERSIB*)) OR (KW IRREVERSIB* OR "TIPPING" OR (NO* REVERSIB*)) OR (AB IRREVERSIB* OR "TIPPING" OR (NO* REVERSIB*)) AND ((TI CLIMAT* OR EARTH* OR PLANET* OR NATUR* OR ENVIRONMENT* OR ECOSYSTEM* OR ECOLOG* OR LIFE* OR LIV* OR SPECIES OR OCEAN* OR ATMOSPHER*) OR (AB CLIMAT* OR EARTH* OR PLANET* OR NATUR* OR ENVIRONMENT* OR ECOSYSTEM* OR ECOLOG* OR LIFE* OR LIV* OR SPECIES OR OCEAN* OR ATMOSPHER*) OR (KW CLIMAT* OR EARTH* OR PLANET* OR NATUR* OR ENVIRONMENT* OR ECOSYSTEM* OR ECOLOG* OR LIFE* OR LIV* OR SPECIES OR OCEAN* OR ATMOSPHER*) OR (SU CLIMATOLOGY OR CLIMATE CHANGE OR ATMOSPHERE OR EARTH TEMPERATURE OR GEOMORPHOLOGY OR NATURE OR ECOLOGY OR LIFE SCIENCES OR SPIECES OR OCEAN))	3077
23 Sept	SEE ABOVE	168

10 Additional research options

☐ Forward citation search

Date: TT.MM.JJJJ – TT.MM.JJJJ

☐ Google Scholar
☐ Scopus
☐ Web of Science Core Collection
☐ Other: Name

Number of rounds: Number

Number of records: Number

☒ Backward citation search

Date: 27 September 2023

☒ Screening Reference Lists of included articles to identify further relevant articles
☐ Using a database:

☐ Scopus
☐ Web of Science Core Collection
☐ Other: Name

Number of rounds: 1

Number of records: 3

☐ Manuel research

[…]

☐ Contacting study authors

[…]

☐ Free web based research

[…]
